# Aging China's cardiac challenge: Projected growth of atrial arrhythmia cases and healthcare preparedness through 2045 — A global burden disease analysis

**DOI:** 10.1111/ggi.70089

**Published:** 2025-06-25

**Authors:** Junpeng Xiong, Shuwen Chen, Xiaoli Wu, Bingyin Wang, Binquan You, Ronghui Yu

**Affiliations:** ^1^ Department of Cardiology National Cardiovascular Disease Regional Center for Anhui, First Affiliated Hospital of Anhui Medical University Hefei China; ^2^ Department of Anesthesiology Second Affiliated Hospital of Anhui Medical University Hefei China; ^3^ Department of Cardiology Suzhou Kowloon Hospital, Shanghai Jiaotong University School of Medicine Suzhou China; ^4^ Department of Cardiology Luliang First People's Hospital lvliang China

**Keywords:** atrial fibrillation/flutter, China, global burden disease, prediction analysis, aging population

## Abstract

Atrial fibrillation/flutter (AF/AFL) poses significant risks of heart failure and stroke. From 1990 to 2021, China's AF/AFL cases surged from 3.20 to 10.78 million. Using Global Burden Disease (GBD) 2021 data and Bayesian age‐period‐cohort (BAPC) modeling, we analyzed trends and projected disease burden through 2045. Age‐standardized incidence and prevalence rose by 5.4% and 14.5%, respectively, while mortality and disability‐adjusted life year (DALY) rates declined by 12.2% and 3.8%. By 2045, prevalent cases may reach 23.04 million, with 1.93 million new cases, 220 000 deaths, and 4.21 million DALYs. Key risk factors include hypertension, smoking, high‐sodium diet, and obesity. Sex disparities emerged, with females showing better mortality/DALY improvements than males. Population aging and metabolic risks will drive future burden. Targeted interventions (blood pressure control, smoking cessation, sodium reduction) and sex‐/age‐specific strategies are critical. Innovations in digital health (AI monitoring) and tiered healthcare networks could mitigate risks. Prioritizing China‐specific prevention frameworks is essential to address this escalating public health challenge. **Geriatr Gerontol Int 2025; 25: 1047–1057**.

## Graphical abstract

Atrial fibrillation/flutter cases in China have tripled since 1990. Using GBD 2021 data and BAPC modeling, this study projects rising incidence and prevalence through 2045, driven by aging and metabolic risk. Targeted, China‐specific prevention and digital health strategies are urgently needed to mitigate future burden.

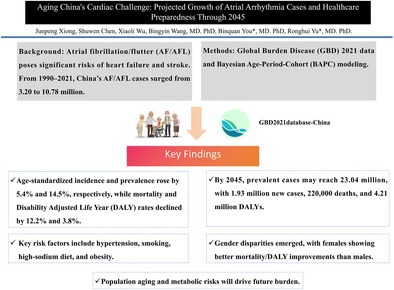

## Introduction

Atrial fibrillation/atrial flutter (AF/AFL) is one of the most common clinical arrhythmias, potentially leading to severe complications such as heart failure and ischemic stroke. It is associated with high mortality and disability rates, significantly impacting patients' physical and mental health as well as their quality of life.^1^ According to Global Burden of Disease (GBD) data, the prevalence and mortality rates of AF/AFL increased markedly worldwide from 1990 to 2021. In 2021, the number of prevalent cases reached 52.55 million, and AF/AFL‐related deaths amounted to 339 000.^2^ With the growing global population and the increasing trend of aging, the disease burden of AF/AFL is expected to rise significantly in the future.^3^, ^4^, ^5^

China has one of the fastest‐aging populations in the world. By 2040, the population aged 60 and above is projected to reach 402 million, accounting for 28% of the total population.^6^ Given that the incidence of AF/AFL is positively correlated with age, demographic shifts are likely to lead to a further surge in the number of patients in China. These profound demographic changes may significantly increase the patient population, posing dual challenges to the public health system and socio‐economic development. However, there is currently limited research on the future disease burden of AF/AFL in China, highlighting an urgent need for scientific predictions to inform the development of prevention and control strategies.

This study systematically evaluates the disease burden of AF/AFL in mainland China from 1990 to 2021, based on data from the 2021 GBD study. By integrating population aging models, the study predicts future disease trends. By uncovering the critical factors influencing the prevalence and impact of AF/AFL, this research will contribute to the development of evidence‐based policies and interventions, ultimately enhancing public health outcomes and aligning with Chinese national health priorities.

## Materials and methods

### Data sources

The primary data were obtained from the GBD 2021 study (https://ghdx.healthdata.org/gbd-2021), which provides updated epidemiological data (1990–2021) for 371 diseases and injuries across 204 countries and territories, including 811 subnational regions, alongside 88 risk factors contributing to disease burden.^7^, ^8^ All GBD estimates include 95% uncertainty intervals (UIs), calculated as the 25th and 975th ordered values from 1000 posterior distribution draws.^9^ Ethical approval for this study was acquired from the University of Washington Institutional Review Board. Study protocols and anonymized data can be accessed upon request through the Ethiopian Public Health Institute’s (EPHI) ‐ Health Metrics and Evaluation (IHME) office^10^ or the GBD 2021 online database.^11^ Data from China were sourced mainly from national institutions such as the China Disease Surveillance System and the Chinese Center for Disease Control and Prevention, including mortality and cancer registry records (https://www.cdc.gov/global-health/countries/china).

### Definitions of indicators

AF/AFL cases were identified using the International Classification of Diseases (ICD‐9 codes 427.3–427.32 and ICD‐10 codes I48–I48.92). Electrocardiograms were utilized for AF/AFL diagnosis. Disability‐adjusted life years (DALYs) were defined as the sum of years of life lost (YLLs) and years lived with disability (YLDs). YLLs were calculated by multiplying the number of deaths by the standard life expectancy at the age of death. YLDs were estimated by weighing the duration of health loss by disability severity.

Risk factors for AF/AFL were identified based on etiological pathways, prior literature, and GBD‐available risk factors.^12^, ^13^, ^14^ In GBD 2021, six risk factors—high body mass index (BMI), elevated systolic blood pressure, smoking, alcohol use, high sodium intake, and lead exposure—were linked to AF/AFL‐related mortality. Definitions of these risk factors and methods for quantifying their percentage contributions to AF/AFL‐related deaths have been published previously.

### Statistical analysis

This study aimed to systematically analyze trends in AF/AFL‐related mortality, DALYs, and disease burden in China. Age‐standardized rates (ASRs) were applied to quantify mortality and DALYs, with estimated annual percentage changes (EAPCs) calculated to assess global burden trends. ASRs were adjusted to a global standard population age distribution,^15^ enabling comparisons across populations with varying age structures. EAPCs were derived by regressing the natural logarithm of ASRs against the calendar year (model: ln(ASR) = α + βx + ε, where x = calendar year and ε = error term). EAPC trends were reported with 95% confidence intervals (CIs). An increasing trend was defined as an EAPC with a lower 95% CI >0, while a decreasing trend required an upper 95% CI <0. AF/AFL burden in China was stratified by sex and age. Linear regression models were used to evaluate current trends. Risk factor contributions to AF/AFL burden were expressed as percentages.

Using age‐period‐cohort (APC) analysis with the Norpred method, we projected AF/AFL mortality and DALYs in China from 2022 to 2045. Data inputs included GBD 1990–2021 estimates and United Nations World Population Prospects (2019 revision) demographic projections (https://population.un.org/wpp).^16^ APC models, enhanced with Bayesian approaches, addressed collinearity among age, period, and cohort effects. Bayesian inference integrated prior information and data to estimate posterior distributions via integrated nested Laplace approximation (INLA). Second‐order random walk (RW2) models were applied to smooth age, period, and cohort effects, implemented using the R packages Norpred and INLA.

All statistical analyses, data visualization, and numerical computations were performed using R software (version 4.3.1).

## Results

### AF/AFL burden by sex in China, 1990–2021

**Figure 1:**
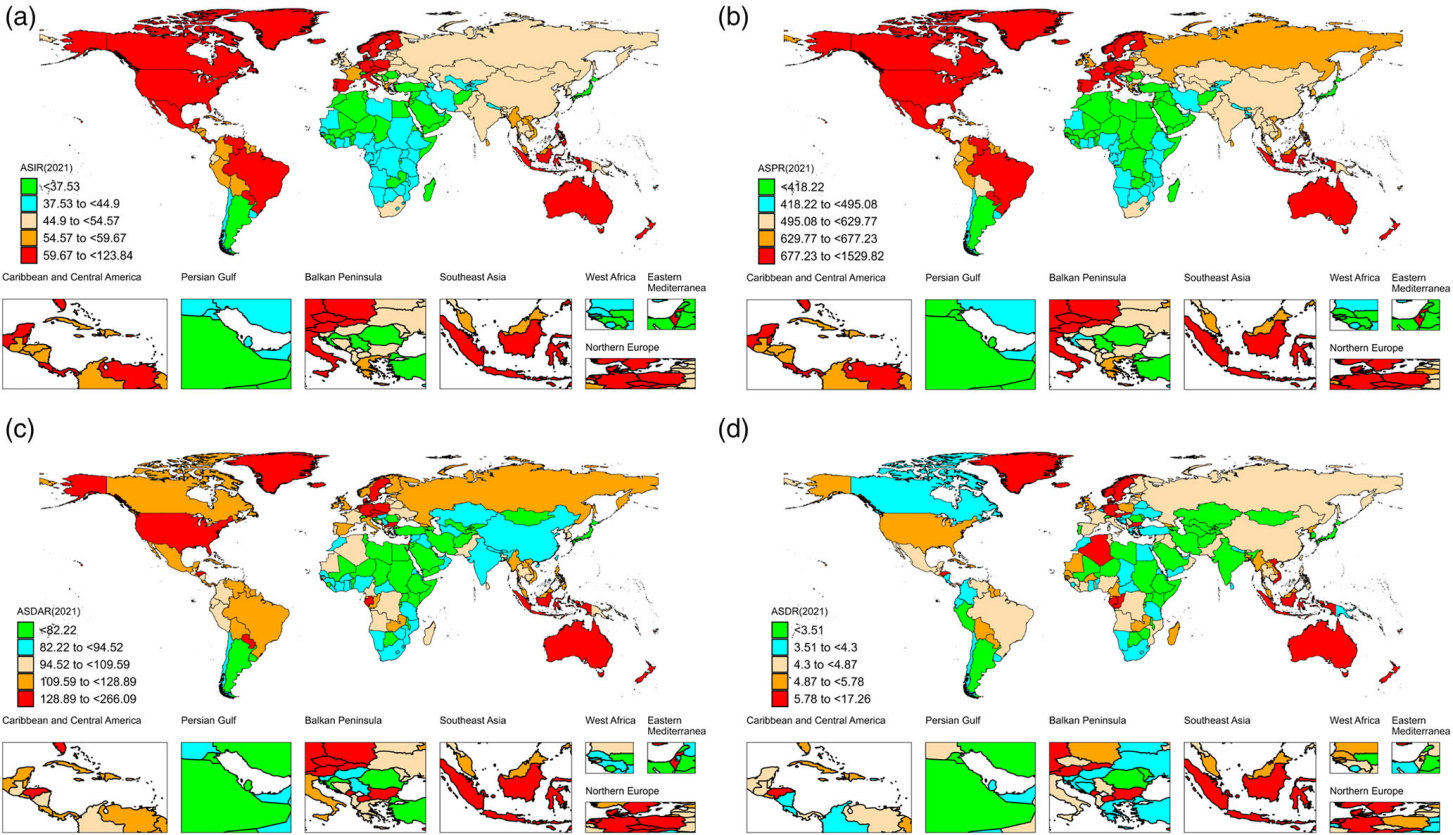
Trends in the global burden of AF/AFL in 2021. (a–d) ASIR, ASPR, ASDAR, and ASDR across 204 countries in 2021. AF/AFL, atrial fibrillation/atrial flutter; ASDAR, age‐standardized DALYs rate; ASDR, age‐standardized deaths rate; ASIR, age‐standardized incidence rate; ASPR, age‐standardized prevalence rate; DALYs, disability‐adjusted life years.

Globally, age‐standardized prevalence rates (ASPRs) of AF/AFL were 61.66 million in 1990 and 62.05 million in 2021, representing an upward trend from 1990 to 2021. In China, EAPCs were 0.16% (95% CI: 0.1%–0.22%) for age‐standardized incidence rate (ASIR) and 0.48% (95% CI: 0.38%–0.58%) for ASPR. China's age‐standardized mortality rate (ASMR) and age‐standardized DALY rate (ASDAR) were at moderate global levels, with ASMR ranging from 4.3% to 4.87%, ASPR from 495.08% to 629.77%, and ASDR from 82.22% to 94.52% (Fig. 1). Globally, age‐standardized mortality and DALY rates for AF/AFL also increased: ASMR rose from 4.24 (3.69–4.71) in 1990 to 4.36 (3.69–4.75) in 2021, while ASDR increased from 100.81 (82.82–122.62) to 101.4 (84.89–122.41).

In contrast, China demonstrated declines in ASMR and ASDR for AF/AFL: ASMR decreased from 4.93 in 1990 to 4.33 in 2021, and ASDR declined from 93.28 to 89.76. However, males experienced rising ASMR and ASDR: ASMR increased from 3.56 to 3.81, and ASDR rose from 81.96 to 89.62. Conversely, females showed significant decreases in ASMR (17.3% decrease) and ASDR (10.3% decrease) compared with males (Table 1).

**Table 1:** Numbers and age‐standardized incidence, prevalence, DALYs and deaths rates of AF/AFL in 2021, and their percentage change in ASR from 1990 to 2021, by sex

	Number of incidence cases		ASIR			Number of prevalence cases		ASPR		
	1990	2021	1990	2021	EAPC (95%UI)	1990	2021	1990	2021	EAPC (95% UI)
China	306 585.30 (234 243.07–404 868.18)	916 180.08 (707 383.77–1 201 380.79)	42.63 (32.40–56.46)	44.92 (34.96–59.42)	0.16 (0.1 to 0.22)	3 195 308.71 (2 518 982.68–4 168 289.54)	10 775 720.75 (8 531 627.04–14 014 035.77)	457.72 (358.93–594.96))	524.00 (418.15–681.23)	0.48 (0.38 to 0.58)
Female	153 612.81 (116 452.58–204 458.07)	464 203.28 (351 459.04–620 286.85)	41.74 (31.35–55.99)	43.28 (32.90–57.37)	−0.05 (−0.12 to 0.03)	1 567 409.86 (1 228 170.49–2 055 586.65)	5 148 954.15 (4 069 341.02–6 741 869.48)	429.29 (334.63–563.04)	473.40 (373.44–613.68)	0.17 (0.06 to 0.27)
Male	152 972.48 (117 440.82–199 656.08)	451 976.80 (348 035.57–591 929.52)	42.00 (32.04–55.80)	45.23 (35.38–59.31)	0.39 (0.34 to 0.45)	1 627 898.85 (1 274 804.73–2 125 145.28)	5 626 766.59 (4 446 323.42–72 895	487.47 (385.37–637.07)	574.50 (456.90–745.56)	0.77 (0.65 to 0.88)

	Number of DALYs cases		ASDAR			Number of deaths cases		ASDR		
	1990	2021	1990	2021	EAPC (95%UI)	1990	2021	1990	2021	EAPC (95%UI)
China	508 610.26 (395 853.05–638 618.04)	1 653 116.54 (1 303 681.18–2 056 459.47)	93.28 (75.14–115.50)	89.76 (72.13–109.67)	−0.21 (−0.31 to −0.11)	16 448.97 (13 239.72–20 520.66)	64 728.22 (51 764.98–77 729.27)	4.93 (3.88–6.17)	4.33 (3.43–5.23)	−0.6 (−0.78 to −0.43)
Female	286 723.05 (226 414.97–359 764.59)	900 010.10 (695 764.56–1 116 491.57)	97.72 (78.59–120.87)	87.68 (67.72–108.49)	−0.55 (−0.67 to −0.44)	11 242.15 (8604.47–14 407.99)	42 939.03 (32 263.25–54 477.57)	5.54 (4.27–7.06)	4.58 (3.45–5.83)	−0.85 (−1.03 to −0.67)
Male	221 887.21 (162 650.95–288 078.11)	753 106.44 (569 016.60–959 037.31)	81.96 (62.57–101.66)	89.62 (70.01–111.29)	0.4 (0.3 to 0.49)	5206.83 (3707.76–6632.37)	21 789.19 (16 730.16–28 054.00)	3.56 (2.57–4.35)	3.81 (2.99–4.80)	0.22 (0.08 to 0.37)

Data in parentheses denote the 95% uncertainty interval.

AF/AFL, atrial fibrillation and atrial flutter; ASDAR, age‐standardized DALYs rate; ASDR, age‐standardized deaths rate; ASIR, age‐standardized incidence rate; ASPR, age‐standardized prevalence rate; DALYs, disability‐adjusted life years; UI, uncertainty interval.

### Sex‐ and age‐specific trends in AF/AFL burden in China

**Figure 2:**
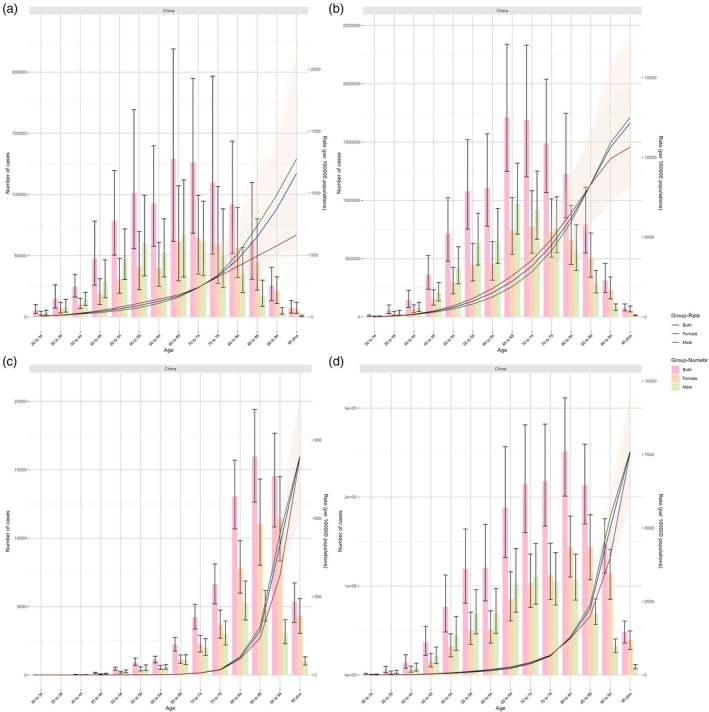
Age‐Patterned temporal trends in numbers and age‐standardized rates of (a) incident cases of, (b) prevalence cases of, (c) disability‐adjusted life years cases of, and (d) deaths from AF/AFL at the mainland China level in 2021.

Figure 2 illustrates age‐ and sex‐stratified trends in AF/AFL incidence, prevalence, mortality, DALYs, and age‐standardized rates in 2021. In China, the highest number of incident (120 000) and prevalent (1.71 million) cases occurred in the 65–69‐year age group. Mortality peaked in the 85–89‐year age group (15 000 deaths), while DALYs were highest in the 80–84 year group (250 000 DALYs).

Before age 65–69, males had higher incidence rates than females, but female incidence surpassed that of males thereafter. Similarly, male prevalence exceeded female prevalence until age 75–79, after which females predominated. Female mortality rates exceeded those of males across all age groups. Male DALYs were higher than female DALYs until age 70–74, after which females showed higher DALYs (Fig. 2). Both sexes exhibited age‐dependent increases in standardized incidence, prevalence, mortality, and DALY rates (Fig. 2). Current trends by age group are detailed in Figure S1.

### Risk factor analysis for AF/AFL‐related mortality and DALYs in China

Among all risk factors in GBD 2021, elevated systolic blood pressure contributed most to AF/AFL‐related mortality (30.3%, 95% UI: 10.6%–40.6%), followed by high sodium intake (4.8%, 95% UI: 0.6%–12.7%), smoking (4.2%, 95% UI: 2.3%–6.1%), high BMI (3.7%, 95% UI: 1.5%–6.5%), and lead exposure (3.5%, 95% UI: −0.5%–8.9%) (Table 2). For DALYs, elevated systolic blood pressure remained the leading contributor (29.8%, 95% UI: 10.2%–47.5%), followed by smoking (6.9%, 95% UI: 4.1%–9.5%), high sodium intake (6.6%, 95% UI: 1.3%–15.9%), high BMI (4.5%, 95% UI: 1.9%–7.9%), and lead exposure (3.4%, 95% UI: −0.5%–8.8%) (Table 2).

**Table 2:** Burden contributions of major risk factors to age‐standardized deaths and DALYs of atrial fibrillation/atrial flutter in 2021

	DALYs (95% UI), in thousands			Age‐standardized DALY rate per 100 000 population (95% UI)		
	Total	Male	Female	Total	Male	Female
All risk factors	646 399.22 (346 275.90 to 992 273.86)	334 977.75 (191 989.16 to 490 260.63)	311 421.47 (135 325.11 to 509 655.26)	34.26 (18.13 to 52.36)	38.47 (22.08 to 56.59)	30.08 (13.08 to 49.34)
Diet high in sodium	108 957.55 (19 944.97 to 260 666.09)	61 545.26 (13 158.18 to 138 446.92)	47 412.28 (7519.61 to 122 368.53)	5.43 (0.95 to 13.42)	6.55 (1.29 to 15.46)	4.44 (0.67 to 11.71)
Dietary risks	108 957.55 (19 944.97 to 260 666.09)	61 545.26 (13 158.18 to 138 446.92)	47 412.28 (7519.61 to 122 368.53)	5.43 (0.95 to 13.42)	6.55 (1.29 to 15.46)	4.44 (0.67 to 11.71)
Environmental/occupational risks	56 945.30 (−7718.89 to 146 211.01)	29 630.79 (−3789.18 to 76 055.40)	27 314.51 (−3935.61 to 70 181.00)	3.09 (−0.43 to 8.01)	3.60 (−0.47 to 9.15)	2.67 (−0.39 to 6.80)
High body mass index	75 251.71 (29 279.94 to 129 907.15)	25 703.62 (9567.47 to 44 812.36)	49 548.08 (19 278.13 to 88 250.03)	3.84 (1.51 to 6.57)	2.83 (1.08 to 4.89)	4.61 (1.80 to 8.16)
High systolic blood pressure	493 592.74 (166 429.49 to 854 026.61)	219 764.63 (70 785.69 to 385 231.35)	273 828.11 (88 986.48 to 478 054.62)	26.71 (9.10 to 46.33)	26.03 (8.36 to 44.72)	26.54 (8.75 to 46.60)
Lead exposure	56 945.30 (−7718.89 to 146 211.01)	29 630.79 (−3789.18 to 76 055.40)	27 314.51 (−3935.61 to 70 181.00)	3.09 (−0.43 to 8.01)	3.60 (−0.47 to 9.15)	2.67 (−0.39 to 6.80)
Smoking	113 500.61 (63 199.64 to 171 028.24)	99 059.39 (55 675.79 to 149 468.23)	14 441.22 (7694.24 to 23 622.65)	5.51 (3.05 to 8.26)	10.46 (5.97 to 15.89)	1.38 (0.72 to 2.27)

	Deaths (95% UI), in thousands			Age‐standardized death rate per 100 000 population (95% UI)		
	Total	Male	Female	Total	Male	Female
All risk factors	23 548.57 (12 076.96 to 36 921.39)	9000.06 (5119.21 to 13 948.98)	14 548.51 (6057.68 to 24 490.27)	1.54 (0.76 to 2.46)	1.51 (0.87 to 2.31)	1.54 (0.64 to 2.62)
Diet high in sodium	3128.80 (408.10 to 8541.46)	1375.16 (248.90 to 3651.08)	1753.64 (159.35 to 5000.09)	0.19 (0.02 to 0.54)	0.21 (0.03 to 0.57)	0.04 (0.01 to 0.12)
Dietary risks	3128.80 (408.10 to 8541.46)	1375.16 (248.90 to 3651.08)	1753.64 (159.35 to 5000.09)	0.19 (0.02 to 0.54)	0.21 (0.03 to 0.57)	0.04 (0.01 to 0.12)
Environmental/occupational risks	2270.41 (−369.49 to 5685.05)	912.14 (−132.50 to 2305.11)	1358.27 (−231.67 to 3443.72)	0.15 (−0.02 to 0.38)	0.16 (−0.02 to 0.40)	0.15 (−0.02 to 0.37)
High body mass index	2378.46 (942.02 to 4211.56)	596.19 (229.85 to 1069.47)	1782.28 (692.78 to 3268.13)	0.17 (0.07 to 0.30)	0.08 (0.03 to 0.15)	0.26 (0.10 to 0.47)
High systolic blood pressure	19 586.57 (6868.36 to 32 943.43)	6465.77 (2084.86 to 11 190.37)	13 120.80 (4459.71 to 23 344.05)	1.29 (0.45 to 2.22)	1.10 (0.36 to 1.93)	1.39 (0.48 to 2.49)
Lead exposure	2270.41 (−369.49 to 5685.05)	912.14 (−132.50 to 2305.11)	1358.27 (−231.67 to 3443.72)	0.15 (−0.02 to 0.38)	0.16 (−0.02 to 0.40)	0.15 (−0.02 to 0.37)
Smoking	2681.73 (1472.27 to 4093.20)	2070.51 (1165.76 to 3233.92)	611.22 (280.99 to 1008.86)	0.16 (0.09 to 0.25)	0.32 (0.18 to 0.50)	0.06 (0.03 to 0.11)

DALYs, disability‐adjusted life years; UI, uncertainty interval.

**Figure 3:**
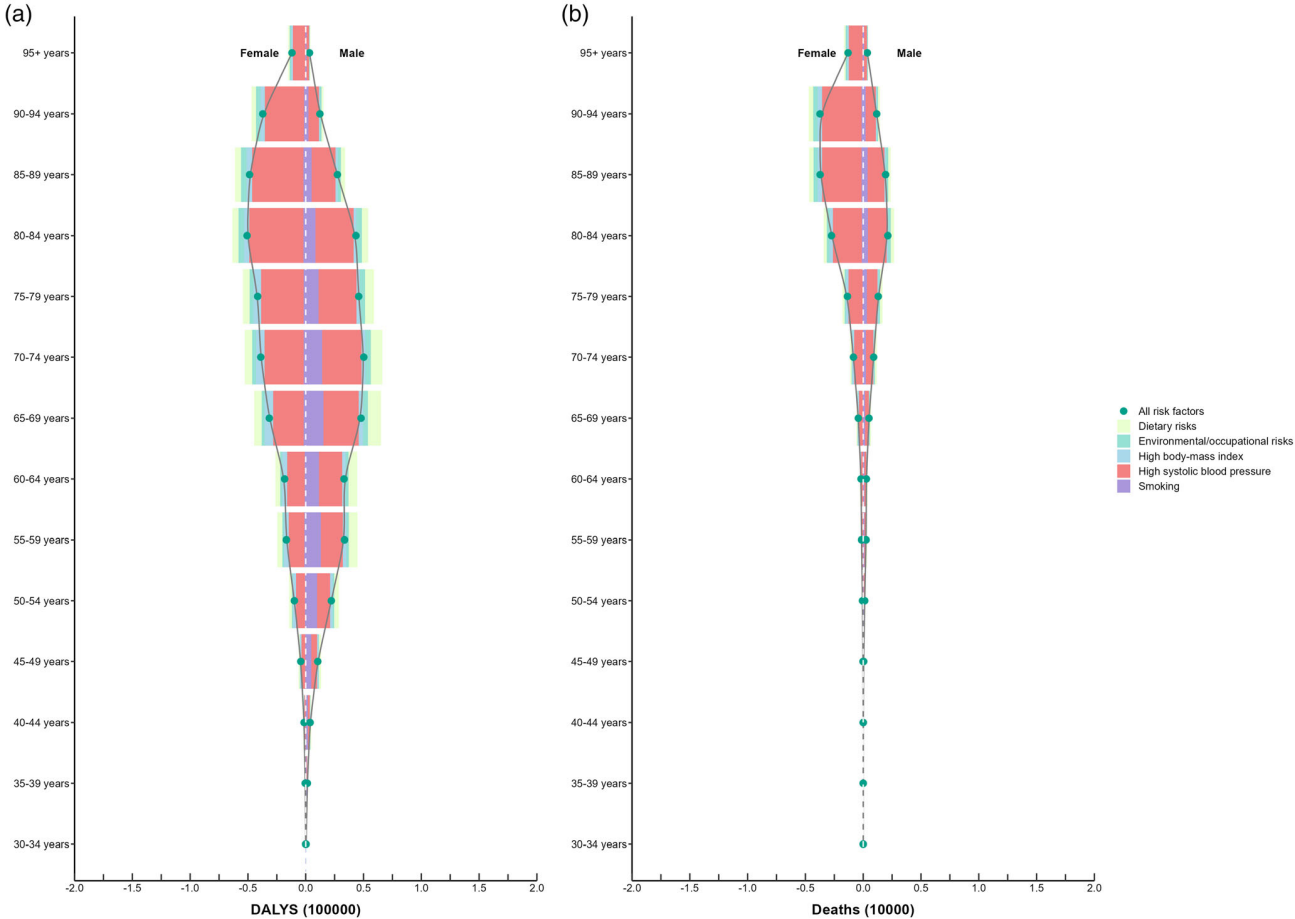
The proportion of the burden of atrial fibrillation attributable to the main risk factors in different age groups. Attributable factors for (a) disability ‐ adjusted life years, (b) deaths.

In females, elevated systolic blood pressure was the predominant risk factor for both mortality and DALYs, with peak contributions in the 80–84‐year age group. Males shared the same leading risk factor (elevated systolic blood pressure). However, for males, smoking and high sodium intake contributed significantly to mortality (peak at 80–84 years) and DALYs (peak at 70–74 years) compared with females (Fig. 3).

### Projection analysis of AF/AFL disease burden in China through 2045

The Norpred model was employed to predict the future disease burden of AF/AFL in China from 2022 to 2045. Our results indicate that over the next 33 years, the burden of AF/AFL in China will experience significant upward trends. By 2045, the total number of incident AF/AFL cases in China is projected to reach 1 931 087, and deaths attributable to AF/AFL are expected to total 219 658 (Tables S1 and S2). From a sex perspective, by 2045, the incident cases of AF/AFL among males and females in China are projected to be 862 546 and 1 068 541, and death counts are projected to be 66 050 for males and 153 608 for females, with age‐standardized mortality rates of 7.28 and 10.09 (Tables S1 and S2). In terms of age distribution, the majority of incident and prevalent cases in China by 2045 are expected to cluster in the 75–79 years age group. The highest mortality is projected for the 90–94 years age group. In males, age‐specific trends mirrored the overall population, whereas females exhibited higher incident and prevalent cases in the 75–84 years age group, with the highest mortality in the 90–94 years group and DALYs concentrated in the 80–94 years group (Figs S2 and S4, Tables S3 and S4).

**Figure 4:**
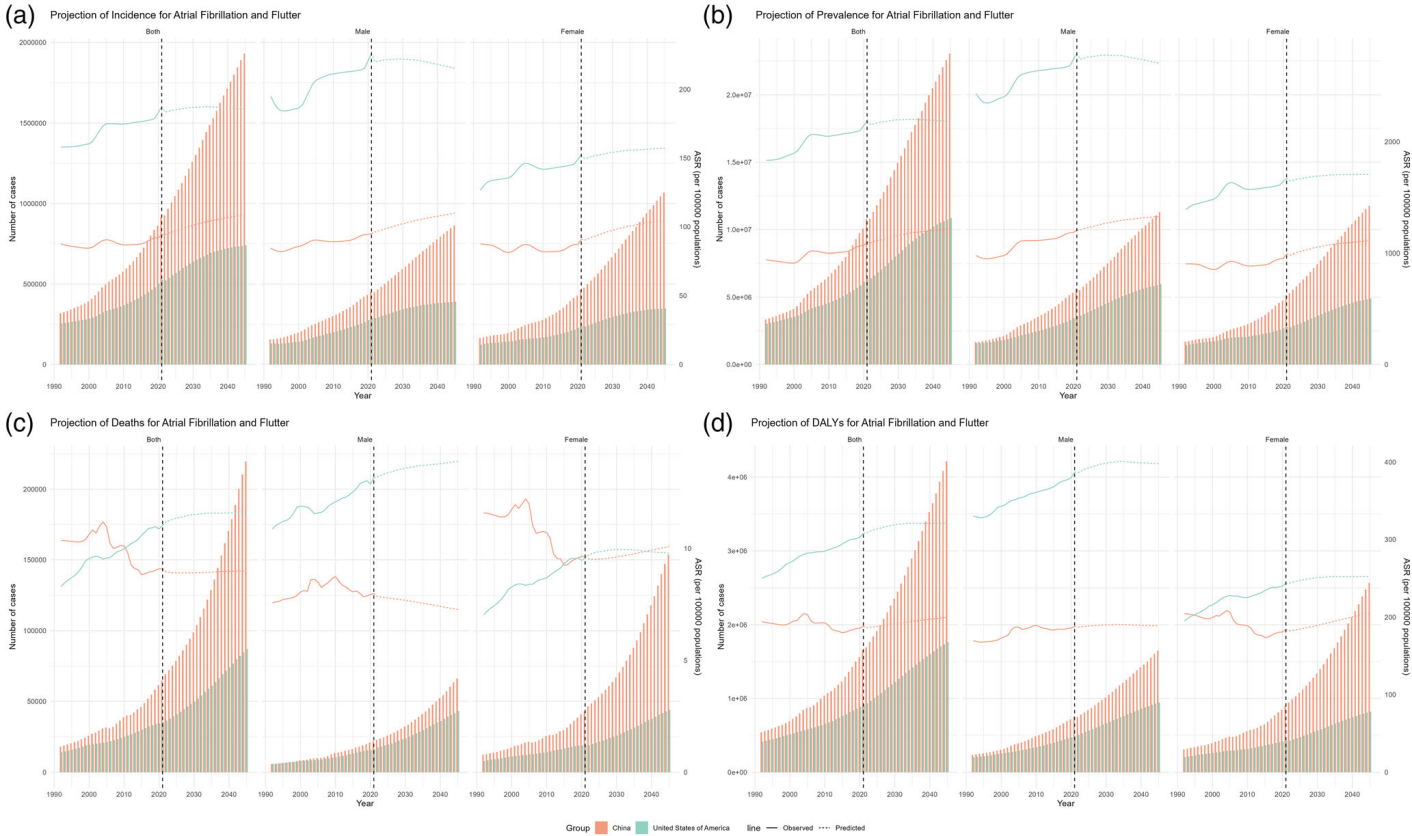
Prediction of future burden of atrial fibrillation/atrial flutter numbers and age‐standardized rates of (a) incident cases, (b) prevalence cases, (c) disability‐adjusted life years cases, and (d) deaths in different sex groups in mainland China and the United States.

A comparative analysis with the United States highlighted the following three key findings. (i) Similar to China, the United States is projected to experience rising trends in AF/AFL incident cases, age‐standardized incidence, prevalent cases, mortality, and DALYs over the next 33 years. (ii) China demonstrates higher crude incidence, prevalence, and DALYs rates compared with the United States; however, its overall age‐standardized rates remain lower than those of the United States. (iii) By 2045, incident cases in China are projected to be higher among females than among males. In contrast, the opposite trend is observed in the United States, where males are expected to dominate incident cases relative to females (Fig. 4, Tables S1 and S2).

## Discussion

### Key findings of this study

Based on the GBD 2021 data, this study systematically analyzed trends in the disease burden of AF/AFL in China from 1990 to 2021 and projected future trajectories. Our findings were as follows. (i) Current trends (1990–2021): China experienced significant shifts in the AF/AFL burden. The age‐standardized incidence and prevalence rates increased by 5.4% and 14.5%, respectively, while age‐standardized mortality and DALY rates declined by 12.2% and 3.8%. (ii) Future projections: By 2045, the prevalence of AF/AFL in China is expected to exceed 23 million, with incident cases rising to approximately 1.93 million. (iii) Sex‐specific disparities: By 2045, females are expected to show a significant decrease in age‐standardized mortality (by 17.3%) and in DALY rates (by 10.3%), whereas males will exhibit adverse trends, with a ~7.0% increase in mortality and ~9.3% rise in DALY rates. (iv) Core risk factors in the Chinese population: Elevated systolic blood pressure, smoking, high‐sodium diet, and elevated BMI were identified as predominant contributors, with hypertension exhibiting the highest population‐attributable fraction (PAF), particularly among older adults.

### Epidemiological characteristics of AF/AFL in China, 1990–2021

Data from the GBD Study 2021 revealed approximately 52.55 million global AF/AFL cases in 2021, with China accounting for 8.5% (4.48 million incident cases, 340 000 deaths, and 8.36 million DALYs).^2^ This study demonstrates that China's age‐standardized incidence and prevalence rates of AF/AFL increased by 5.4% and 14.5%, respectively, from 1990 to 2021, aligning with global trends reported in GBD 2021.^2^ Notably, China achieved a 12.2% reduction in age‐standardized mortality and a 3.8% decline in DALYs during this period, outperforming most developing nations. These improvements correlate with economic development, enhanced lifestyle quality, and advancements in national cardiovascular disease prevention policies, including early screening, diagnosis, and standardized management of chronic conditions such as AF/AFL.^17^, ^18^, ^19^

Clinically, innovations in cardiovascular interventions (e.g. refined ablation techniques), novel anticoagulants, and improved treatment precision (20–35% enhancement, according to the China Cardiovascular Health Report 2021) have propelled progress.^20^ Public health policies, such as the “Comprehensive Chronic Disease Prevention and Control Demonstration Zones” established in 2017, raised high‐risk population screening coverage to 82.6% through a tiered prevention system. Following implementation of the “early screening–early diagnosis–standardized management” model for AF/AFL, rural stroke incidence decreased by 18.3%, and the treatment rate of anticoagulant drugs gradually increased.^21^, ^22^

The WHO 2023 Global Health Assessment highlighted China's integrated approach combining medical assistance and critical illness insurance. It targeted health poverty alleviation, which elevated standardized anticoagulation therapy rates among low‐income rural AF/AFL patients from 29.4% (2015) to 67.1% (2022), offering an innovative chronic disease management model for developing nations.^23^, ^24^

### Projection of disease burden and response strategies for AF/AFL in China by 2045

This study utilized longitudinal data from the GBD database spanning 1990–2021, and employed the Norpred algorithm within the Bayesian APC (BAPC) model for predictive modeling. Using Markov chain Monte Carlo (MCMC) simulations and considering covariates such as demographic shifts, risk factor exposure, and healthcare accessibility, the model forecasted a “four‐dimensional escalation” in China's AF/AFL disease burden by 2045. Specifically, the number of AF/AFL cases is projected to increase approximately 3.7‐fold from the 2021 baseline, reaching 23.17 million (95% UI: 21.84–24.06 million) in 2045. Annual incidence is expected to surpass 1.93 million cases, marking a 121% increase from 2021. Deaths are anticipated to exceed 220 000, with the age‐standardized mortality rate rising to 8.7 per 100 000. DALYs are projected to reach 4.89 million, with the 80–94 age group accounting for 62.3% of the disease burden.

These alarming trends are driven primarily by the following two key factors. (i) Accelerated demographic transition: According to predictions from China's National Bureau of Statistics, the proportion of the population aged 60 and above will increase from 20.4% in 2023 to 36.8% by 2045, characterizing a “super‐aged society.” The AF incidence rate among the 75–79 age group is 8.9%, 12.6 times higher than that among the 50–54 age group (GBD 2021 data). (ii) The compounding of metabolic risk factors: Patients with comorbidities such as hypertension and diabetes face a 2.3–3.1‐fold increased risk of AF,^25^ and the prevalence of multiple chronic conditions among the elderly in China is projected to reach 79.4% by 2045. The predictive model suggests that without effective interventions, AF‐related healthcare expenditures will account for 6.2% of total health expenditures in China by 2045.

To address this challenge, it is imperative to establish a comprehensive prevention and control system encompassing “screening‐diagnosis‐treatment‐rehabilitation‐long‐term management,” tailored to China's aging population. Specific strategies include: (i) establishing an age‐stratified prevention and control system; (ii) innovating anticoagulation therapy management models; (iii) advancing digital health solutions; and (iv) enhancing social security policies. In implementing these strategies, special attention must be paid to sex disparities (17% increased stroke risk in women) and regional disparities (39% higher mortality in western regions compared with in eastern regions), allowing the formulation of differentiated and precise prevention and control strategies.

### AF/AFL burden trends in Chinese females in the future

This study revealed significant sex disparities in the disease burden of AF/AFL. Among females, the age‐standardized mortality rate decreased from 5.54 per 100 000 in 1990 to 4.58 per 100 000 in 2021 (a 17.3% reduction), while the age‐standardized DALYs declined from 97.72 per 100 000 to 87.68 per 100 000 (a 10.3% reduction). In contrast, males exhibited an increase in age‐standardized mortality from 3.56 per 100 000 to 3.81 per 100 000 (a 7.0% rise), and age‐standardized DALYs increased from 81.96 per 100 000 to 89.62 per 100 000 (a 9.3% rise). Notably, after age standardization, the DALYs rate for males surpassed that of females by 2021.

The observed sex disparities, along with the lower age‐standardized incidence and prevalence rates among females, may be driven by the following multifactorial influences. (i) Differences in risk factor exposure, such as smoking and harmful alcohol consumption. (ii) Health management and disease awareness: Females are more likely to participate in regular health check‐ups than males. (iii) Biological mechanisms: The incidence of AF/AFL is relatively lower in premenopausal women.^26^, ^27^, ^28^, ^29^ However, it significantly increases in postmenopausal women, particularly after the age of 50, which is primarily attributed to changes in estrogen levels.^30^, ^31^ (iv) Demographic and lifespan differences: The male population in China is projected to outnumber the female population, particularly in younger age groups, while females have a longer life expectancy. The absolute number of AF cases and lifetime risk (approximately 23%) are similar between sexes.^32^ (v) Comorbidities and aging: Female AF patients tend to have more comorbidities and a later onset of the disease.^33^, ^34^, ^35^

### Attributable risk factor analysis and key prevention strategies

This study identified that the primary risk factors for AF/AFL in China include high systolic blood pressure, smoking, high‐sodium diet, and elevated BMI. Compared with previous research, the 2021 GBD study highlighted that high systolic blood pressure accounted for the highest proportion of risk factors contributing to AF/AFL‐related mortality, followed by high BMI, alcohol consumption, high‐sodium diet, smoking, and lead exposure.^2^ In both Chinese males and females, high systolic blood pressure emerged as the dominant risk factor, contributing most significantly to DALYs and mortality, particularly among individuals aged 85–89 years. To address high systolic blood pressure as a primary risk factor, lifestyle modifications are recommended as first‐line preventive strategies, including weight reduction, healthy dietary habits, reduced sodium intake, increased potassium intake, and enhanced physical activity.^36^

In addition to lifestyle modification, the effectiveness and adherence of antihypertensive medications play a crucial role in the management of AF and explain the divergent trends between morbidity and mortality. Evidence suggests that the European Society of Cardiology (ESC)/European Society of Hypertension (ESH) hypertension guidelines recommend initial dual therapy consisting of a beta‐blocker combined with a Renin–Angiotensin System (RAS) blocker or a dihydropyridine calcium channel blocker (CCB).^37^ For patients with preserved ejection fraction who are intolerant of beta‐blockers, non‐dihydropyridine CCBs such as verapamil or diltiazem may be appropriate alternatives.^37^ In addition, valsartan has been shown to reduce the risk of new‐onset AF in high‐risk individuals compared with amlodipine, and the losartan intervention for end point (LIFE) study found losartan to be superior to atenolol in reducing both new‐onset AF and stroke risk.^38^ Furthermore, antihypertensive drugs that target proteins to lower systolic blood pressure are genetically likely to prevent AF,^39^ and these findings highlight the importance of selecting appropriate antihypertensive drugs and ensuring medication adherence as part of a comprehensive AF management strategy.

Among males, smoking and high‐sodium diets also play critical roles, with their impact on disease burden markedly higher than in females. A large US prospective cohort study demonstrated that smoking not only elevates AF incidence but also increases all‐cause mortality and cardiovascular adverse events.^40^ In males aged 80–84 years, elevated mortality and DALYs underscore the pronounced effects of high systolic blood pressure and smoking during this life stage. Consequently, males face greater challenges in mitigating unhealthy lifestyles, particularly in tobacco control and dietary improvements. Meanwhile, hypertension and obesity in females warrant heightened attention. Prior studies have established obesity as an independent predictor of AF/AFL,^41^ closely linked to diabetes and hypertension.^42^ Thus, obese adults exhibit the highest risk of AF/AFL. To reduce the percentage contribution of high BMI and systolic blood pressure to age‐standardized AF/AFL mortality in females, nationwide initiatives promoting physical activity, dietary optimization, and restriction of high‐salt and high‐fat foods are recommended.

## Conclusion

This study outlines the increasing burden of AF/AFL in China (1990–2021) and forecasts a significant public health challenge by 2045, driven by an aging population and comorbidities. Age‐standardized incidence, prevalence, and mortality rates are expected to increase sharply among older people. Targeted interventions, including managing hypertension, reducing smoking, and lowering sodium intake, along with sex‐ and age‐stratified prevention, are essential for sustainable healthcare. Innovations in digital health (e.g., AI‐driven monitoring) and healthcare infrastructure (e.g., tiered AF centers) provide scalable solutions for future prevention and treatment of AF/AFL. Together, future research must consider China‐specific epidemiological nuances to enhance strategies.

## Limitations

While leveraging comprehensive GBD 2021 data, this study has limitations. Regional data gaps in China may reduce representativeness. Moreover, the GBD 2021 database does not recognize the specific classification of complex types of AF/AFL, including paroxysmal, persistent, and permanent AF/AFL, but can only diagnose whether a certain population belongs to AF/AFL. Projections rely on current trends and may not account for future policy shifts. Continuous model updates incorporating real‐world intervention outcomes are warranted.

## Disclosure statement

The authors declare no conflict of interest.

## Author contributions

**Junpeng Xiong:** study design, data analysis, data interpretation, writing. **Xiaoli Wu and Shuwen Chen:** study design, data analysis, data interpretation. **Binyin Wang:** writing, editing, reviewing. **Binquan You:** reviewing. **Ronghui Yu:** study design, reviewing. All authors read and agreed to the final manuscript version.

## Supporting information


**Figure S1.** Temporal trends of age‐standardized rates of (a) incident cases of, (b) prevalence of, (c) disability‐adjusted life years cases of, and (d) deaths from atrial fibrillation/flutter in mainland China from1990 to 2021.
**Figure S2.** Prediction of future burden of atrial fibrillation/atrial flutter in different age groups in mainland China in all population.
**Figure S3.** Prediction of future burden of atrial fibrillation/atrial flutter in different age groups in mainland China for males.
**Figure S4.** Prediction of future burden of atrial fibrillation/atrial flutter in different age groups in mainland China for females.
**Table S1.** Prediction of future burden of atrial fibrillation/atrial flutter in mainland China and the United States for males.
**Table S2.** Prediction of future burden of atrial fibrillation/atrial flutter in mainland China and the United States for females.
**Table S3.** Prediction of future burden of atrial fibrillation/atrial flutter in different age groups in mainland China and the United States for males.
**Table S4.** Prediction of future burden of atrial fibrillation/atrial flutter in different age groups in mainland China and the United States for females.

## Data Availability

The data that supports the findings of this study are available in the supplementary material of this article.

## References

[1] Joglar JA , Chung MK , Armbruster AL *et al*. 2023 ACC/AHA/ACCP/HRS guideline for the diagnosis and Management of Atrial Fibrillation: a report of the American College of Cardiology/American Heart Association joint committee on clinical practice guidelines. Circulation 2024; 149: e167. 10.1161/CIR.0000000000001207. 38033089 PMC11095842

[2] Cheng S , He J , Han Y *et al*. Global burden of atrial fibrillation/atrial flutter and its attributable risk factors from 1990 to 2021. Europace 2024; 26: euae195. 10.1093/europace/euae195. 38984719 PMC11287210

[3] Li X , Liu Z , Jiang X *et al*. Global, regional, and national burdens of atrial fibrillation/flutter from 1990 to 2019: an age‐period‐cohort analysis using the global burden of disease 2019 study. J Glob Health 2023; 13: 04154. 10.7189/jogh.13.04154. 37988383 PMC10662782

[4] Naccarelli GV , Varker H , Lin J , Schulman KL . Increasing prevalence of atrial fibrillation and flutter in the United States. Am J Cardiol 2009; 104: 1534–1539. 19932788 10.1016/j.amjcard.2009.07.022

[5] Krijthe BP , Kunst A , Benjamin EJ *et al*. Projections on the number of individuals with atrial fibrillation in the European Union, from 2000 to 2060. Eur Heart J 2013; 34: 2746–2751. 23900699 10.1093/eurheartj/eht280 PMC3858024

[6] Lancet . Population ageing in China: crisis or opportunity? Lancet 2022; 400: 1821. 10.1016/S0140-6736(22)02410-2. 36436518

[7] GBD 2021 Diseases and Injuries Collaborators . Global incidence, prevalence, years lived with disability (YLDs), disability‐adjusted life‐years (DALYs), and healthy life expectancy (HALE) for 371 diseases and injuries in 204 countries and territories and 811 subnational locations, 1990–2021: a systematic analysis for the global burden of disease study 2021. Lancet 2024; 403: 2133–2161. 10.1016/S0140-6736(24)00757-8. 38642570 PMC11122111

[8] GBD 2021 Risk Factors Collaborators . Global burden and strength of evidence for 88 risk factors in 204 countries and 811 subnational locations, 1990–2021: a systematic analysis for the global burden of disease study 2021. Lancet 2024; 403: 2162–2203. 10.1016/S0140-6736(24)00933-4. 38762324 PMC11120204

[9] Global age‐sex‐specific fertility, mortality, healthy life expectancy (HALE), and population estimates in 204 countries and territories . 1950‐2019: a comprehensive demographic analysis for the global burden of disease study 2019. Lancet 2020; 396: 1160–1203. 10.1016/S0140-6736(20)30977-6. 33069325 PMC7566045

[10] Tian N , Zheng JX , Guo ZY , Li LH , Xia S , Lv S . Dengue incidence trends and its burden in major endemic regions from 1990 to 2019. Trop Med Infect Dis 2022; 7: 180. 10.3390/tropicalmed7080180. 36006272 PMC9416661

[11] Zheng X , Guan Q , Lin X . Changing trends of the disease burden of non‐rheumatic valvular heart disease in China from 1990 to 2019 and its predictions: findings from global burden of disease study. Front Cardiovasc Med 2023; 9: 912661. 10.3389/fcvm.2022.912661. 36741848 PMC9897059

[12] Dai H , Zhang Q , Much AA *et al*. Global, regional, and national prevalence, incidence, mortality, and risk factors for atrial fibrillation, 1990‐2017: results from the global burden of disease study 2017. Eur Heart J Qual Care Clin Outcomes 2021; 7: 574–582. 10.1093/ehjqcco/qcaa061. 32735316 PMC8557444

[13] Roth GA , Mensah GA , Johnson CO *et al*. Global burden of cardiovascular diseases and risk factors, 1990–2019: update from the GBD 2019 study. J Am Coll Cardiol 2020; 76: 2982–3021. 10.1016/j.jacc.2020.11.010. 33309175 PMC7755038

[14] GBD 2019 Risk Factors Collaborators . Global burden of 87 risk factors in 204 countries and territories, 1990–2019: a systematic analysis for the global burden of disease study 2019. Lancet 2020; 396: 1223–1249. 10.1016/S0140-6736(20)30752-2. 33069327 PMC7566194

[15] Liu Z , Jiang Y , Yuan H *et al*. The trends in incidence of primary liver cancer caused by specific etiologies: results from the global burden of disease study 2016 and implications for liver cancer prevention. J Hepatol 2019; 70: 674–683. 10.1016/j.jhep.2018.12.001. 30543829

[16] GBD . Global fertility， mortality，migration，and population forecasts 2017—2100[EB/OL], 2022. https://ghdx.healthdata.org/record/ihme-data/global-populationforecasts-2017-2100.

[17] Guo Y , Wang H , Zhang H *et al*. Mobile Photoplethysmographic technology to detect atrial fibrillation. J Am Coll Cardiol 2019; 74: 2365–2375. 10.1016/j.jacc.2019.08.019. 31487545

[18] Guo Y , Lane DA , Wang L *et al*. Mobile health technology to improve Care for Patients with Atrial Fibrillation. J Am Coll Cardiol 2020; 75: 1523–1534. 10.1016/j.jacc.2020.01.052. 32241367

[19] Wei Y , Zhou G , Wu X *et al*. Latest incidence and electrocardiographic predictors of atrial fibrillation: a prospective study from China. Chin Med J 2023; 136: 313–321. 10.1097/CM9.0000000000002340. 36989484 PMC10106138

[20] National Center for Cardiovascular Diseases . Annual Report on Cardiovascular Health and Diseases in China (2021). Beijing: China Science Publishing & Media Ltd, 2022.

[21] Chen M , Li C , Liao P *et al*. Epidemiology, management, and outcomes of atrial fibrillation among 30 million citizens in Shanghai, China from 2015 to 2020: a medical insurance database study. Lancet Reg Health West Pac 2022; 23: 100470. 10.1016/j.lanwpc.2022.100470. 35542895 PMC9079299

[22] Zhao QY , Shi SB , Huang H *et al*. Contemporary characteristics, management, and outcomes of patients hospitalized for atrial fibrillation in China: results from the real‐world study of Chinese atrial fibrillation registry. Chin Med J 2020; 133: 2883–2884. 10.1097/CM9.0000000000001151. 33273341 PMC10631585

[23] Wang Z , Ma L , Liu M , Fan J , Hu S , Writing Committee of the Report on Cardiovascular Health and Diseases in China . Summary of the 2022 report on cardiovascular health and diseases in China. Chin Med J 2023; 136: 2899–2908. 10.1097/CM9.0000000000002927. 38018129 PMC10752444

[24] Du X , Dong J , Ma C . Is atrial fibrillation a preventable disease? J Am Coll Cardiol 2017; 69: 1968–1982. 10.1016/j.jacc.2017.02.020. 28408027

[25] Rao C , Bongiovanni T , Li X *et al*. The China patient‐Centred evaluative assessment of cardiac events (China PEACE)‐prospective study of 3‐vessel disease: rationale and design. BMJ Open 2016; 6: e009743. 10.1136/bmjopen-2015-009743. PMC4762131 26880670

[26] Stewart S , Hart CL , Hole DJ , McMurray JJ . A population‐based study of the long‐term risks associated with atrial fibrillation: 20‐year follow‐up of the Renfrew/Paisley study. Am J Med 2002; 113: 359–364. 10.1016/s0002-9343(02)01236-6. 12401529

[27] Westerman S , Wenger N . Gender differences in atrial fibrillation: a review of epidemiology, management, and outcomes. Curr Cardiol Rev 2019; 15: 136–144. 10.2174/1573403X15666181205110624. 30516110 PMC6520576

[28] Tian XT , Xu YJ , Yang YQ . Gender differences in arrhythmias: focused on atrial fibrillation. J Cardiovasc Transl Res 2020; 13: 85–96. 10.1007/s12265-019-09918-w. 31637585

[29] GBD 2017 Causes of Death Collaborators . Global, regional, and national age‐sex‐specific mortality for 282 causes of death in 195 countries and territories, 1980–2017: a systematic analysis for the global burden of disease study 2017. Lancet 2018; 392: 1736–1788. 10.1016/S0140-6736(18)32203-7. 30496103 PMC6227606

[30] Gautam S , Shankar N , Tandon OP , Goel N . Comparison of cardiac autonomic functions among postmenopausal women with and without hormone replacement therapy, and premenopausal women. Indian J Physiol Pharmacol 2011; 55: 297–303. 23362720

[31] Ko D , Rahman F , Schnabel RB , Yin X , Benjamin EJ , Christophersen IE . Atrial fibrillation in women: epidemiology, pathophysiology, presentation, and prognosis. Nat Rev Cardiol 2016; 13: 321–332. 10.1038/nrcardio.2016.45. 27053455 PMC5579870

[32] Lloyd‐Jones DM , Wang TJ , Leip EP *et al*. Lifetime risk for development of atrial fibrillation: the Framingham heart study. Circulation 2004; 110: 1042–1046. 10.1161/01.CIR.0000140263.20897.42. 15313941

[33] Magnussen C , Niiranen TJ , Ojeda FM *et al*. Sex differences and similarities in atrial fibrillation epidemiology, risk factors, and mortality in community cohorts: results from the BiomarCaRE consortium (biomarker for cardiovascular risk assessment in Europe). Circulation 2017; 136: 1588–1597. 10.1161/CIRCULATIONAHA.117.028981. 29038167 PMC5657474

[34] Schnabel RB , Yin X , Gona P *et al*. 50 year trends in atrial fibrillation prevalence, incidence, risk factors, and mortality in the Framingham heart study: a cohort study. Lancet 2015; 386: 154–162. 10.1016/S0140-6736(14)61774-8. 25960110 PMC4553037

[35] Leventopoulos G , Koros R , Travlos C *et al*. Mechanisms of atrial fibrillation: how our knowledge affects clinical practice. Life (Basel) 2023; 13: 1260. 10.3390/life13061260. 37374043 PMC10303005

[36] Whelton PK , Carey RM , Aronow WS *et al*. 2017 ACC/AHA/AAPA/ABC/ACPM/AGS/APhA/ASH/ASPC/NMA/PCNA guideline for the prevention, detection, evaluation, and management of high blood pressure in adults: a report of the American College of Cardiology/American Heart Association task force on clinical practice guidelines. Hypertension 2018; 71: e13–e115. 10.1161/HYP.0000000000000065. 29133356

[37] Williams B , Mancia G , Spiering W *et al*. 2018 ESC/ESH guidelines for the management of arterial hypertension. Eur Heart J 2019; 40: 475. 10.1093/eurheartj/ehy686. 31005980

[38] Wachtell K , Lehto M , Gerdts E *et al*. Angiotensin II receptor blockade reduces new‐onset atrial fibrillation and subsequent stroke compared to atenolol: the losartan intervention for end point reduction in hypertension (LIFE) study. J Am Coll Cardiol 2005; 45: 712–719. 10.1016/j.jacc.2004.10.068. 15734615

[39] Geurts S , Tilly MJ , Lu Z *et al*. Antihypertensive drugs for the prevention of atrial fibrillation: a drug target mendelian randomization study. Hypertension 2024; 81: 1766–1775. 10.1161/HYPERTENSIONAHA.123.21858. 39018378 PMC11251507

[40] Tasdighi E , Yao Z , Jha KK *et al*. Cigar, pipe, and smokeless tobacco use and cardiovascular outcomes from cross cohort collaboration. JAMA Netw Open 2025; 8: e2453987. 10.1001/jamanetworkopen.2024.53987. 39804647 PMC11731180

[41] Karasoy D , Bo Jensen T , Hansen ML *et al*. Obesity is a risk factor for atrial fibrillation among fertile young women: a nationwide cohort study. Europace 2013; 15: 781–786. 10.1093/europace/eus422. 23284141

[42] Grundvold I , Bodegard J , Nilsson PM *et al*. Body weight and risk of atrial fibrillation in 7,169 patients with newly diagnosed type 2 diabetes; an observational study. Cardiovasc Diabetol 2015; 14: 5. 10.1186/s12933-014-0170-3. 25589001 PMC4299152

