# Supplementary material for: Aging China's cardiac challenge: Projected growth of atrial arrhythmia cases and healthcare preparedness through 2045 — A global burden disease analysis
Source: Geriatr Gerontol Int. 2025 Jun 25;25(8):1047–57. doi: 10.1111/ggi.70089 (PMC12336437; doi:10.1111/ggi.70089)
Supplement: Supplementary file 1 — Figure S1. Temporal trends of age‐standardized rates of (a) incident cases of, (b) prevalence of, (c) disability‐adjusted life years cases of, and (d) deaths from atrial fibrillation/flutter in mainland China from1990 to 2021. Figure S2. Prediction of future burden of atrial fibrillation/atrial flutter in different age groups in mainland China in all population. Figure S3. Prediction of future burden of atrial fibrillation/atrial flutter in different age groups in mainland China for males. Figure S4. Prediction of future burden of atrial fibrillation/atrial flutter in different age groups in mainland China for females. Table S1. Prediction of future burden of atrial fibrillation/atrial flutter in mainland China and the United States for males. Table S2. Prediction of future burden of atrial fibrillation/atrial flutter in mainland China and the United States for females. Table S3. Prediction of future burden of atrial fibrillation/atrial flutter in different age groups in mainland China and the United States for males. Table S4. Prediction of future burden of atrial fibrillation/atrial flutter in different age groups in mainland China and the United States for females. [file GGI-25-1047-s001.docx]

**Supplemental Materials**

**Supplemental Table1(TableS1)**

**Supplemental Table2(TableS2)**

**Supplemental Table3(TableS3)**

**Supplemental Table4(TableS4)**

**Supplemental Figure1(FigureS1)**

**Supplemental Figure2(FigureS2)**

**Supplemental Figure3(FigureS3)**

**Supplemental Figure4(FigureS4)**

**Supplymental Table1** Prediction of future burden of atrial fibrillation/atrial flutter in mainland China and the United States in male.

|  | Location | Incidence Number(N) | Incidence ASR (per 100 000) | Prevalence Number(N) | Prevalence ASR (per 100 000) | Deaths Number(N) | Deaths ASR (per 100 000) | DALYs Number(N) | DALYs ASR (per 100 000) |
| --- | --- | --- | --- | --- | --- | --- | --- | --- | --- |
| 2022 | China | 449208 | 96.191852 | 5525490 | 1208.01306 | 22659 | 7.867154 | 748454 | 187.28246 |
| 2023 | China | 466593 | 96.960134 | 5764415 | 1216.919043 | 23640 | 7.843045 | 779510 | 187.768042 |
| 2024 | China | 484010 | 97.728417 | 6002619 | 1225.825026 | 24629 | 7.818935 | 810629 | 188.253624 |
| 2025 | China | 501353 | 98.481086 | 6231954 | 1233.773328 | 25650 | 7.798213 | 841091 | 188.609469 |
| 2026 | China | 518700 | 99.233755 | 6461652 | 1241.72163 | 26759 | 7.77749 | 872313 | 188.965314 |
| 2027 | China | 536808 | 99.986424 | 6706876 | 1249.669931 | 27974 | 7.756767 | 905944 | 189.321159 |
| 2028 | China | 556095 | 100.739093 | 6977923 | 1257.618233 | 29280 | 7.736044 | 942624 | 189.677003 |
| 2029 | China | 574951 | 101.491762 | 7245600 | 1265.566535 | 30572 | 7.715321 | 978552 | 190.032848 |
| 2030 | China | 592889 | 102.13909 | 7495326 | 1271.452535 | 31952 | 7.690423 | 1013337 | 190.144885 |
| 2031 | China | 610425 | 102.786418 | 7740387 | 1277.338534 | 33461 | 7.665525 | 1048630 | 190.256922 |
| 2032 | China | 628793 | 103.433746 | 7998833 | 1283.224534 | 35181 | 7.640627 | 1087315 | 190.368959 |
| 2033 | China | 648839 | 104.081074 | 8283654 | 1289.110534 | 37072 | 7.61573 | 1130326 | 190.480997 |
| 2034 | China | 668239 | 104.728402 | 8560029 | 1294.996534 | 38916 | 7.590832 | 1171778 | 190.593034 |
| 2035 | China | 685883 | 105.227648 | 8808863 | 1298.590477 | 40851 | 7.561943 | 1210776 | 190.430743 |
| 2036 | China | 702759 | 105.726894 | 9046354 | 1302.18442 | 42848 | 7.533054 | 1248857 | 190.268453 |
| 2037 | China | 720178 | 106.22614 | 9293807 | 1305.778364 | 45138 | 7.504166 | 1290400 | 190.106162 |
| 2038 | China | 739590 | 106.725387 | 9569241 | 1309.372307 | 47688 | 7.475277 | 1337118 | 189.943872 |
| 2039 | China | 758688 | 107.224633 | 9833903 | 1312.96625 | 49993 | 7.446388 | 1380300 | 189.781581 |
| 2040 | China | 776389 | 107.653333 | 10075320 | 1315.803373 | 52254 | 7.419198 | 1420757 | 189.620905 |
| 2041 | China | 793202 | 108.082034 | 10301906 | 1318.640495 | 54437 | 7.392007 | 1458987 | 189.46023 |
| 2042 | China | 809887 | 108.510735 | 10533264 | 1321.477617 | 57112 | 7.364817 | 1502277 | 189.299554 |
| 2043 | China | 827922 | 108.939435 | 10791652 | 1324.31474 | 60448 | 7.337627 | 1555108 | 189.138878 |
| 2044 | China | 845680 | 109.368136 | 11042165 | 1327.151862 | 63414 | 7.310436 | 1604638 | 188.978203 |
| 2045 | China | 862546 | 109.796836 | 11275552 | 1329.988984 | 66050 | 7.283246 | 1649334 | 188.817527 |
| 2022 | United States of America | 287144 | 219.739876 | 3624176 | 2739.528525 | 17326 | 13.203338 | 508286 | 386.386449 |
| 2023 | United States of America | 294599 | 220.342724 | 3741202 | 2749.205084 | 18059 | 13.272726 | 526587 | 388.779773 |
| 2024 | United States of America | 302670 | 220.945571 | 3865845 | 2758.881642 | 18817 | 13.342115 | 545921 | 391.173097 |
| 2025 | United States of America | 310073 | 221.14052 | 3978557 | 2762.670937 | 19485 | 13.387883 | 563181 | 392.594263 |
| 2026 | United States of America | 317476 | 221.33547 | 4104951 | 2766.460232 | 20315 | 13.433652 | 582925 | 394.015429 |
| 2027 | United States of America | 324458 | 221.530419 | 4235430 | 2770.249527 | 21223 | 13.479421 | 603600 | 395.436595 |
| 2028 | United States of America | 330996 | 221.725368 | 4359736 | 2774.038823 | 22137 | 13.525189 | 623691 | 396.857761 |
| 2029 | United States of America | 337812 | 221.920317 | 4486443 | 2777.828118 | 23062 | 13.570958 | 644132 | 398.278926 |
| 2030 | United States of America | 344136 | 221.743771 | 4600981 | 2776.456395 | 23899 | 13.598722 | 662393 | 398.814567 |
| 2031 | United States of America | 349710 | 221.567226 | 4722964 | 2775.084673 | 24968 | 13.626487 | 683020 | 399.350207 |
| 2032 | United States of America | 354429 | 221.39068 | 4842991 | 2773.71295 | 26161 | 13.654251 | 704278 | 399.885848 |
| 2033 | United States of America | 359000 | 221.214135 | 4954891 | 2772.341227 | 27340 | 13.682015 | 724684 | 400.421488 |
| 2034 | United States of America | 363747 | 221.037589 | 5069149 | 2770.969504 | 28512 | 13.70978 | 745334 | 400.957129 |
| 2035 | United States of America | 368016 | 220.529946 | 5171657 | 2765.02858 | 29561 | 13.724078 | 763491 | 400.659753 |
| 2036 | United States of America | 371466 | 220.022302 | 5273633 | 2759.087656 | 30910 | 13.738377 | 783682 | 400.362378 |
| 2037 | United States of America | 374099 | 219.514659 | 5367298 | 2753.146732 | 32389 | 13.752676 | 803972 | 400.065003 |
| 2038 | United States of America | 376672 | 219.007016 | 5453217 | 2747.205807 | 33731 | 13.766975 | 822575 | 399.767628 |
| 2039 | United States of America | 379265 | 218.499372 | 5541177 | 2741.264883 | 35026 | 13.781273 | 840968 | 399.470253 |
| 2040 | United States of America | 381696 | 217.940959 | 5625004 | 2734.883638 | 36202 | 13.801012 | 858131 | 399.244758 |
| 2041 | United States of America | 383730 | 217.382547 | 5703475 | 2728.502393 | 37680 | 13.820752 | 877105 | 399.019263 |
| 2042 | United States of America | 385332 | 216.824134 | 5770967 | 2722.121148 | 39207 | 13.840491 | 895425 | 398.793768 |
| 2043 | United States of America | 386851 | 216.265722 | 5832510 | 2715.739903 | 40502 | 13.86023 | 911380 | 398.568273 |
| 2044 | United States of America | 388450 | 215.707309 | 5896374 | 2709.358658 | 41837 | 13.879969 | 927830 | 398.342778 |
| 2045 | United States of America | 390261 | 215.148896 | 5960347 | 2702.977414 | 43115 | 13.899709 | 943836 | 398.117283 |

AF/AFL, atrial fibrillation and atrial flutter; DALYs, disability-adjusted life years; ASIR, age-standardized incidence rate; ASPR, age-standardized Prevalence rate; ASDAR, age-standardized DALYs rate; ASDR, age-standardized Deaths rate;

**Supplymental Table2** Prediction of future burden of atrial fibrillation/atrial flutter in mainland China and the United States in Female.

| Year | Location | Incidence Number(N) | Incidence ASR (per 100 000) | Prevalence Number(N) | Prevalence ASR (per 100 000) | Deaths Number(N) | Deaths ASR (per 100 000) | DALYs Number(N) | DALYs ASR (per 100 000) |
| --- | --- | --- | --- | --- | --- | --- | --- | --- | --- |
| 2022 | China | 477613 | 90.27581 | 5292778 | 983.981117 | 46466 | 9.529979 | 944422 | 182.43756 |
| 2023 | China | 499594 | 91.282116 | 5545122 | 994.253283 | 48517 | 9.519018 | 986157 | 183.052451 |
| 2024 | China | 521740 | 92.288422 | 5799366 | 1004.525448 | 50623 | 9.508058 | 1028512 | 183.667342 |
| 2025 | China | 543865 | 93.165649 | 6048977 | 1013.051622 | 52990 | 9.525147 | 1074402 | 184.633007 |
| 2026 | China | 566492 | 94.042876 | 6303606 | 1021.577797 | 55512 | 9.542237 | 1122146 | 185.598672 |
| 2027 | China | 590434 | 94.920103 | 6574516 | 1030.103971 | 58128 | 9.559326 | 1172505 | 186.564336 |
| 2028 | China | 616238 | 95.79733 | 6869469 | 1038.630145 | 60876 | 9.576415 | 1226373 | 187.530001 |
| 2029 | China | 641913 | 96.674558 | 7163368 | 1047.156319 | 63687 | 9.593504 | 1280441 | 188.495666 |
| 2030 | China | 666723 | 97.357682 | 7442117 | 1053.147916 | 66892 | 9.620229 | 1338236 | 189.564468 |
| 2031 | China | 691713 | 98.040805 | 7721008 | 1059.139512 | 70463 | 9.646954 | 1398970 | 190.633271 |
| 2032 | China | 718396 | 98.723929 | 8016518 | 1065.131109 | 74406 | 9.673679 | 1465025 | 191.702074 |
| 2033 | China | 747695 | 99.407053 | 8339408 | 1071.122705 | 78685 | 9.700404 | 1537241 | 192.770876 |
| 2034 | China | 776327 | 100.090177 | 8653969 | 1077.114302 | 83073 | 9.727129 | 1609080 | 193.839679 |
| 2035 | China | 802839 | 100.549947 | 8940146 | 1080.409669 | 87975 | 9.760119 | 1683774 | 194.93238 |
| 2036 | China | 828577 | 101.009718 | 9216437 | 1083.705036 | 93201 | 9.793109 | 1759570 | 196.025081 |
| 2037 | China | 855592 | 101.469488 | 9506424 | 1087.000402 | 99046 | 9.826099 | 1841889 | 197.117781 |
| 2038 | China | 885235 | 101.929258 | 9826516 | 1090.295769 | 105475 | 9.859089 | 1932455 | 198.210482 |
| 2039 | China | 913146 | 102.389028 | 10129121 | 1093.591136 | 111659 | 9.892079 | 2018613 | 199.303183 |
| 2040 | China | 938948 | 102.811931 | 10408181 | 1096.58768 | 118047 | 9.924425 | 2103753 | 200.37972 |
| 2041 | China | 963026 | 103.234833 | 10667316 | 1099.584224 | 124359 | 9.956771 | 2186002 | 201.456258 |
| 2042 | China | 988358 | 103.657736 | 10934621 | 1102.580768 | 131564 | 9.989117 | 2277023 | 202.532795 |
| 2043 | China | 1017343 | 104.080639 | 11234347 | 1105.577312 | 139825 | 10.021463 | 2382102 | 203.609333 |
| 2044 | China | 1044435 | 104.503542 | 11513317 | 1108.573856 | 147087 | 10.053809 | 2478113 | 204.68587 |
| 2045 | China | 1068541 | 104.926444 | 11761817 | 1111.5704 | 153608 | 10.086155 | 2564622 | 205.762408 |
| 2022 | United States of America | 236570 | 149.57546 | 2775186 | 1648.48937 | 19077 | 9.713786 | 426588 | 243.610186 |
| 2023 | United States of America | 244420 | 150.334791 | 2876746 | 1656.059112 | 19896 | 9.771546 | 442901 | 244.919285 |
| 2024 | United States of America | 252461 | 151.094123 | 2979157 | 1663.628853 | 20722 | 9.829306 | 459370 | 246.228383 |
| 2025 | United States of America | 260020 | 151.632132 | 3074856 | 1668.728085 | 21455 | 9.853797 | 474506 | 247.035509 |
| 2026 | United States of America | 267929 | 152.170141 | 3184001 | 1673.827317 | 22287 | 9.878288 | 491654 | 247.842635 |
| 2027 | United States of America | 275544 | 152.70815 | 3299648 | 1678.926549 | 23222 | 9.902779 | 510141 | 248.649761 |
| 2028 | United States of America | 282642 | 153.246159 | 3411382 | 1684.025781 | 24180 | 9.92727 | 528367 | 249.456887 |
| 2029 | United States of America | 290019 | 153.784168 | 3522527 | 1689.125013 | 25126 | 9.951761 | 546423 | 250.264013 |
| 2030 | United States of America | 297092 | 154.125734 | 3625690 | 1691.847411 | 25954 | 9.951561 | 562844 | 250.632078 |
| 2031 | United States of America | 303474 | 154.467301 | 3737945 | 1694.569808 | 26976 | 9.951361 | 581504 | 251.000144 |
| 2032 | United States of America | 308889 | 154.808867 | 3850351 | 1697.292206 | 28140 | 9.951161 | 601119 | 251.36821 |
| 2033 | United States of America | 314029 | 155.150434 | 3955659 | 1700.014603 | 29309 | 9.950961 | 620062 | 251.736275 |
| 2034 | United States of America | 319438 | 155.492 | 4061287 | 1702.737001 | 30438 | 9.950762 | 638819 | 252.104341 |
| 2035 | United States of America | 324536 | 155.652669 | 4158690 | 1703.218252 | 31434 | 9.932355 | 655759 | 252.067766 |
| 2036 | United States of America | 328618 | 155.813337 | 4256471 | 1703.699502 | 32713 | 9.913949 | 674690 | 252.031191 |
| 2037 | United States of America | 331735 | 155.974006 | 4346872 | 1704.180753 | 34152 | 9.895543 | 694043 | 251.994616 |
| 2038 | United States of America | 334695 | 156.134674 | 4429164 | 1704.662004 | 35475 | 9.877137 | 711824 | 251.958041 |
| 2039 | United States of America | 337664 | 156.295343 | 4511678 | 1705.143254 | 36670 | 9.858731 | 728656 | 251.921467 |
| 2040 | United States of America | 340549 | 156.442674 | 4591066 | 1705.567711 | 37750 | 9.847369 | 744368 | 251.966354 |
| 2041 | United States of America | 342827 | 156.590005 | 4664779 | 1705.992169 | 39129 | 9.836006 | 761783 | 252.011241 |
| 2042 | United States of America | 344511 | 156.737335 | 4727183 | 1706.416626 | 40560 | 9.824644 | 778504 | 252.056129 |
| 2043 | United States of America | 346020 | 156.884666 | 4782683 | 1706.841083 | 41750 | 9.813281 | 792768 | 252.101016 |
| 2044 | United States of America | 347575 | 157.031997 | 4839409 | 1707.265541 | 42888 | 9.801919 | 806778 | 252.145903 |
| 2045 | United States of America | 349313 | 157.179328 | 4895010 | 1707.689998 | 43936 | 9.790557 | 820027 | 252.190791 |

AF/AFL, atrial fibrillation and atrial flutter; DALYs, disability-adjusted life years; ASIR, age-standardized incidence rate; ASPR, age-standardized Prevalence rate; ASDAR, age-standardized DALYs rate; ASDR, age-standardized Deaths rate;

**Supplymental Table3** Prediction of future burden of atrial fibrillation/atrial flutter in different age groups in mainland China and the United States in male.

|  | Location | Age | Incidence Number(N) | Incidence ASR (per 100 000) | Prevalence Number(N) | Prevalence ASR (per 100 000) | Deaths Number(N) | Deaths ASR (per 100 000) | DALYs Number(N) | DALYs ASR (per 100 000) |
| --- | --- | --- | --- | --- | --- | --- | --- | --- | --- | --- |
| China | 2022 | 30 to 34 | 3191.21073743383 | 5.12538905644114 | 5535.42831930446 | 8.89042625661624 | 3.34992672152295 | 0.00538030207687889 | 697.813460262057 | 1.12075502589356 |
| China | 2022 | 35 to 39 | 8846.09006697455 | 15.4514959702321 | 34200.8997229824 | 59.7388292733844 | 3.41004554286823 | 0.00595633828787784 | 3073.857771 | 5.36911794865471 |
| China | 2022 | 40 to 44 | 15632.0189031217 | 31.4406215418838 | 85686.940432773 | 172.341824937964 | 23.3032823069397 | 0.0468698051247784 | 8347.3218289692 | 16.7889373816273 |
| China | 2022 | 45 to 49 | 28021.6363853851 | 52.5200010619312 | 202686.544527409 | 379.888504276315 | 85.0463894937355 | 0.159399558437423 | 20628.8252432422 | 38.6639063037211 |
| China | 2022 | 50 to 54 | 48100.6109247589 | 77.5633168238855 | 432767.75861871 | 697.847743044526 | 259.052140709903 | 0.417727402573291 | 45841.8056015792 | 73.9209424432498 |
| China | 2022 | 55 to 59 | 60875.059242322 | 107.221226302321 | 655038.267626732 | 1153.74025428558 | 508.010714721646 | 0.894775832420133 | 70575.090665006 | 124.306207857266 |
| China | 2022 | 60 to 64 | 48145.4894403579 | 140.939453238225 | 600786.163576736 | 1758.7207938241 | 543.928079396451 | 1.59227638979598 | 64033.5404736213 | 187.449588490316 |
| China | 2022 | 65 to 69 | 64234.4573733307 | 177.313250451736 | 918759.589485885 | 2536.15046903287 | 1025.92353655078 | 2.83196658646151 | 97340.1514241642 | 268.698442460085 |
| China | 2022 | 70 to 74 | 61515.4223741521 | 243.720452338536 | 889665.549410238 | 3524.80210269001 | 1922.44163843565 | 7.61659966933506 | 107389.851618545 | 425.472218232506 |
| China | 2022 | 75 to 79 | 47979.2934105292 | 334.914767268009 | 699422.037308018 | 4882.24715697304 | 2718.0207004627 | 18.9728777899286 | 96558.6289955314 | 674.018070275698 |
| China | 2022 | 80 to 84 | 35436.0804403708 | 424.170029125064 | 549201.418920935 | 6573.94325117923 | 5001.58511744609 | 59.868994498659 | 103360.274563123 | 1237.22291311504 |
| China | 2022 | 85 to 89 | 20019.0276254122 | 510.687656355621 | 329837.382117215 | 8414.18888088702 | 5528.32765774223 | 141.02826310677 | 78659.8659847641 | 2006.62206779782 |
| China | 2022 | 90 to 94 | 6308.2781339559 | 591.111615089352 | 107413.429100522 | 10065.0802341967 | 3958.77894015192 | 370.953874163988 | 42063.5411403511 | 3941.52686547098 |
| China | 2022 | 95 plus | 903.404317249701 | 673.966053616899 | 14488.4649309683 | 10808.8187603742 | 1078.23771722708 | 804.396885497253 | 9883.78431732318 | 7373.59229301283 |
| China | 2023 | 30 to 34 | 3067.6126826879 | 5.16454091424269 | 5358.81371466489 | 9.02193840747249 | 3.2399946497615 | 0.00545475803547625 | 678.961299856779 | 1.1430789265173 |
| China | 2023 | 35 to 39 | 9240.09300137618 | 15.5975160925013 | 35639.2246177578 | 60.1599333921157 | 3.50169854541989 | 0.00591095775823258 | 3189.32203085095 | 5.38365811826328 |
| China | 2023 | 40 to 44 | 16383.441416549 | 31.882733814522 | 89439.1365817068 | 174.051599522604 | 23.8153554930891 | 0.046345491193163 | 8677.93172373523 | 16.8875500680202 |
| China | 2023 | 45 to 49 | 26809.4207725643 | 53.2772618396602 | 193190.795974791 | 383.920141709829 | 80.5041326863189 | 0.159982559589389 | 19626.4836701586 | 39.0029056710142 |
| China | 2023 | 50 to 54 | 48560.5334885383 | 78.3693415282701 | 436782.881534309 | 704.901374791189 | 254.776076374687 | 0.411169975044718 | 46005.0182249847 | 74.245127190357 |
| China | 2023 | 55 to 59 | 61617.5214392192 | 108.030048988775 | 663605.98116671 | 1163.4578116778 | 508.638088928126 | 0.891762543851499 | 71220.99948 | 124.867211196196 |
| China | 2023 | 60 to 64 | 52653.768909567 | 142.386569658101 | 655640.814680232 | 1772.98697630739 | 584.650007069476 | 1.58101330030492 | 69561.9124075377 | 188.109650869968 |
| China | 2023 | 65 to 69 | 65466.6694639789 | 179.213949757697 | 934013.589217053 | 2556.84710741321 | 1029.22393086779 | 2.81748387914323 | 98498.9365891417 | 269.639247232299 |
| China | 2023 | 70 to 74 | 66630.1341069404 | 246.222604767324 | 962154.852669211 | 3555.51248979187 | 2054.35112842005 | 7.59157538441135 | 115702.43736744 | 427.562631958919 |
| China | 2023 | 75 to 79 | 51416.5444342083 | 337.074250832668 | 750681.275456835 | 4921.28227058324 | 2890.90675760515 | 18.9520754510008 | 103316.040295814 | 677.314612736583 |
| China | 2023 | 80 to 84 | 36321.7374294534 | 425.525498611935 | 564562.416141282 | 6614.10275575831 | 5074.20747961801 | 59.4466239953037 | 105632.330516194 | 1237.53028609238 |
| China | 2023 | 85 to 89 | 20727.5746858464 | 510.750666747976 | 342710.023988163 | 8444.75901817363 | 5729.88934374027 | 141.190894113908 | 81605.3066479632 | 2010.8461994389 |
| China | 2023 | 90 to 94 | 6715.24981248343 | 589.337711952573 | 114807.95577737 | 10075.6725157132 | 4238.9542564112 | 372.015289423927 | 45095.3920140061 | 3957.62121905487 |
| China | 2023 | 95 plus | 982.678091328245 | 670.536599087174 | 15827.1331463475 | 10799.7442162438 | 1163.44905966834 | 793.886810508521 | 10698.8535400619 | 7300.43025299173 |
| China | 2024 | 30 to 34 | 2926.45503076706 | 5.20369277204424 | 5147.72155251117 | 9.15345055832875 | 3.10952179884128 | 0.00552921399407361 | 655.399754704821 | 1.16540282714104 |
| China | 2024 | 35 to 39 | 9685.45732792965 | 15.7435362147705 | 37269.5845259037 | 60.581037510847 | 3.60851572201796 | 0.00586557722858732 | 3320.98319282509 | 5.39819828787186 |
| China | 2024 | 40 to 44 | 16943.535170611 | 32.3248460871602 | 92127.8639901681 | 175.761374107245 | 24.0178321776516 | 0.0458211772615475 | 8903.54265777739 | 16.986162754413 |
| China | 2024 | 45 to 49 | 25992.9585824242 | 54.0345226173893 | 186621.70106793 | 387.951779143343 | 77.239027346762 | 0.160565560741355 | 18925.1696633903 | 39.3419050383074 |
| China | 2024 | 50 to 54 | 47996.8021699508 | 79.1753662326547 | 431593.375928204 | 711.955006537853 | 245.279678802454 | 0.404612547516145 | 45204.5716150496 | 74.5693119374641 |
| China | 2024 | 55 to 59 | 62466.2930592232 | 108.838871675229 | 673324.84512396 | 1173.17536907002 | 510.083121794262 | 0.888749255282865 | 71987.4754896919 | 125.428214535126 |
| China | 2024 | 60 to 64 | 59882.6136573327 | 143.833686077977 | 744091.271899179 | 1787.25315879067 | 653.537762786126 | 1.56975021081386 | 78590.9345506456 | 188.769713249619 |
| China | 2024 | 65 to 69 | 64518.264965247 | 181.114649063658 | 918195.470164165 | 2577.54374579355 | 998.509912017861 | 2.80300117182494 | 96388.4234643082 | 270.580052004513 |
| China | 2024 | 70 to 74 | 71309.6391001301 | 248.724757196112 | 1028173.72089054 | 3586.22287689374 | 2169.33784243972 | 7.56655109948764 | 123181.962147561 | 429.653045685333 |
| China | 2024 | 75 to 79 | 55297.1442861828 | 339.233734397328 | 808561.644337356 | 4960.31738419343 | 3085.91167284479 | 18.9312731120731 | 110943.722382453 | 680.611155197467 |
| China | 2024 | 80 to 84 | 37515.40937 | 426.880968098805 | 584793.868520326 | 6654.26226033739 | 5187.2048599315 | 59.0242534919483 | 108784.391856961 | 1237.83765906973 |
| China | 2024 | 85 to 89 | 21260.1878378631 | 510.813677140331 | 352745.233529252 | 8475.32915546023 | 5883.16764038396 | 141.353525121046 | 83867.7107964283 | 2015.07033107998 |
| China | 2024 | 90 to 94 | 7154.99952547345 | 587.563808815794 | 122824.480941785 | 10086.2647972297 | 4543.10426361731 | 373.076704683866 | 48389.5240142512 | 3973.71557263876 |
| China | 2024 | 95 plus | 1060.13331877347 | 667.107144557449 | 17147.9927094392 | 10790.6696721135 | 1244.90313925127 | 783.37673551979 | 11485.2132806423 | 7227.26821297062 |
| China | 2025 | 30 to 34 | 2738.6524228876 | 5.23385241026579 | 4843.96615754896 | 9.25732807020544 | 2.92387294039793 | 0.00558782827222637 | 619.199656078533 | 1.18335557492371 |
| China | 2025 | 35 to 39 | 10073.6409660726 | 15.8164937557049 | 38882.4722847392 | 61.0488682462651 | 3.77495755293956 | 0.00592701216622744 | 3476.82410977372 | 5.45891669229895 |
| China | 2025 | 40 to 44 | 17403.707356342 | 32.4806200243702 | 94429.5220645196 | 176.234256440934 | 24.5469587694605 | 0.0458121033765939 | 9098.22569924178 | 16.9800609595601 |
| China | 2025 | 45 to 49 | 25736.7441228256 | 54.4519811197945 | 184246.860136212 | 389.816462472448 | 75.5943340516148 | 0.159937140102011 | 18633.5610392108 | 39.4235692385728 |
| China | 2025 | 50 to 54 | 46536.6130302856 | 79.8677021201279 | 417189.042544272 | 715.993881119432 | 236.315340417049 | 0.405572343706288 | 43614.3065502547 | 74.8523413481928 |
| China | 2025 | 55 to 59 | 63994.4843375065 | 109.634678507753 | 688755.857060184 | 1179.96930111785 | 511.226188776352 | 0.875827337800575 | 73259.9780107005 | 125.508224964019 |
| China | 2025 | 60 to 64 | 67279.1534689904 | 144.881970247722 | 834496.657493879 | 1797.04282335504 | 728.292496923979 | 1.5683379833192 | 87884.3760215216 | 189.254187894299 |
| China | 2025 | 65 to 69 | 61765.6617506962 | 182.912243104028 | 875924.860316393 | 2593.95554827433 | 940.716887830882 | 2.78583004216008 | 91560.4495308097 | 271.146244184857 |
| China | 2025 | 70 to 74 | 75647.2724713533 | 251.242691393315 | 1087299.23138751 | 3611.18089680138 | 2261.93122430258 | 7.51241479004497 | 129551.110965459 | 430.270236171193 |
| China | 2025 | 75 to 79 | 60310.8590080656 | 342.625109546814 | 880087.207506092 | 4999.76257745224 | 3316.11231896103 | 18.8387856721062 | 120137.105015558 | 682.497139712234 |
| China | 2025 | 80 to 84 | 39200.6885436756 | 429.879954223804 | 611597.798254966 | 6706.86264156554 | 5381.69957411296 | 59.0164319177295 | 113363.326128996 | 1243.15728262516 |
| China | 2025 | 85 to 89 | 21936.1413081143 | 512.955847126166 | 364897.247404117 | 8532.77584362331 | 6015.41936437605 | 140.664873212285 | 86221.1935605016 | 2016.20078763334 |
| China | 2025 | 90 to 94 | 7583.6054715226 | 588.209711978074 | 130690.484674752 | 10136.7895043433 | 4806.92074389009 | 372.840791478744 | 51255.2279746364 | 3975.52628463388 |
| China | 2025 | 95 plus | 1145.39051007832 | 666.029266267563 | 18612.3878285589 | 10822.8546507643 | 1344.50475060606 | 781.81153472118 | 12415.7568419301 | 7219.5965889588 |
| China | 2026 | 30 to 34 | 2588.06963184024 | 5.26401204848733 | 4602.46892697788 | 9.36120558208213 | 2.77609290365627 | 0.00564644255037913 | 590.627369359225 | 1.20130832270639 |
| China | 2026 | 35 to 39 | 10177.1417558863 | 15.8894512966394 | 39401.2451533294 | 61.5166989816832 | 3.83558084768961 | 0.00598844710386755 | 3535.30828544262 | 5.51963509672604 |
| China | 2026 | 40 to 44 | 17984.2493188593 | 32.6363939615802 | 97374.2762109748 | 176.707138774623 | 25.2397094760662 | 0.0458030294916403 | 9353.48169609643 | 16.9739591647072 |
| China | 2026 | 45 to 49 | 26111.3106281865 | 54.8694396221996 | 186393.521341714 | 391.681145801553 | 75.8119544925209 | 0.159308719462667 | 18799.7805128517 | 39.5052334388382 |
| China | 2026 | 50 to 54 | 44547.4920619613 | 80.5600380076012 | 398158.370604498 | 720.032755701012 | 224.801126251451 | 0.406532139896432 | 41547.7998178173 | 75.1353707589214 |
| China | 2026 | 55 to 59 | 65493.0256096352 | 110.430485340276 | 703833.860575689 | 1186.76323316568 | 511.763455693033 | 0.862905420318286 | 74482.6348585924 | 125.588235392913 |
| China | 2026 | 60 to 64 | 74887.3336536434 | 145.930254417467 | 927216.004105506 | 1806.83248791942 | 804.102564991303 | 1.56692575582453 | 97368.5860089331 | 189.73866253898 |
| China | 2026 | 65 to 69 | 58843.5392421268 | 184.709837144398 | 831592.166477007 | 2610.36735075512 | 882.019560431504 | 2.76865891249522 | 86560.2052333061 | 271.712436365202 |
| China | 2026 | 70 to 74 | 78947.1104249839 | 253.760625590518 | 1131234.05142147 | 3636.13891670901 | 2320.33450192753 | 7.45827848060231 | 134052.779756004 | 430.887426657054 |
| China | 2026 | 75 to 79 | 65750.7409947956 | 346.016484696301 | 957560.288613977 | 5039.20777071105 | 3562.20889520458 | 18.7462982321392 | 130047.843188032 | 684.383124227001 |
| China | 2026 | 80 to 84 | 41302.3952128143 | 432.878940348804 | 644942.470448831 | 6759.4630227937 | 5630.20447103017 | 59.0086103435106 | 119121.264138174 | 1248.4769061806 |
| China | 2026 | 85 to 89 | 22825.4045067997 | 515.098017112001 | 380656.297593192 | 8590.22253178639 | 6202.72990080549 | 139.976221303523 | 89393.4749175212 | 2017.33124418669 |
| China | 2026 | 90 to 94 | 8011.0685505085 | 588.855615140355 | 138593.010570338 | 10187.3142114568 | 5069.09188832714 | 372.604878273622 | 54109.5618505293 | 3977.336997 |
| China | 2026 | 95 plus | 1230.87821525772 | 664.951387977677 | 20093.5467572178 | 10855.0396294151 | 1444.29838379739 | 780.246333922571 | 13349.850064114 | 7211.92496494697 |
| China | 2027 | 30 to 34 | 2508.58088840453 | 5.29417168670888 | 4484.91812539354 | 9.46508309395882 | 2.7032739726304 | 0.00570505682853189 | 577.732495355832 | 1.21926107048906 |
| China | 2027 | 35 to 39 | 9877.8203252067 | 15.9624088375739 | 38357.1210158918 | 61.9845297171013 | 3.74377378770298 | 0.00604988204150767 | 3453.22123975286 | 5.58035350115314 |
| China | 2027 | 40 to 44 | 18626.3743854787 | 32.7921678987901 | 100640.537611794 | 177.180021108312 | 26.0115575266254 | 0.0457939556066867 | 9637.96187143733 | 16.9678573698543 |
| China | 2027 | 45 to 49 | 27157.3141314377 | 55.2868981246048 | 193312.485767074 | 393.545829130658 | 77.9448814781232 | 0.158680298823324 | 19445.363207956 | 39.5868976391036 |
| China | 2027 | 50 to 54 | 42501.9199370459 | 81.2523738950744 | 378751.20422571 | 724.071630282591 | 213.153029410669 | 0.407491936086575 | 39450.2542157094 | 75.41840017 |
| China | 2027 | 55 to 59 | 66921.5232116085 | 111.2262922 | 718127.539594015 | 1193.55716521351 | 511.409574151334 | 0.849983502835996 | 75610.8134636062 | 125.668245821806 |
| China | 2027 | 60 to 64 | 79480.6363002329 | 146.978538587212 | 982363.044185774 | 1816.62215248379 | 846.572653152681 | 1.56551352832986 | 102865.738955573 | 190.223137183661 |
| China | 2027 | 65 to 69 | 58698.3162802628 | 186.507431184768 | 826709.759260436 | 2626.7791532359 | 865.958525576682 | 2.75148778283037 | 85692.5482979253 | 272.278628545547 |
| China | 2027 | 70 to 74 | 81660.0569802125 | 256.27855978772 | 1166564.16635802 | 3661.09693661664 | 2359.24016463691 | 7.40414217115964 | 137493.71641663 | 431.504617142915 |
| China | 2027 | 75 to 79 | 71773.1053947471 | 349.407859845787 | 1043224.08376054 | 5078.65296396986 | 3831.74530931031 | 18.6538107921723 | 140968.967019291 | 686.269108741768 |
| China | 2027 | 80 to 84 | 44139.1084839015 | 435.877926473804 | 689822.510678128 | 6812.06340402185 | 5974.70543459613 | 59.0007887692918 | 126965.504976275 | 1253.79652973604 |
| China | 2027 | 85 to 89 | 23704.7453617591 | 517.240187097836 | 396316.454028446 | 8647.66921994946 | 6383.44901831197 | 139.287569394762 | 92504.6464524915 | 2018.46170074004 |
| China | 2027 | 90 to 94 | 8440.4591589964 | 589.501518302635 | 146584.968122534 | 10237.8389185704 | 5331.56394709217 | 372.368965068499 | 56973.2777261718 | 3979.14770862412 |
| China | 2027 | 95 plus | 1318.1341309553 | 663.873509687791 | 21616.8022038071 | 10887.2246080659 | 1546.08696344029 | 778.681133123961 | 14304.1890934935 | 7204.25334093514 |
| China | 2028 | 30 to 34 | 2445.74905791354 | 5.32433132493043 | 4395.53344048133 | 9.56896060583551 | 2.64756122769683 | 0.00576367110668466 | 568.318225484446 | 1.23721381827174 |
| China | 2028 | 35 to 39 | 9466.71121471856 | 16.0353663785084 | 36869.657176895 | 62.4523604525194 | 3.60790465384926 | 0.00611131697914779 | 3330.28865141264 | 5.64107190558023 |
| China | 2028 | 40 to 44 | 19369.3668558699 | 32.9479418360001 | 104438.215804387 | 177.652903442001 | 26.9159201183208 | 0.0457848817217331 | 9971.44124774513 | 16.9617555750014 |
| China | 2028 | 45 to 49 | 28287.2460500689 | 55.70435663 | 200793.530955346 | 395.410512459763 | 80.2603716761702 | 0.15805187818398 | 20144.1042882185 | 39.668561839369 |
| China | 2028 | 50 to 54 | 40443.4468592178 | 81.9447097825476 | 359355.699583965 | 728.110504864171 | 201.589534854966 | 0.408451732276718 | 37362.1036980343 | 75.7014295803787 |
| China | 2028 | 55 to 59 | 67381.0113762335 | 112.022099005323 | 722008.172121446 | 1200.35109726135 | 503.490442565675 | 0.837061585353706 | 75637.2605066728 | 125.7482563 |
| China | 2028 | 60 to 64 | 80497.2437819051 | 148.026822756958 | 993205.924067352 | 1826.41181704816 | 850.561008930442 | 1.56410130083519 | 103707.131143497 | 190.707611828341 |
| China | 2028 | 65 to 69 | 64408.2368533079 | 188.305025225138 | 904082.453034794 | 2643.19095571669 | 935.251273397872 | 2.73431665316551 | 93324.4018129666 | 272.844820725892 |
| China | 2028 | 70 to 74 | 83361.7150669656 | 258.796493984923 | 1187326.2202109 | 3686.05495652427 | 2367.53243813524 | 7.35000586171698 | 139192.051820728 | 432.121807628775 |
| China | 2028 | 75 to 79 | 77993.7678794489 | 352.799234995274 | 1131464.35724124 | 5118.09815722867 | 4103.37495512622 | 18.5613233522053 | 152131.306660089 | 688.155093256535 |
| China | 2028 | 80 to 84 | 47637.4775449701 | 438.876912598803 | 745118.409139366 | 6864.66378525001 | 6403.33558087035 | 58.9929671950729 | 136669.566698677 | 1259.11615329147 |
| China | 2028 | 85 to 89 | 24527.7435182757 | 519.382357083672 | 411097.61889091 | 8705.11590811254 | 6545.3103164604 | 138.5989175 | 95374.8962975137 | 2019.59215729339 |
| China | 2028 | 90 to 94 | 8842.22012612834 | 590.147421464916 | 154151.272389076 | 10288.363625684 | 5575.69556538261 | 372.133051863377 | 59646.9786832275 | 3980.95842061924 |
| China | 2028 | 95 plus | 1433.46125180582 | 662.795631397905 | 23615.9530836717 | 10919.4095867168 | 1680.70748263665 | 777.115932325351 | 15564.4071082759 | 7196.58171692331 |
| China | 2029 | 30 to 34 | 2392.0085352743 | 5.35449096315197 | 4321.1411686227 | 9.6728381177122 | 2.60098605659719 | 0.00582228538483742 | 560.719806953548 | 1.25516656605441 |
| China | 2029 | 35 to 39 | 9004.16292736321 | 16.1083239194428 | 35170.8629470265 | 62.9201911879376 | 3.45041881743301 | 0.0061727519167879 | 3187.16268593279 | 5.70179031000733 |
| China | 2029 | 40 to 44 | 20213.049071167 | 33.10371577 | 108763.175511493 | 178.125785775691 | 27.9505979454406 | 0.0457758078367794 | 10353.0813350331 | 16.9556537801485 |
| China | 2029 | 45 to 49 | 29076.7472140754 | 56.1218151294152 | 205828.525248834 | 397.275195788868 | 81.5611909564196 | 0.157423457544636 | 20594.6167562747 | 39.7502260396344 |
| China | 2029 | 50 to 54 | 39008.4166188618 | 82.6370456700208 | 345607.563642876 | 732.14937944575 | 193.260726366869 | 0.409411528466862 | 35868.0953421261 | 75.9844589911073 |
| China | 2029 | 55 to 59 | 66427.3454151087 | 112.817905837847 | 710768.731546087 | 1207.14502930918 | 485.254623203812 | 0.824139667871416 | 74087.8646136487 | 125.828266679593 |
| China | 2029 | 60 to 64 | 81681.6231640591 | 149.075106926703 | 1006096.32665302 | 1836.20148161253 | 856.232691310114 | 1.56268907334053 | 104758.468943565 | 191.192086473022 |
| China | 2029 | 65 to 69 | 73389.0857510303 | 190.102619265508 | 1026739.21926569 | 2659.60275819747 | 1048.95359460415 | 2.71714552350066 | 105550.277786691 | 273.411012906237 |
| China | 2029 | 70 to 74 | 82218.9368780885 | 261.314428182126 | 1167618.42729315 | 3711.0129764319 | 2295.54350428416 | 7.29586955227431 | 136155.284720356 | 432.738998114636 |
| China | 2029 | 75 to 79 | 83691.1299122135 | 356.19061014476 | 1211824.84400163 | 5157.54335048748 | 4339.46797483042 | 18.4688359122384 | 162133.183300513 | 690.041077771302 |
| China | 2029 | 80 to 84 | 51557.0547110083 | 441.875898723803 | 807090.334891806 | 6917.26416647816 | 6882.24994550341 | 58.9851456208541 | 147531.432951495 | 1264.43577684691 |
| China | 2029 | 85 to 89 | 25567.286213244 | 521.524527069507 | 429577.007852953 | 8762.56259627562 | 6760.93078799309 | 137.910265577239 | 99064.1681151626 | 2020.72261384675 |
| China | 2029 | 90 to 94 | 9168.72838255308 | 590.793324627196 | 160452.826647601 | 10338.8883327975 | 5771.60185883598 | 371.897138658255 | 61809.9881382386 | 3982.76913261436 |
| China | 2029 | 95 plus | 1555.34110997027 | 661.717753108019 | 25741.2849621139 | 10951.5945653676 | 1822.90097242434 | 775.550731526741 | 16897.2456169847 | 7188.91009291148 |
| China | 2030 | 30 to 34 | 2355.32732790844 | 5.3749828655632 | 4270.1325385368 | 9.74467071152304 | 2.56905606372441 | 0.00586272326549711 | 555.524847148863 | 1.26773739659842 |
| China | 2030 | 35 to 39 | 8403.51312546987 | 16.1577090022782 | 32890.701458611 | 63.2400253458674 | 3.23244051543621 | 0.00621511889560739 | 2987.29243079746 | 5.74376467090183 |
| China | 2030 | 40 to 44 | 20985.1180461654 | 33.1915172451784 | 113082.874839809 | 178.859712970055 | 29.0739162012856 | 0.0459853210669061 | 10783.3818741436 | 17.0557442016574 |
| China | 2030 | 45 to 49 | 29822.8723049095 | 56.2961687330602 | 210568.639570653 | 397.487121359834 | 83.2349955390289 | 0.157121396807554 | 20999.7939531856 | 39.6409819839324 |
| China | 2030 | 50 to 54 | 38576.7962804553 | 83.1296017320822 | 340664.485000757 | 734.101991168345 | 189.098873012889 | 0.407491433121193 | 35248.7514647933 | 75.9579579786409 |
| China | 2030 | 55 to 59 | 64335.3924056166 | 113.615902826292 | 686065.90851194 | 1211.58812714601 | 467.03446086406 | 0.824779952961449 | 71353.5065997738 | 126.009848840952 |
| China | 2030 | 60 to 64 | 83655.9721522696 | 149.948509676956 | 1028494.78145777 | 1843.51763206347 | 859.539398060189 | 1.54067484283317 | 106536.775606636 | 190.961031436296 |
| China | 2030 | 65 to 69 | 82438.5355240231 | 191.230615748986 | 1150947.95727342 | 2669.82528456364 | 1169.23533253258 | 2.71224605306725 | 117948.483227511 | 273.602156211074 |
| China | 2030 | 70 to 74 | 78720.7552724455 | 263.485354553582 | 1114025.40869708 | 3728.74191534838 | 2166.96056566815 | 7.25300933626277 | 129336.160008849 | 432.899606445453 |
| China | 2030 | 75 to 79 | 88946.3861778814 | 359.239329099784 | 1283883.16733293 | 5185.38579805595 | 4542.33985952537 | 18.3457383014498 | 170836.817673471 | 689.980857050505 |
| China | 2030 | 80 to 84 | 56501.3724604372 | 445.702014188419 | 882529.611004563 | 6961.69328419552 | 7444.3675101787 | 58.7236990741906 | 160485.066826744 | 1265.96070886469 |
| China | 2030 | 85 to 89 | 26924.5211612679 | 524.63624887409 | 452626.093758883 | 8819.62039547039 | 7073.58366702389 | 137.831918307352 | 103990.117083099 | 2026.2936012744 |
| China | 2030 | 90 to 94 | 9552.4403574544 | 592.754871135397 | 167544.59278666 | 10396.5970778545 | 5966.93379141202 | 370.264449527997 | 64152.2155510184 | 3980.81922933123 |
| China | 2030 | 95 plus | 1669.51016222986 | 661.947156451658 | 27732.1095782786 | 10995.5551592623 | 1954.33245097413 | 774.87686984526 | 18122.8593737384 | 7185.56586274181 |
| China | 2031 | 30 to 34 | 2332.33835136173 | 5.39547476797442 | 4243.44624335902 | 9.81650330533388 | 2.55179937401855 | 0.00590316114615681 | 553.447492230471 | 1.28030822714242 |
| China | 2031 | 35 to 39 | 7920.23929804216 | 16.2070940851136 | 31061.0461305585 | 63.5598595037973 | 3.05796864442847 | 0.00625748587442687 | 2827.43084029728 | 5.78573903179634 |
| China | 2031 | 40 to 44 | 21161.7224068423 | 33.2793187171468 | 114200.377462523 | 179.59364016442 | 29.3744673180525 | 0.0461948342970328 | 10909.087804315 | 17.1558346231663 |
| China | 2031 | 45 to 49 | 30774.0205586745 | 56.4705223367053 | 216728.97894303 | 397.699046930801 | 85.45983413 | 0.156819336070471 | 21543.1071890891 | 39.5317379282304 |
| China | 2031 | 50 to 54 | 39090.6903857069 | 83.6221577941436 | 344082.039349136 | 736.05460289094 | 189.591658628669 | 0.405571337775524 | 35495.5331588948 | 75.9314569661746 |
| China | 2031 | 55 to 59 | 61519.7292304912 | 114.413899814737 | 653853.350142782 | 1216.03122498283 | 443.823955205782 | 0.825420238051482 | 67852.4434447294 | 126.191431002311 |
| China | 2031 | 60 to 64 | 85580.7601274802 | 150.82191242721 | 1050217.1695618 | 1850.83378251441 | 861.732406696754 | 1.51866061232581 | 108225.761739026 | 190.72997639957 |
| China | 2031 | 65 to 69 | 91689.9965814704 | 192.358612232465 | 1277476.33324243 | 2680.04781092982 | 1290.4886140817 | 2.70734658263384 | 130506.798761394 | 273.793299515911 |
| China | 2031 | 70 to 74 | 75029.6195526329 | 265.656280925038 | 1058120.22167034 | 3746.47085426486 | 2036.37099611001 | 7.21014912025124 | 122309.711800917 | 433.060214776269 |
| China | 2031 | 75 to 79 | 92975.9091447207 | 362.288048054807 | 1337898.49904886 | 5213.22824562443 | 4676.57322489285 | 18.2226406906612 | 177058.003279078 | 689.920636329708 |
| China | 2031 | 80 to 84 | 61874.2789772377 | 449.528129653035 | 964341.814113445 | 7006.12240191287 | 8046.90403983823 | 58.4622525275271 | 174460.183846862 | 1267.48564088246 |
| China | 2031 | 85 to 89 | 28633.8580432419 | 527.747970678673 | 481619.177037707 | 8876.67819466516 | 7474.0528001727 | 137.753571037465 | 110242.247111185 | 2031.86458870206 |
| China | 2031 | 90 to 94 | 10056.5356790697 | 594.716417643598 | 176780.220604268 | 10454.3058229114 | 6233.4893419737 | 368.63176039774 | 67281.8845296082 | 3978.8693260481 |
| China | 2031 | 95 plus | 1785.32070992649 | 662.176559795296 | 29764.0800027167 | 11039.515753157 | 2087.35969843069 | 774.203008163779 | 19364.2750324431 | 7182.22163257214 |
| China | 2032 | 30 to 34 | 2312.10882986665 | 5.41596667038564 | 4221.39022201035 | 9.88833589914472 | 2.5373582644501 | 0.00594359902681651 | 551.937865787055 | 1.29287905768643 |
| China | 2032 | 35 to 39 | 7656.88134485188 | 16.2564791679489 | 30087.6487251725 | 63.8796936617272 | 2.96726156315801 | 0.00629985285324635 | 2744.88156375301 | 5.82771339269084 |
| China | 2032 | 40 to 44 | 20500.9128002877 | 33.3671201891152 | 110794.090498599 | 180.327567358785 | 28.5110455087724 | 0.0464043475271595 | 10602.1200698221 | 17.2559250446752 |
| China | 2032 | 45 to 49 | 31829.1653333359 | 56.6448759403503 | 223589.052345075 | 397.910972501768 | 87.9481886297321 | 0.156517275333389 | 22151.7843316044 | 39.4224938725284 |
| China | 2032 | 50 to 54 | 40607.1511283033 | 84.1147138562049 | 356279.765140986 | 738.007214613534 | 194.866346837931 | 0.403651242429855 | 36643.8150531979 | 75.9049559537082 |
| China | 2032 | 55 to 59 | 58633.5325261305 | 115.211896803182 | 621122.669533005 | 1220.47432281966 | 420.397961461499 | 0.826060523141515 | 64313.6375960828 | 126.37301316367 |
| China | 2032 | 60 to 64 | 87389.1119737699 | 151.695315177464 | 1070448.82939197 | 1858.14993296535 | 862.192732141144 | 1.49664638181844 | 109743.214880331 | 190.498921362843 |
| China | 2032 | 65 to 69 | 97249.8368206614 | 193.486608715944 | 1352178.08116941 | 2690.27033729599 | 1358.29834644419 | 2.70244711220043 | 137709.490762923 | 273.984442820748 |
| China | 2032 | 70 to 74 | 75038.8333013928 | 267.827207296495 | 1054639.53287232 | 3764.19979318134 | 2008.10441454805 | 7.1672889042397 | 121378.20296889 | 433.220823107086 |
| China | 2032 | 75 to 79 | 96367.5532993174 | 365.33676700983 | 1382475.58138097 | 5241.0706931929 | 4774.24896683333 | 18.0995430798726 | 181969.531603405 | 689.860415608912 |
| China | 2032 | 80 to 84 | 67853.42106 | 453.354245117651 | 1055254.35381069 | 7050.55151963023 | 8710.90066296241 | 58.2008059808636 | 189932.507883442 | 1269.01057290024 |
| China | 2032 | 85 to 89 | 30891.5854983025 | 530.859692483256 | 519868.57013502 | 8933.73599385993 | 8011.54430489784 | 137.675223767577 | 118561.676820622 | 2037.43557612972 |
| China | 2032 | 90 to 94 | 10557.7631004133 | 596.6779642 | 186002.108649129 | 10512.0145679684 | 6493.76964678394 | 366.999071267483 | 70368.5667270649 | 3976.91942276497 |
| China | 2032 | 95 plus | 1904.80796354269 | 662.405963138935 | 31871.5337488185 | 11083.4763470517 | 2224.35267833303 | 773.529146482298 | 20643.5080695745 | 7178.87740240247 |
| China | 2033 | 30 to 34 | 2303.08070560167 | 5.43645857279687 | 4219.4880313171 | 9.96016849295556 | 2.53505471598334 | 0.00598403690747621 | 553.035842994866 | 1.30544988823044 |
| China | 2033 | 35 to 39 | 7445.76913186967 | 16.3058642507843 | 29315.5183416431 | 64.1995278196571 | 2.89605653075739 | 0.00634221983206584 | 2680.2835603919 | 5.86968775358535 |
| China | 2033 | 40 to 44 | 19611.0977666424 | 33.4549216610836 | 106137.288481135 | 181.06149455315 | 27.3247981224591 | 0.0466138607572861 | 10174.0042793951 | 17.3560154661841 |
| China | 2033 | 45 to 49 | 33052.7992542579 | 56.8192295439954 | 231595.47100744 | 398.122898072735 | 90.8732865607504 | 0.156215214596307 | 22869.2462860995 | 39.3132498168264 |
| China | 2033 | 50 to 54 | 42241.1626431709 | 84.6072699182663 | 369433.541631495 | 739.959826336129 | 200.568943297709 | 0.401731147084187 | 37883.2003370614 | 75.8784549412419 |
| China | 2033 | 55 to 59 | 55739.4604459922 | 116.009893791627 | 588538.045219871 | 1224.91742065649 | 397.206268319313 | 0.826700808231548 | 60805.8901687151 | 126.554595325029 |
| China | 2033 | 60 to 64 | 87931.7569765223 | 152.568717927718 | 1075146.41613899 | 1865.46608341629 | 849.892413858289 | 1.47463215131108 | 109659.35880877 | 190.267866326117 |
| China | 2033 | 65 to 69 | 98526.0395879974 | 194.614605199422 | 1367157.75529612 | 2700.49286366217 | 1365.66670045602 | 2.69754764176702 | 138804.765577543 | 274.175586125585 |
| China | 2033 | 70 to 74 | 82705.3909081901 | 269.998133667951 | 1158474.28249166 | 3781.92873209782 | 2182.34345420358 | 7.12442868822817 | 132752.698000139 | 433.381431437903 |
| China | 2033 | 75 to 79 | 98577.6904734561 | 368.385485964853 | 1409928.75265193 | 5268.91314076138 | 4810.38625238724 | 17.976445469084 | 184586.289880861 | 689.800194888115 |
| China | 2033 | 80 to 84 | 74027.4120484865 | 457.180360582267 | 1148831.18436679 | 7094.98063734758 | 9381.63841770953 | 57.9393594342001 | 205727.243455974 | 1270.53550491802 |
| China | 2033 | 85 to 89 | 33617.0941570377 | 533.971414287839 | 566030.977315021 | 8990.79379305471 | 8662.64940250843 | 137.59687649769 | 128621.01261011 | 2043.00656355738 |
| China | 2033 | 90 to 94 | 11033.7103992228 | 598.639510660001 | 194813.846996597 | 10569.7233130253 | 6734.18105274967 | 365.366382137226 | 73263.8954541209 | 3974.96951948184 |
| China | 2033 | 95 plus | 2026.57087308198 | 662.635366482574 | 34031.5967683436 | 11127.4369409464 | 2363.66196027058 | 772.855284800817 | 21945.2918772982 | 7175.5331722328 |
| China | 2034 | 30 to 34 | 2307.11418732462 | 5.45695047520809 | 4241.37476410801 | 10.0320010867664 | 2.54705468155414 | 0.00602447478813591 | 557.238756937109 | 1.31802071877444 |
| China | 2034 | 35 to 39 | 7263.47891672842 | 16.3552493336197 | 28653.4931926504 | 64.519361977587 | 2.8354389925173 | 0.00638458681088532 | 2625.40987639247 | 5.91166211447985 |
| China | 2034 | 40 to 44 | 18618.4982817071 | 33.542723133052 | 100908.853881725 | 181.795421747514 | 25.9901649806526 | 0.0468233739874128 | 9689.328925 | 17.4561058876929 |
| China | 2034 | 45 to 49 | 34443.204532941 | 56.9935831476404 | 240727.588013055 | 398.334823643701 | 94.2237415366207 | 0.155913153859225 | 23692.3441967678 | 39.2040057611244 |
| China | 2034 | 50 to 54 | 43361.6172563189 | 85.0998259803277 | 378032.7728 | 741.912438058724 | 203.719027631614 | 0.399811051738518 | 38649.4726224684 | 75.8519539287755 |
| China | 2034 | 55 to 59 | 53715.4693871439 | 116.807890780073 | 565335.756479192 | 1229.36051849332 | 380.462440287666 | 0.827341093321581 | 58281.1076935831 | 126.736177486388 |
| China | 2034 | 60 to 64 | 86641.8867365805 | 153.442120677971 | 1057476.17063728 | 1872.78223386723 | 820.228219016472 | 1.45261792080372 | 107305.267984869 | 190.036811289391 |
| China | 2034 | 65 to 69 | 100047.024879954 | 195.742601682901 | 1385487.92003888 | 2710.71539002834 | 1376.25348940025 | 2.6926481713336 | 140233.014017178 | 274.366729430422 |
| China | 2034 | 70 to 74 | 94452.758584111 | 272.169060039407 | 1318622.14114498 | 3799.6576710143 | 2457.56691523381 | 7.08156847221663 | 150455.167874001 | 433.542039768719 |
| China | 2034 | 75 to 79 | 97312.1587772199 | 371.434204919876 | 1387698.58561321 | 5296.75558832985 | 4677.40396139917 | 17.8533478582954 | 180705.182754088 | 689.739974167319 |
| China | 2034 | 80 to 84 | 79671.3604735308 | 461.006476046883 | 1233836.221 | 7139.40975506493 | 9967.92459062226 | 57.6779128875366 | 219838.095300254 | 1272.0604369358 |
| China | 2034 | 85 to 89 | 36651.7670148345 | 537.083136092423 | 617445.840799703 | 9047.85159224948 | 9384.57522638134 | 137.518529227803 | 139799.561864484 | 2048.57755098504 |
| China | 2034 | 90 to 94 | 11620.2971218578 | 600.601057168202 | 205617.217424724 | 10627.4320580823 | 7037.43947088636 | 363.733693006968 | 76869.1094698972 | 3973.01961619871 |
| China | 2034 | 95 plus | 2132.85356933592 | 662.864769826212 | 35945.423850031 | 11171.3975348412 | 2484.59411247147 | 772.181423119336 | 23077.4503056506 | 7172.18894206313 |
| China | 2035 | 30 to 34 | 2307.1018258076 | 5.46731339216444 | 4248.80279407801 | 10.0687087829757 | 2.55092124974368 | 0.00604511069042148 | 558.910414639952 | 1.3244922095762 |
| China | 2035 | 35 to 39 | 7136.18331206065 | 16.3801679458003 | 28179.2265429374 | 64.6816993302239 | 2.79092573296327 | 0.00640620205943018 | 2584.81748863871 | 5.93310775825879 |
| China | 2035 | 40 to 44 | 17347.9288843116 | 33.5869718032298 | 94090.5995552383 | 182.166893540166 | 24.2394777575327 | 0.046929559222687 | 9042.51525001928 | 17.5070296147761 |
| China | 2035 | 45 to 49 | 35714.5754342444 | 57.0611863666455 | 249752.836037628 | 399.030170440631 | 97.7597996393383 | 0.156190857053771 | 24598.6079027094 | 39.3012021795082 |
| China | 2035 | 50 to 54 | 44430.3377457796 | 85.2491645401315 | 386260.236385313 | 741.123388152714 | 207.738822232263 | 0.398591636624089 | 39352.8658406615 | 75.5069419992185 |
| China | 2035 | 55 to 59 | 53082.8533125743 | 117.334900262239 | 556727.420689495 | 1230.59617754914 | 372.299245388435 | 0.822934188713084 | 57206.6934084854 | 126.450280015885 |
| China | 2035 | 60 to 64 | 83863.1946536918 | 154.309269322092 | 1019925.70958456 | 1876.67535989676 | 789.11159194472 | 1.45197465550189 | 103231.290385021 | 189.946794374687 |
| China | 2035 | 65 to 69 | 102517.815809839 | 196.643315721384 | 1416711.25122599 | 2717.44765200248 | 1384.65798364401 | 2.65596506219862 | 142639.960432089 | 273.603124999871 |
| China | 2035 | 70 to 74 | 106133.401514916 | 273.430583138477 | 1478477.30640186 | 3808.98855851379 | 2742.393162 | 7.06520426850028 | 168132.109220444 | 433.157328534194 |
| China | 2035 | 75 to 79 | 93267.9157025888 | 373.996286349171 | 1325397.45544223 | 5314.72932077406 | 4427.393259 | 17.7534645704643 | 171828.038005269 | 689.015591487597 |
| China | 2035 | 80 to 84 | 84966.4495445481 | 464.380594105974 | 1311582.61865865 | 7168.4002207534 | 10491.3928025132 | 57.3402707627066 | 232441.942754412 | 1270.40176505018 |
| China | 2035 | 85 to 89 | 40479.0496972795 | 541.134104912425 | 680288.965562831 | 9094.27378396145 | 10243.7734250824 | 136.941336438543 | 153238.039604431 | 2048.52460766764 |
| China | 2035 | 90 to 94 | 12378.8463853974 | 603.630330491458 | 219103.868417907 | 10684.173337919 | 7452.91590931883 | 363.426926338964 | 81591.6850199008 | 3978.65958269071 |
| China | 2035 | 95 plus | 2257.04780546974 | 664.529482305029 | 38117.1365583098 | 11222.6072317383 | 2611.88702105075 | 769.002732565893 | 24328.3201546655 | 7162.84606757198 |
| China | 2036 | 30 to 34 | 2316.03346538621 | 5.47767630912078 | 4272.70277151782 | 10.1054164791851 | 2.5646773026493 | 0.00606574659270704 | 562.748928073182 | 1.33096370037795 |
| China | 2036 | 35 to 39 | 7050.93802848813 | 16.4050865579809 | 27870.0927637247 | 64.8440366828608 | 2.76268834893325 | 0.00642781730797505 | 2559.27860402941 | 5.95455340203772 |
| China | 2036 | 40 to 44 | 16323.7442205385 | 33.6312204734076 | 88599.5079626744 | 182.538365332818 | 22.8299612962744 | 0.0470357444579611 | 8522.18668707797 | 17.5579533418592 |
| China | 2036 | 45 to 49 | 35969.1691342125 | 57.1287895856505 | 251673.365409265 | 399.725517237561 | 98.5150245362822 | 0.156468560248318 | 24805.8408564751 | 39.398398597892 |
| China | 2036 | 50 to 54 | 45804.6673449632 | 85.3985030999354 | 397088.553739175 | 740.334338246704 | 213.136082690807 | 0.397372221509659 | 40314.1399307365 | 75.1619300696615 |
| China | 2036 | 55 to 59 | 53750.6811434024 | 117.861909744406 | 561774.37151011 | 1231.83183660496 | 373.287681749692 | 0.818527284104587 | 57537.0067611931 | 126.164382545381 |
| China | 2036 | 60 to 64 | 80148.6788465871 | 155.176417966212 | 971314.337596955 | 1880.56848592629 | 749.613214544261 | 1.45133139020006 | 98061.097703657 | 189.856777459984 |
| China | 2036 | 65 to 69 | 104914.514130817 | 197.544029759867 | 1446796.50621287 | 2724.17991397662 | 1391.08579394342 | 2.61928195306363 | 144903.522374284 | 272.83952056932 |
| China | 2036 | 70 to 74 | 117954.41110183 | 274.692106237547 | 1639608.89820283 | 3818.31944601327 | 3026.8135119746 | 7.04884006478393 | 185835.11522692 | 432.772617299668 |
| China | 2036 | 75 to 79 | 89028.4070642446 | 376.558367778465 | 1260792.74502795 | 5332.70305321828 | 4173.77584740498 | 17.6535812826332 | 162730.336542512 | 688.291208807875 |
| China | 2036 | 80 to 84 | 89081.8782641213 | 467.754712165064 | 1370712.17942681 | 7197.39068644186 | 10855.9060828068 | 57.0026286378767 | 241626.67918532 | 1268.74309316455 |
| China | 2036 | 85 to 89 | 44648.3132428687 | 545.185073732427 | 748583.695414958 | 9140.69597567343 | 11167.6369990586 | 136.364143649284 | 167761.020882127 | 2048.47166435023 |
| China | 2036 | 90 to 94 | 13348.7073916895 | 606.659603814714 | 236339.003701543 | 10740.9146177558 | 7989.95800773913 | 363.12015967096 | 87669.0132463877 | 3984.29954918271 |
| China | 2036 | 95 plus | 2418.49810920769 | 666.194194783845 | 40927.5630723638 | 11273.8169286354 | 2780.18633619864 | 765.82404201245 | 25969.5057119052 | 7153.50319308082 |
| China | 2037 | 30 to 34 | 2348.37989542085 | 5.48803922607713 | 4339.90347539532 | 10.1421241753944 | 2.60441620372652 | 0.00608638249499261 | 572.300194115011 | 1.3374351911797 |
| China | 2037 | 35 to 39 | 6974.41791289605 | 16.4300051701616 | 27594.7338317933 | 65.0063740354977 | 2.73773729736234 | 0.00644943255651991 | 2536.7682091961 | 5.97599904581665 |
| China | 2037 | 40 to 44 | 15755.3423571452 | 33.6754691435854 | 85575.8561852256 | 182.90983712547 | 22.0557355422878 | 0.0471419296932353 | 8238.45647296401 | 17.6088770689423 |
| China | 2037 | 45 to 49 | 34800.3524599884 | 57.1963928046555 | 243630.524888611 | 400.420864034491 | 95.3700914885993 | 0.156746263442864 | 24030.5473787061 | 39.4955950162758 |
| China | 2037 | 50 to 54 | 47334.2393012289 | 85.5478416597392 | 409195.754951341 | 739.545288340695 | 219.194211963271 | 0.39615280639523 | 41396.741733238 | 74.8169181401045 |
| China | 2037 | 55 to 59 | 55794.2457969667 | 118.388919226573 | 581119.173877093 | 1233.06749566079 | 383.678074423448 | 0.81412037949609 | 59323.9230723642 | 125.878485074878 |
| China | 2037 | 60 to 64 | 76348.2129323987 | 156.043566610332 | 922019.917500454 | 1884.46161195582 | 709.785403295821 | 1.45068812489823 | 92848.1279704567 | 189.76676054528 |
| China | 2037 | 65 to 69 | 107137.421867918 | 198.444743798351 | 1474379.63978717 | 2730.91217595076 | 1394.30743571994 | 2.58259884392865 | 146889.817535705 | 272.075916138769 |
| China | 2037 | 70 to 74 | 125030.670910885 | 275.953629336617 | 1734254.0134799 | 3827.65033351276 | 3186.31495149249 | 7.03247586106758 | 195908.82032985 | 432.387906065142 |
| China | 2037 | 75 to 79 | 89458.7473472236 | 379.120449207758 | 1262566.66899792 | 5350.67678566249 | 4142.03938934964 | 17.5536979948021 | 162240.963555158 | 687.566826128153 |
| China | 2037 | 80 to 84 | 92684.3550058334 | 471.128830224155 | 1421633.39015539 | 7226.38115213032 | 11147.6041996351 | 56.6649865130467 | 249271.313471311 | 1267.08442127893 |
| China | 2037 | 85 to 89 | 49326.4605775204 | 549.236042552429 | 825087.916660713 | 9187.1181673854 | 12194.9201429171 | 135.786950860024 | 183966.887569356 | 2048.41872103283 |
| China | 2037 | 90 to 94 | 14605.7981940488 | 609.688877137971 | 258670.264331111 | 10797.6558975925 | 8691.61206478777 | 362.813393002956 | 95583.5895284308 | 3989.93951567471 |
| China | 2037 | 95 plus | 2579.37127868449 | 667.858907262662 | 43738.9515818006 | 11325.0266255326 | 2945.4507441374 | 762.645351459006 | 27591.8187744411 | 7144.16031858967 |
| China | 2038 | 30 to 34 | 2396.05217295495 | 5.49840214303348 | 4435.65450282686 | 10.1788318716038 | 2.66127036917699 | 0.00610701839727818 | 585.637507378426 | 1.34390668198145 |
| China | 2038 | 35 to 39 | 6932.03847255042 | 16.4549237823422 | 27453.9110921838 | 65.1687113881346 | 2.72608691087093 | 0.00647104780506477 | 2526.57003309146 | 5.99744468959558 |
| China | 2038 | 40 to 44 | 15296.1363294228 | 33.7197178137631 | 83141.1432127221 | 183.281308918123 | 21.4329672403051 | 0.0472481149285094 | 8010.94250012375 | 17.6598007960255 |
| China | 2038 | 45 to 49 | 33246.5678843604 | 57.2639960236606 | 232881.710305619 | 401.116210831421 | 91.1656246295697 | 0.157023966637411 | 22986.9467652912 | 39.5927914346597 |
| China | 2038 | 50 to 54 | 49108.5146041839 | 85.6971802195431 | 423342.068328968 | 738.756238434685 | 226.315406921291 | 0.394933391280801 | 42675.9046723928 | 74.4719062105476 |
| China | 2038 | 55 to 59 | 57989.6071149045 | 118.91592870874 | 601910.574806305 | 1234.30315471661 | 394.858508815771 | 0.809713474887593 | 61245.494113411 | 125.592587604375 |
| China | 2038 | 60 to 64 | 72549.7262973728 | 156.910715254452 | 873105.569438145 | 1888.35473798536 | 670.447250922508 | 1.45004485959641 | 87699.529079706 | 189.676743630576 |
| China | 2038 | 65 to 69 | 107808.551298565 | 199.345457836834 | 1480552.82536126 | 2737.64443792491 | 1376.86351158793 | 2.54591573479366 | 146729.138411917 | 271.312311708218 |
| China | 2038 | 70 to 74 | 126797.678470991 | 277.215152435688 | 1755027.84348706 | 3836.98122101225 | 3209.15600113807 | 7.01611165735123 | 197597.43186939 | 432.003194830617 |
| China | 2038 | 75 to 79 | 99324.3136423055 | 381.682530637053 | 1397070.82481964 | 5368.6505181067 | 4541.96361388717 | 17.453814706971 | 178735.333163023 | 686.842443448432 |
| China | 2038 | 80 to 84 | 95164.6979976635 | 474.502948283246 | 1455112.66340623 | 7255.37161781878 | 11296.8206775298 | 56.3273443882168 | 253789.485850774 | 1265.42574939331 |
| China | 2038 | 85 to 89 | 54154.4269157867 | 553.287011372431 | 903757.139193204 | 9233.54035909738 | 13234.0120249374 | 135.209758070764 | 200489.20817955 | 2048.36577771542 |
| China | 2038 | 90 to 94 | 16086.8782772705 | 612.718150461227 | 284981.546629574 | 10854.3971774292 | 9517.58972402659 | 362.506626334952 | 104903.699569518 | 3995.57948216671 |
| China | 2038 | 95 plus | 2734.72278672365 | 669.523619741478 | 46467.1473578699 | 11376.2363224297 | 3102.10233380164 | 759.466660905563 | 29142.7326358159 | 7134.81744409851 |
| China | 2039 | 30 to 34 | 2452.24946256652 | 5.50876505998983 | 4547.48952663502 | 10.2155395678131 | 2.72775055738652 | 0.00612765429956375 | 601.126407172513 | 1.3503781727832 |
| China | 2039 | 35 to 39 | 6929.18407813597 | 16.4798423945228 | 27469.3684505705 | 65.3310487407715 | 2.72993250655879 | 0.00649266305360963 | 2530.72802928786 | 6.01889033337451 |
| China | 2039 | 40 to 44 | 14897.6138189299 | 33.7639664839409 | 81032.784021482 | 183.652780710775 | 20.8940521499829 | 0.0473543001637836 | 7814.47092491982 | 17.7107245231086 |
| China | 2039 | 45 to 49 | 31523.2615659225 | 57.3315992426656 | 220932.452585468 | 401.811557628351 | 86.4909011500063 | 0.157301669831957 | 21823.1810235058 | 39.6899878530435 |
| China | 2039 | 50 to 54 | 51122.469841406 | 85.846518779347 | 439466.921617102 | 737.967188528675 | 234.460653255373 | 0.393713976166371 | 44143.3149672311 | 74.1268942809906 |
| China | 2039 | 55 to 59 | 59474.2699905735 | 119.442938190907 | 615212.335757232 | 1235.53881377243 | 400.986622661698 | 0.805306570279096 | 62394.0103410349 | 125.306690133872 |
| China | 2039 | 60 to 64 | 69900.1487539981 | 157.777863898572 | 838320.43295443 | 1892.24786401489 | 642.126750496525 | 1.44940159429458 | 83992.3933039319 | 189.586726715873 |
| China | 2039 | 65 to 69 | 106246.065199095 | 200.246171875317 | 1456103.86984028 | 2744.37669989905 | 1331.34177122459 | 2.50923262565867 | 143546.991803927 | 270.548707277667 |
| China | 2039 | 70 to 74 | 128963.659562082 | 278.476675534758 | 1781242.4842371 | 3846.31210851173 | 3241.61097477065 | 6.99974745363487 | 199884.241911099 | 431.618483596091 |
| China | 2039 | 75 to 79 | 113893.522793513 | 384.244612066347 | 1596643.36881912 | 5386.62425055091 | 5143.85972262268 | 17.3539314191399 | 203371.499662587 | 686.11806076871 |
| China | 2039 | 80 to 84 | 94083.7534572743 | 477.877066342336 | 1434134.79036313 | 7284.36208350725 | 11023.1725163414 | 55.9897022633868 | 248808.655032793 | 1263.76707750769 |
| China | 2039 | 85 to 89 | 58557.8016979794 | 557.337980192433 | 975017.361327759 | 9279.96255080935 | 14145.4330048036 | 134.632565281505 | 215209.982156055 | 2048.31283439802 |
| China | 2039 | 90 to 94 | 17721.5495176005 | 615.747423784483 | 314028.565926266 | 10911.138457266 | 10424.3111711376 | 362.199859666948 | 115157.296403094 | 4001.21944865871 |
| China | 2039 | 95 plus | 2922.11237068756 | 671.188332220295 | 49750.9860875821 | 11427.4460193268 | 3292.6055592438 | 756.287970352119 | 31021.7511052254 | 7125.47456960736 |
| China | 2040 | 30 to 34 | 2503.59603634098 | 5.51920654587614 | 4650.81811928061 | 10.2527825715558 | 2.78908576928246 | 0.00614857197862139 | 615.545018678975 | 1.35697614433823 |
| China | 2040 | 35 to 39 | 6927.15722282447 | 16.5049126383482 | 27488.4382542623 | 65.495015827898 | 2.73418295790285 | 0.00651457003296464 | 2535.27575747289 | 6.04064604645286 |
| China | 2040 | 40 to 44 | 14635.2414926311 | 33.808448333403 | 79663.0384855617 | 184.027282506891 | 20.5454483867943 | 0.0474614464422245 | 7689.04506628465 | 17.7622407520627 |
| China | 2040 | 45 to 49 | 29373.8832246988 | 57.3995228334483 | 205983.126589114 | 402.511751255801 | 80.6413692447383 | 0.157581348025235 | 20361.3599967615 | 39.7881457863035 |
| China | 2040 | 50 to 54 | 53021.3619387225 | 85.9403190105079 | 455995.278242711 | 739.1054897 | 243.263309374986 | 0.394296292037909 | 45832.765048164 | 74.2885943579701 |
| China | 2040 | 55 to 59 | 60965.2505881334 | 119.638758544198 | 628998.306792323 | 1234.35195992918 | 409.277399318966 | 0.803169666036772 | 63590.5777487414 | 124.790724282672 |
| China | 2040 | 60 to 64 | 69139.3415135723 | 158.447826989212 | 826451.115760028 | 1893.99234268496 | 629.437275071399 | 1.44249231013425 | 82554.6872582857 | 189.192007292716 |
| China | 2040 | 65 to 69 | 102944.787095972 | 201.318524358022 | 1406046.44699838 | 2749.66031669639 | 1282.59367493026 | 2.50823643694723 | 138284.767655874 | 270.42928691201 |
| China | 2040 | 70 to 74 | 132438.918010441 | 279.670114137425 | 1825653.23312851 | 3855.21609323444 | 3277.27237671575 | 6.92058764466248 | 203874.852725021 | 430.520757704033 |
| China | 2040 | 75 to 79 | 128221.383333899 | 385.905898707262 | 1793861.29168645 | 5398.95636690899 | 5754.88425464082 | 17.3203854341532 | 227785.525237796 | 685.562544591247 |
| China | 2040 | 80 to 84 | 90400.5886492224 | 481.00926926081 | 1373375.49680353 | 7307.54471855807 | 10475.7215540193 | 55.739893345505 | 237290.571318369 | 1262.5909412517 |
| China | 2040 | 85 to 89 | 62843.285611442 | 561.263129793279 | 1043057.1242811 | 9315.70493858134 | 14999.8637440085 | 133.966109338849 | 229072.736006878 | 2045.88413082782 |
| China | 2040 | 90 to 94 | 19807.9351275586 | 620.264669490931 | 350164.801700799 | 10965.0427263427 | 11526.6667489498 | 360.94545419943 | 127774.654194562 | 4001.12899920813 |
| China | 2040 | 95 plus | 3166.60182461063 | 674.498498239657 | 53931.2705221865 | 11487.5702693831 | 3547.99566839164 | 755.736869565289 | 33494.5601775963 | 7134.47152193328 |
| China | 2041 | 30 to 34 | 2543.95151506117 | 5.52964803176246 | 4733.99500328689 | 10.2900255752984 | 2.83831492920615 | 0.00616948965767902 | 627.321380691786 | 1.36357411589325 |
| China | 2041 | 35 to 39 | 6952.04895252044 | 16.5299828821735 | 27614.3337020767 | 65.6589829150245 | 2.74905960221392 | 0.00653647701231965 | 2549.67679655367 | 6.06240175953121 |
| China | 2041 | 40 to 44 | 14459.2232547184 | 33.8529301828651 | 78761.4706733775 | 184.401784303008 | 20.3174407162253 | 0.0475685927206654 | 7608.59068336109 | 17.8137569810168 |
| China | 2041 | 45 to 49 | 27641.8405807061 | 57.4674464242309 | 193944.937424605 | 403.211944883251 | 75.9311504538846 | 0.157861026218514 | 19185.311291384 | 39.8863037195636 |
| China | 2041 | 50 to 54 | 53408.2364532897 | 86.0341192416688 | 459528.33320972 | 740.243790840526 | 245.132631652015 | 0.394878607909446 | 46217.2329332456 | 74.4502944349497 |
| China | 2041 | 55 to 59 | 62879.5295366891 | 119.834578897489 | 647065.666897999 | 1233.16510608593 | 420.317438159453 | 0.801032761794448 | 65209.3779220542 | 124.274758431471 |
| China | 2041 | 60 to 64 | 70068.1219180872 | 159.117790079852 | 834794.894126236 | 1895.73682135502 | 632.164426347287 | 1.43558302597393 | 83137.6012550813 | 188.797287869559 |
| China | 2041 | 65 to 69 | 98489.3185799363 | 202.390876840726 | 1340636.27259866 | 2754.94393349372 | 1220.09641649639 | 2.50724024823578 | 131540.684921953 | 270.309866546353 |
| China | 2041 | 70 to 74 | 135797.157009945 | 280.863552740092 | 1868296.95705377 | 3864.12007795714 | 3307.82132787141 | 6.84142783569008 | 207625.469042688 | 429.423031811975 |
| China | 2041 | 75 to 79 | 142544.741968089 | 387.567185348177 | 1990237.43423799 | 5411.28848326706 | 6357.98943223617 | 17.2868394491666 | 251941.221555819 | 685.007028413785 |
| China | 2041 | 80 to 84 | 86589.3462074229 | 484.141472179284 | 1311110.33706033 | 7330.7273536089 | 9924.4754016927 | 55.4900844276232 | 225605.715555172 | 1261.4148049957 |
| China | 2041 | 85 to 89 | 66253.0091565896 | 565.188279394125 | 1096203.77479238 | 9351.44732635333 | 15625.7719400967 | 133.299653396194 | 239539.771203878 | 2043.45542725763 |
| China | 2041 | 90 to 94 | 22090.1830063251 | 624.781915197378 | 389592.831906655 | 11018.9469954194 | 12717.4633243262 | 359.691048731912 | 141463.239619514 | 4001.03854975755 |
| China | 2041 | 95 plus | 3485.68528064515 | 677.80866425902 | 59384.9428817789 | 11547.6945194394 | 3883.60323080473 | 755.185768778459 | 36735.8581063559 | 7143.4684742592 |
| China | 2042 | 30 to 34 | 2593.15413259747 | 5.54008951764877 | 4833.89286560664 | 10.3272685790411 | 2.8975489144322 | 0.00619040733673666 | 641.337545109587 | 1.37017208744828 |
| China | 2042 | 35 to 39 | 7047.30583120915 | 16.5550531259988 | 28020.1130039902 | 65.822950002151 | 2.79183264445423 | 0.00655838399167466 | 2589.95957961501 | 6.08415747260956 |
| China | 2042 | 40 to 44 | 14300.9757725773 | 33.8974120323271 | 77955.2488647311 | 184.776286099125 | 20.1139127587591 | 0.0476757389991063 | 7537.1783280273 | 17.8652732099709 |
| China | 2042 | 45 to 49 | 26680.6918731708 | 57.5353700150136 | 187304.875394481 | 403.912138510701 | 73.3340796438121 | 0.158140704411792 | 18541.8656522974 | 39.9844616528236 |
| China | 2042 | 50 to 54 | 51679.0426863558 | 86.1279194728297 | 444848.975961518 | 741.382091996451 | 237.287073529162 | 0.395460923780983 | 44769.1813862115 | 74.6119945119292 |
| China | 2042 | 55 to 59 | 65012.1755741677 | 120.030399250781 | 667277.514182198 | 1231.97825224269 | 432.706698310146 | 0.798895857552124 | 67031.5886833414 | 123.758792580271 |
| China | 2042 | 60 to 64 | 72787.6757854329 | 159.787753170492 | 864354.438526853 | 1897.48130002509 | 650.79982075556 | 1.4286737418136 | 85822.5038973263 | 188.402568446402 |
| China | 2042 | 65 to 69 | 93919.0539284773 | 203.463229323431 | 1274126.83369221 | 2760.22755029106 | 1156.88752098896 | 2.50624405952433 | 124720.477356946 | 270.190446180696 |
| China | 2042 | 70 to 74 | 138867.422953399 | 282.056991342758 | 1906837.57938576 | 3873.02406267984 | 3329.3226653237 | 6.76226802671769 | 210880.897281302 | 428.325305919917 |
| China | 2042 | 75 to 79 | 151173.213015933 | 389.228471989093 | 2106490.68922099 | 5423.62059962514 | 6701.04063754096 | 17.2532934641799 | 265835.471206944 | 684.451512236322 |
| China | 2042 | 80 to 84 | 87839.2027885347 | 487.273675097758 | 1325664.86513543 | 7353.90998865973 | 9957.98051602363 | 55.2402755097414 | 227179.028218941 | 1260.23866873971 |
| China | 2042 | 85 to 89 | 69411.316107137 | 569.113428994971 | 1144898.6430622 | 9387.18971412532 | 16176.4673362315 | 132.633197453538 | 248931.660866211 | 2041.02672368743 |
| China | 2042 | 90 to 94 | 24685.4049109254 | 629.299160903826 | 434352.743439161 | 11072.8512644961 | 14060.3296867366 | 358.436643264394 | 156944.470960083 | 4000.94810030697 |
| China | 2042 | 95 plus | 3890.17614319347 | 681.118830278383 | 66297.4765110365 | 11607.8187694957 | 4310.05817450079 | 754.634667991629 | 40850.9486606696 | 7152.46542658512 |
| China | 2043 | 30 to 34 | 2635.13962682311 | 5.55053100353509 | 4920.59861787384 | 10.3645115827838 | 2.94885456432416 | 0.00621132501579429 | 653.627794744788 | 1.37677005900331 |
| China | 2043 | 35 to 39 | 7188.55372778228 | 16.5801233698241 | 28609.5880136999 | 65.9869170892775 | 2.85298089372354 | 0.00658029097102966 | 2647.30750269184 | 6.10591318568791 |
| China | 2043 | 40 to 44 | 14212.7339461765 | 33.9418938817892 | 77529.5243525551 | 185.150787895241 | 20.0084720668702 | 0.0477828852775472 | 7502.42642181292 | 17.916789438925 |
| China | 2043 | 45 to 49 | 25904.1256742119 | 57.6032936057962 | 181953.635720371 | 404.612332138151 | 71.241438526799 | 0.15842038260507 | 18025.1015196302 | 40.0826195860837 |
| China | 2043 | 50 to 54 | 49378.1278317122 | 86.2217197039906 | 425232.377834773 | 742.520393152376 | 226.809135581907 | 0.39604323965252 | 42821.983399731 | 74.7736945889088 |
| China | 2043 | 55 to 59 | 67478.4216923049 | 120.226219604072 | 690796.577235519 | 1230.79139839944 | 447.19061129605 | 0.7967589533098 | 69171.5289723227 | 123.242826729071 |
| China | 2043 | 60 to 64 | 75690.1816553696 | 160.457716261132 | 895891.749824303 | 1899.22577869515 | 670.666469512704 | 1.42176445765328 | 88685.9702156423 | 188.007849023245 |
| China | 2043 | 65 to 69 | 89350.6448560507 | 204.535581806135 | 1208103.76343304 | 2765.5111670884 | 1094.40866378715 | 2.50524787081289 | 117979.571575868 | 270.071025815039 |
| China | 2043 | 70 to 74 | 139928.176671781 | 283.250429945425 | 1917706.22819087 | 3881.92804740254 | 3301.51360260767 | 6.68310821774529 | 211054.141413709 | 427.227580027859 |
| China | 2043 | 75 to 79 | 153752.812193383 | 390.889758630008 | 2138180.90287647 | 5435.95271598322 | 6773.22580531436 | 17.2197474791933 | 269004.061427389 | 683.895996058859 |
| China | 2043 | 80 to 84 | 98931.5001632563 | 490.405878016232 | 1488209.81318421 | 7377.09262371056 | 11093.4423882049 | 54.9904665918596 | 253995.620203068 | 1259.06253248372 |
| China | 2043 | 85 to 89 | 71751.266390439 | 573.038578595817 | 1179863.51473752 | 9422.93210189731 | 16523.8104003887 | 131.966741510883 | 255256.792593058 | 2038.59802011724 |
| China | 2043 | 90 to 94 | 27365.7015389536 | 633.816406610273 | 480409.575790427 | 11126.7555335728 | 15421.7253018746 | 357.182237796876 | 172741.309993411 | 4000.85765085639 |
| China | 2043 | 95 plus | 4355.07645776226 | 684.428996297746 | 74244.0548688512 | 11667.943019552 | 4798.29406480951 | 754.083567204799 | 45568.9580340013 | 7161.46237891105 |
| China | 2044 | 30 to 34 | 2656.78274534187 | 5.5609724894214 | 4969.49088659122 | 10.4017545865265 | 2.97748548261354 | 0.00623224269485192 | 660.911076441481 | 1.38336803055834 |
| China | 2044 | 35 to 39 | 7355.3223193701 | 16.6051936136495 | 29301.7405366968 | 66.150884176404 | 2.9244641809805 | 0.00660219795038467 | 2714.27005703548 | 6.12766889876626 |
| China | 2044 | 40 to 44 | 14205.582505315 | 33.9863757312513 | 77545.6268233288 | 185.525289691358 | 20.0170150483322 | 0.0478900315559881 | 7510.36976299497 | 17.9683056678791 |
| China | 2044 | 45 to 49 | 25230.1794196596 | 57.6712171965789 | 177317.355922013 | 405.312525765601 | 69.4285850462464 | 0.158700060798349 | 17578.4086987262 | 40.1807775193437 |
| China | 2044 | 50 to 54 | 46826.0047021766 | 86.3155199351515 | 403433.421274147 | 743.658694308301 | 215.16860631699 | 0.396625555524057 | 40652.3084796406 | 74.9353946658883 |
| China | 2044 | 55 to 59 | 70269.0601275506 | 120.422039957363 | 717502.840054197 | 1229.60454455619 | 463.680440593513 | 0.794622049067476 | 71613.9767217529 | 122.726860877871 |
| China | 2044 | 60 to 64 | 77658.3563588324 | 161.127679351772 | 916206.490827153 | 1900.97025736522 | 681.914663584119 | 1.41485517349295 | 90423.491076732 | 187.613129600088 |
| China | 2044 | 65 to 69 | 86204.9219730631 | 205.60793428884 | 1161706.86201581 | 2770.79478388574 | 1049.95374620708 | 2.50425168210144 | 113182.19389129 | 269.951605449381 |
| China | 2044 | 70 to 74 | 138113.623303007 | 284.443868548092 | 1889219.52286471 | 3890.83203212525 | 3206.59133029453 | 6.6039484087729 | 206910.201484362 | 426.129854135801 |
| China | 2044 | 75 to 79 | 156970.99593177 | 392.551045270923 | 2178627.99387612 | 5448.28483234129 | 6872.31685491452 | 17.1862014942066 | 273250.159386141 | 683.340479881396 |
| China | 2044 | 80 to 84 | 114360.778540244 | 493.538080934706 | 1714763.80991971 | 7400.27525876138 | 12684.2982765281 | 54.7406576739778 | 291472.680923311 | 1257.88639622773 |
| China | 2044 | 85 to 89 | 71156.1461581869 | 576.963728196663 | 1166525.36642686 | 9458.6744896693 | 16193.0843377382 | 131.300285568227 | 251117.972257783 | 2036.16931654704 |
| China | 2044 | 90 to 94 | 29804.1876101956 | 638.333652316721 | 522031.826388185 | 11180.6598026495 | 16618.4876074354 | 355.927832329358 | 186798.26110163 | 4000.76720140581 |
| China | 2044 | 95 plus | 4867.87281001348 | 687.739162317109 | 83012.1983796695 | 11728.0672696084 | 5333.5630799037 | 753.532466417969 | 50753.0847832418 | 7170.45933123697 |
| China | 2045 | 30 to 34 | 2663.35260820073 | 5.57141397530772 | 4990.2469251549 | 10.4389975902691 | 2.98925390666731 | 0.00625316037390956 | 664.457818687671 | 1.38996600211337 |
| China | 2045 | 35 to 39 | 7507.29823596503 | 16.6302638574748 | 29936.1074590059 | 66.3148512635305 | 2.99027914891021 | 0.00662410492973968 | 2775.99711804639 | 6.14942461184461 |
| China | 2045 | 40 to 44 | 14200.0886123275 | 34.0308575807134 | 77570.5844577784 | 185.899791487475 | 20.0278284722629 | 0.0479971778344291 | 7519.14838192061 | 18.0198218968332 |
| China | 2045 | 45 to 49 | 24786.9275538403 | 57.7391407873615 | 174297.845868467 | 406.012719393051 | 68.2486649295006 | 0.158979738991627 | 17291.4082439557 | 40.2789354526038 |
| China | 2045 | 50 to 54 | 43643.5831376866 | 86.4093201663124 | 376181.753654333 | 744.796995464226 | 200.62158485718 | 0.397207871395594 | 37930.0090719479 | 75.0970947428678 |
| China | 2045 | 55 to 59 | 72950.5238285489 | 120.617860310655 | 742955.593698668 | 1228.41769071294 | 479.300547136563 | 0.792485144825152 | 73914.0023441776 | 122.21089502667 |
| China | 2045 | 60 to 64 | 79863.2774781402 | 161.797642442412 | 939179.53705548 | 1902.71473603528 | 694.961753067571 | 1.40794588933263 | 92410.9623309148 | 187.218410176931 |
| China | 2045 | 65 to 69 | 85491.3474943394 | 206.680286771544 | 1148298.59650167 | 2776.07840068308 | 1035.44797907636 | 2.50325549338999 | 111613.533445733 | 269.832185083724 |
| China | 2045 | 70 to 74 | 133903.676140239 | 285.637307150759 | 1828154.01055726 | 3899.73601684795 | 3058.75023212594 | 6.5247885998005 | 199250.458622718 | 425.032128243743 |
| China | 2045 | 75 to 79 | 161763.772862684 | 394.212331911839 | 2240746.74553072 | 5460.61694869937 | 7038.53747123733 | 17.15265551 | 280178.631771875 | 682.784963703934 |
| China | 2045 | 80 to 84 | 129329.867526311 | 496.67028385318 | 1933022.48434434 | 7423.45789381221 | 14189.0797177336 | 54.490848756096 | 327239.57266013 | 1256.71025997173 |
| China | 2045 | 85 to 89 | 68647.2227737077 | 580.88887779751 | 1122013.82295937 | 9494.41687744129 | 15437.8056575826 | 130.633829625572 | 240339.676415056 | 2033.74061297684 |
| China | 2045 | 90 to 94 | 32275.030042732 | 642.850898023168 | 564043.534895853 | 11234.5640717262 | 17806.7659896339 | 354.67342686184 | 200858.337069572 | 4000.67675195523 |
| China | 2045 | 95 plus | 5519.95691439279 | 691.049328336472 | 94161.5983388622 | 11788.1915196647 | 6014.65702257475 | 752.981365631139 | 57347.9891072808 | 7179.45628356289 |
| United States of America | 2022 | 30 to 34 | 1187.42520505744 | 9.93159028499177 | 2069.07158979599 | 17.3056553058231 | 4.25924423548424 | 0.0356241963623269 | 431.882029232719 | 3.61224887893694 |
| United States of America | 2022 | 35 to 39 | 3310.40218657585 | 28.9304411992129 | 13048.9202931326 | 114.037811715008 | 6.96680626739818 | 0.0608846803819174 | 1559.63861793587 | 13.6300759802502 |
| United States of America | 2022 | 40 to 44 | 5103.42481382487 | 46.3876484222093 | 33558.3234817112 | 305.028832224863 | 28.3350855296816 | 0.25755214067296 | 4142.56643091969 | 37.6539132393189 |
| United States of America | 2022 | 45 to 49 | 6270.89344588546 | 62.1129790586803 | 58270.9775021297 | 577.171982996044 | 67.7052304601294 | 0.670617926779225 | 7634.03204898626 | 75.6148189861364 |
| United States of America | 2022 | 50 to 54 | 12563.276513914 | 117.773139352509 | 106471.485648586 | 998.105956075386 | 148.882102998064 | 1.39567991232749 | 14299.5546470807 | 134.049699556025 |
| United States of America | 2022 | 55 to 59 | 22435.317287921 | 213.903556344661 | 191870.945420345 | 1829.34241838072 | 285.946278571885 | 2.72627862245469 | 25067.1792870426 | 238.996343497868 |
| United States of America | 2022 | 60 to 64 | 39313.7133033992 | 378.226025836615 | 337881.141949357 | 3250.6581237543 | 556.322479910625 | 5.35221995023229 | 42914.8804935336 | 412.87182854129 |
| United States of America | 2022 | 65 to 69 | 54366.4188996557 | 602.561502835016 | 513200.431475012 | 5687.97484741302 | 925.73676815457 | 10.2602553108824 | 62333.1970346333 | 690.860013256044 |
| United States of America | 2022 | 70 to 74 | 55706.7653085755 | 803.058188967285 | 636201.581169456 | 9171.36162478666 | 1451.83781137584 | 20.9294192010505 | 76955.3139084382 | 1109.37324535707 |
| United States of America | 2022 | 75 to 79 | 47626.6024786985 | 977.074800661876 | 659002.357675278 | 13519.6416235919 | 2030.38915834818 | 41.654075220805 | 80894.3605882466 | 1659.57337145311 |
| United States of America | 2022 | 80 to 84 | 25766.536820292 | 898.732878626571 | 520937.185301703 | 18170.2096558476 | 2888.87317393226 | 100.763456171981 | 73376.8915782177 | 2559.37479889911 |
| United States of America | 2022 | 85 to 89 | 9722.11994335729 | 609.966763016047 | 342111.639124994 | 21464.1179416601 | 3410.36273590955 | 213.966494021154 | 57560.8039803985 | 3611.37051230418 |
| United States of America | 2022 | 90 to 94 | 3122.65981137375 | 428.474176421303 | 167362.904567177 | 22964.6157537572 | 3820.43903338659 | 524.219597163858 | 44269.0085777722 | 6074.34947676989 |
| United States of America | 2022 | 95 plus | 648.630874159231 | 343.190938708588 | 42189.0894591067 | 22322.2695550829 | 1700.34090934177 | 899.651274784004 | 16846.4576857712 | 8913.46967501121 |
| United States of America | 2023 | 30 to 34 | 1205.79772690175 | 10.0077463231692 | 2118.92376404078 | 17.5864085953638 | 4.40605938153802 | 0.0365689232874506 | 446.625399568439 | 3.70685198739741 |
| United States of America | 2023 | 35 to 39 | 3334.70708533431 | 28.8942627099074 | 13195.5261195715 | 114.335378951618 | 7.23101908935207 | 0.0626546679757776 | 1620.55611046125 | 14.0416452760554 |
| United States of America | 2023 | 40 to 44 | 5158.7441254116 | 46.2219758129576 | 34092.3636566829 | 305.465122913035 | 29.4549802431385 | 0.263914501528192 | 4242.7464802892 | 38.0147028860036 |
| United States of America | 2023 | 45 to 49 | 6271.86694482617 | 61.7265873904056 | 58575.7549809095 | 576.492054215335 | 70.2360341656075 | 0.69125042655047 | 7749.28892755375 | 76.2671090455358 |
| United States of America | 2023 | 50 to 54 | 12398.4402981076 | 117.208109875133 | 105348.048130745 | 995.903138100666 | 150.09535378938 | 1.41891982343761 | 14247.2837642494 | 134.686070240401 |
| United States of America | 2023 | 55 to 59 | 22100.9948130135 | 213.850469642036 | 188883.555517579 | 1827.64791344631 | 283.504040177565 | 2.74320104820297 | 24790.6737430339 | 239.875954342502 |
| United States of America | 2023 | 60 to 64 | 39370.8070405066 | 379.010607559808 | 337987.735687099 | 3253.7036113772 | 558.991064119193 | 5.38123444140587 | 43097.3648943585 | 414.885029814907 |
| United States of America | 2023 | 65 to 69 | 55853.1009803687 | 604.865654509185 | 526579.486751108 | 5702.63495337116 | 954.674522207569 | 10.3387246112134 | 64212.5708329438 | 695.395206404698 |
| United States of America | 2023 | 70 to 74 | 57262.6784688764 | 805.967632030865 | 654006.727631528 | 9205.09252615441 | 1501.29439233382 | 21.130598824995 | 79362.6422911643 | 1117.02286007959 |
| United States of America | 2023 | 75 to 79 | 50232.626196902 | 981.186085053466 | 695121.161927283 | 13577.6936852908 | 2140.60196407661 | 41.8120456724125 | 85402.4751602317 | 1668.15328204982 |
| United States of America | 2023 | 80 to 84 | 27426.508131138 | 905.05237733174 | 553541.524550514 | 18266.4183989758 | 3065.26649821907 | 101.151291958266 | 78004.3493980353 | 2574.07984739974 |
| United States of America | 2023 | 85 to 89 | 10014.3668642288 | 615.673515895022 | 351435.049673391 | 21605.8843833679 | 3486.31335524043 | 214.335147696623 | 59031.0047204889 | 3629.1686449893 |
| United States of America | 2023 | 90 to 94 | 3275.70797924375 | 432.702887749262 | 175226.060237836 | 23146.3923995271 | 4001.82580830688 | 528.619032739201 | 46396.3679789245 | 6128.70333154449 |
| United States of America | 2023 | 95 plus | 692.187618397548 | 345.756696419765 | 45090.2349594085 | 22523.157401238 | 1805.19443834842 | 901.718044081229 | 17982.7176526051 | 8982.60079053181 |
| United States of America | 2024 | 30 to 34 | 1216.61273599412 | 10.0839023613467 | 2155.65521425184 | 17.8671618849045 | 4.52598438449687 | 0.0375136502125743 | 458.641755860157 | 3.80145509585788 |
| United States of America | 2024 | 35 to 39 | 3368.29683247391 | 28.8580842206019 | 13379.8829676748 | 114.632946188227 | 7.519603922 | 0.0644246555696378 | 1686.96981023809 | 14.4532145718606 |
| United States of America | 2024 | 40 to 44 | 5189.98026021734 | 46.0563032037059 | 34471.3359025111 | 305.901413601207 | 30.4568860935105 | 0.270276862383424 | 4324.44714114059 | 38.3754925326883 |
| United States of America | 2024 | 45 to 49 | 6318.94124685464 | 61.3401957221309 | 59317.1075998891 | 575.812125434626 | 73.3343989713244 | 0.711882926321716 | 7923.82805378119 | 76.9193991049353 |
| United States of America | 2024 | 50 to 54 | 12169.8158216035 | 116.643080397757 | 103676.530459956 | 993.700320125946 | 150.466005312356 | 1.44215973454773 | 14118.7044869575 | 135.322440924777 |
| United States of America | 2024 | 55 to 59 | 21885.0099249896 | 213.797382939412 | 186910.653060595 | 1825.9534085119 | 282.535402403583 | 2.76012347395124 | 24644.5389610316 | 240.755565187135 |
| United States of America | 2024 | 60 to 64 | 39403.0634588189 | 379.795189283002 | 337881.824305391 | 3256.74909900009 | 561.30353420455 | 5.41024893257945 | 43252.4368896361 | 416.898231088524 |
| United States of America | 2024 | 65 to 69 | 57047.5681097604 | 607.169806183353 | 537177.204760725 | 5717.2950593293 | 978.76339226584 | 10.4171939115444 | 65763.0315835465 | 699.930399553352 |
| United States of America | 2024 | 70 to 74 | 60032.5512138457 | 808.877075094446 | 685679.144143927 | 9238.82342752217 | 1583.18380091172 | 21.3317784489396 | 83469.9857632591 | 1124.67247480211 |
| United States of America | 2024 | 75 to 79 | 52452.3941391708 | 985.297369445057 | 725900.152058111 | 13635.7457469896 | 2234.27758566366 | 41.9700161240201 | 89261.0424165249 | 1676.73319264652 |
| United States of America | 2024 | 80 to 84 | 29014.58998 | 911.371876036909 | 584595.719342634 | 18362.6271421039 | 3232.61693252727 | 101.539127744552 | 82416.9960393165 | 2588.78489590037 |
| United States of America | 2024 | 85 to 89 | 10451.7093278189 | 621.380268773996 | 365798.749025397 | 21747.6508250757 | 3611.35014464878 | 214.703801372091 | 61342.5282455003 | 3646.96677767442 |
| United States of America | 2024 | 90 to 94 | 3361.40654733688 | 436.931599077222 | 179468.503380969 | 23328.169045297 | 4100.62301062213 | 533.018468314544 | 47567.5573763618 | 6183.05718631907 |
| United States of America | 2024 | 95 plus | 757.723250593745 | 348.322454130942 | 49432.7518289167 | 22724.0452473932 | 1966.04829378282 | 903.784813378453 | 19690.6850018311 | 9051.73190605241 |
| United States of America | 2025 | 30 to 34 | 1217.43862503849 | 10.1425732379733 | 2171.39251421287 | 18.0900352188963 | 4.59618990613479 | 0.0382912056343963 | 465.63349058371 | 3.87922781745905 |
| United States of America | 2025 | 35 to 39 | 3427.51246492834 | 28.9938487155822 | 13667.0302849467 | 115.611485743002 | 7.75706918116147 | 0.0656182268091628 | 1735.1050683546 | 14.677517404321 |
| United States of America | 2025 | 40 to 44 | 5210.22391794796 | 45.9311867372298 | 34680.1518792692 | 305.725926011558 | 31.1718762095364 | 0.274798413595614 | 4433.53228992913 | 39.0841934475775 |
| United States of America | 2025 | 45 to 49 | 6426.33670627399 | 61.1722535661145 | 60451.5510571467 | 575.437886116737 | 75.9406711959174 | 0.722878710952424 | 8119.49882272937 | 77.2894517538827 |
| United States of America | 2025 | 50 to 54 | 11883.1553371055 | 116.176513421575 | 101466.548745972 | 991.994931297319 | 150.431162365813 | 1.47070095928511 | 13906.9989500467 | 135.962764462816 |
| United States of America | 2025 | 55 to 59 | 21827.7558821388 | 213.017538055927 | 186694.544569847 | 1821.95606673811 | 285.78785225481 | 2.78900978288041 | 24721.8898454415 | 241.261453509061 |
| United States of America | 2025 | 60 to 64 | 39081.7735455156 | 379.467815157027 | 334999.020578059 | 3252.70157636146 | 559.235040265948 | 5.42994034397709 | 43004.5003916025 | 417.555866202327 |
| United States of America | 2025 | 65 to 69 | 57879.7913436459 | 607.649219928509 | 544604.39339564 | 5717.51257449598 | 997.462530198269 | 10.4718298790038 | 66866.2077286346 | 701.992837614868 |
| United States of America | 2025 | 70 to 74 | 63599.9519991199 | 810.77898325587 | 725600.31604661 | 9250.03035383658 | 1682.72911200861 | 21.4516105066914 | 88572.2527665221 | 1129.1285415391 |
| United States of America | 2025 | 75 to 79 | 54220.4209474959 | 988.08621737028 | 749920.412680359 | 13666.1798441518 | 2316.40412315871 | 42.2130066118292 | 92400.7502145332 | 1683.86571270009 |
| United States of America | 2025 | 80 to 84 | 30073.7113862407 | 914.507072072653 | 605621.558955742 | 18416.2570276606 | 3342.974202 | 101.656011451944 | 85366.9842584322 | 2595.91208491709 |
| United States of America | 2025 | 85 to 89 | 11026.5317160643 | 624.320234681466 | 385498.614391013 | 21826.8619365911 | 3799.69193428362 | 215.13787120144 | 64625.5870174901 | 3659.08906736343 |
| United States of America | 2025 | 90 to 94 | 3363.8264329819 | 440.153149924356 | 179047.887250888 | 23428.2276838281 | 4079.24879243224 | 533.765413015837 | 47400.6936293354 | 6202.33089465814 |
| United States of America | 2025 | 95 plus | 834.333363050743 | 352.174616857419 | 54133.3374543018 | 22849.8442247031 | 2151.45435451511 | 908.135340791235 | 21561.1753257156 | 9101.03682245738 |
| United States of America | 2026 | 30 to 34 | 1213.62273196256 | 10.2012441145998 | 2178.65211913992 | 18.312908552888 | 4.6479366628999 | 0.0390687610562183 | 470.756875400975 | 3.95700053906022 |
| United States of America | 2026 | 35 to 39 | 3487.44273576515 | 29.1296132105625 | 13958.3397090892 | 116.590025297776 | 7.99881268879386 | 0.0668117980486878 | 1784.06916558705 | 14.9018202367815 |
| United States of America | 2026 | 40 to 44 | 5228.77712128832 | 45.8060702707537 | 34878.6772664967 | 305.550438421908 | 31.88446057 | 0.279319964807805 | 4542.37122727282 | 39.7928943624667 |
| United States of America | 2026 | 45 to 49 | 6555.8417466347 | 61.0043114100981 | 61799.3413172405 | 575.063646798848 | 78.8659841202304 | 0.733874495583133 | 8345.69572574111 | 77.6595044028301 |
| United States of America | 2026 | 50 to 54 | 11596.4357904642 | 115.709946445393 | 99246.6892085716 | 990.289542468691 | 150.253856781249 | 1.49924218402248 | 13690.3437210443 | 136.603088000856 |
| United States of America | 2026 | 55 to 59 | 21920.6814960529 | 212.237693172441 | 187765.394484074 | 1817.95872496432 | 291.042565503868 | 2.81789609180959 | 24970.6110974285 | 241.767341830986 |
| United States of America | 2026 | 60 to 64 | 38471.7900230972 | 379.140441031053 | 329644.435377632 | 3248.65405372282 | 552.980019820169 | 5.44963175537472 | 42436.5756491291 | 418.213501316129 |
| United States of America | 2026 | 65 to 69 | 58580.3745028597 | 608.128633673665 | 550782.764388708 | 5717.73008966266 | 1014.00308640668 | 10.5264658464631 | 67820.8843262083 | 704.055275676384 |
| United States of America | 2026 | 70 to 74 | 65505.7369122453 | 812.680891417295 | 746497.40034793 | 9261.23728015098 | 1738.75534218567 | 21.5714425644433 | 91372.0205577896 | 1133.58460827608 |
| United States of America | 2026 | 75 to 79 | 56746.5232019148 | 990.875065295504 | 784392.753466506 | 13696.613941314 | 2431.41674349889 | 42.4559970996383 | 96841.9468913928 | 1690.99823275366 |
| United States of America | 2026 | 80 to 84 | 32295.4010431574 | 917.642268108396 | 650027.168336206 | 18469.8869132172 | 3581.78407721919 | 101.772895159336 | 91611.0778725066 | 2603.03927393382 |
| United States of America | 2026 | 85 to 89 | 11509.7730534625 | 627.260200588935 | 401960.668060159 | 21906.0730481065 | 3955.58990611743 | 215.571941030789 | 67364.0851297354 | 3671.21135705245 |
| United States of America | 2026 | 90 to 94 | 3468.40500671316 | 443.374700771489 | 184055.666545372 | 23528.2863223592 | 4181.35120120811 | 534.512357717131 | 48669.9951920504 | 6221.60460299721 |
| United States of America | 2026 | 95 plus | 895.079806016282 | 356.026779583896 | 57762.6050613168 | 22975.643202013 | 2294.06247153436 | 912.485868204017 | 23004.6911588391 | 9150.34173886236 |
| United States of America | 2027 | 30 to 34 | 1209.86610462515 | 10.2599149912264 | 2185.76998414097 | 18.5357818868797 | 4.69874338551271 | 0.0398463164780404 | 475.787120273071 | 4.03477326066139 |
| United States of America | 2027 | 35 to 39 | 3544.97285426648 | 29.2653777055428 | 14241.3118706623 | 117.568564852551 | 8.23762435245872 | 0.0680053692882129 | 1832.25708583973 | 15.126123069242 |
| United States of America | 2027 | 40 to 44 | 5251.59436767657 | 45.6809538042776 | 35106.652516306 | 305.374950832259 | 32.6311160933729 | 0.283841516019995 | 4656.16262199326 | 40.5015952773559 |
| United States of America | 2027 | 45 to 49 | 6668.05177282842 | 60.8363692540817 | 62989.6025907565 | 574.689407480959 | 81.6425051890135 | 0.744870280213841 | 8552.53416026653 | 78.0295570517776 |
| United States of America | 2027 | 50 to 54 | 11469.4682690882 | 115.243379469211 | 98387.7306767751 | 988.584153640063 | 152.050932639407 | 1.52778340875986 | 13658.9968207876 | 137.243411538895 |
| United States of America | 2027 | 55 to 59 | 21888.2852299066 | 211.457848288956 | 187765.573482305 | 1813.96138319052 | 294.674260988886 | 2.84678240073876 | 25078.0266992563 | 242.273230152912 |
| United States of America | 2027 | 60 to 64 | 37837.3643747468 | 378.813066905078 | 324084.278750812 | 3244.60653108418 | 546.29787518989 | 5.46932316677235 | 41838.5245911673 | 418.871136429932 |
| United States of America | 2027 | 65 to 69 | 58973.2190549735 | 608.60804741882 | 554060.660345509 | 5717.94760482935 | 1025.2931057384 | 10.5811018139224 | 68421.7614069862 | 706.1177137 |
| United States of America | 2027 | 70 to 74 | 66573.343294546 | 814.582799578719 | 757808.30586626 | 9272.44420646539 | 1772.76106574618 | 21.6912746221952 | 93008.5592035394 | 1138.04067501307 |
| United States of America | 2027 | 75 to 79 | 59198.2231948641 | 993.663913220728 | 817798.495825846 | 13727.0480384762 | 2543.82207481349 | 42.6989875874474 | 101167.328290018 | 1698.13075280723 |
| United States of America | 2027 | 80 to 84 | 35234.1297552779 | 920.77746414414 | 708814.040120664 | 18523.5167987738 | 3898.87657889602 | 101.889778866728 | 99879.6641096741 | 2610.16646295054 |
| United States of America | 2027 | 85 to 89 | 12053.5612965035 | 630.200166496405 | 420502.856596057 | 21985.2841596219 | 4131.45192707707 | 216.006010860137 | 70449.5019944195 | 3683.33364674147 |
| United States of America | 2027 | 90 to 94 | 3614.81258407636 | 446.596251618623 | 191251.132081741 | 23628.3449608903 | 4332.46373007707 | 535.259302418425 | 50514.5427749194 | 6240.87831133628 |
| United States of America | 2027 | 95 plus | 941.454109452206 | 359.878942310374 | 60434.0657843741 | 23101.4421793229 | 2398.47151602542 | 916.836395616799 | 24066.551639579 | 9199.64665526734 |
| United States of America | 2028 | 30 to 34 | 1210.30893308496 | 10.318585867853 | 2200.27901858276 | 18.7586552208715 | 4.7649392742935 | 0.0406238718998624 | 482.377256321723 | 4.11254598226256 |
| United States of America | 2028 | 35 to 39 | 3589.24175190954 | 29.4011422005231 | 14472.0301614399 | 118.547104407326 | 8.44768971341675 | 0.0691989405277379 | 1873.95405186006 | 15.3504259017025 |
| United States of America | 2028 | 40 to 44 | 5283.19883438927 | 45.5558373378014 | 35394.5738392041 | 305.19946324261 | 33.442024331267 | 0.288363067232185 | 4779.23799738928 | 41.2102961922451 |
| United States of America | 2028 | 45 to 49 | 6747.34589614302 | 60.6684270980652 | 63873.4722219518 | 574.31516816307 | 84.0649747259607 | 0.755866064844549 | 8719.3505761792 | 78.399609700725 |
| United States of America | 2028 | 50 to 54 | 11502.9218181382 | 114.776812493029 | 98904.9009903132 | 986.878764811435 | 155.974714699876 | 1.55632463349724 | 13818.6955198742 | 137.883735076935 |
| United States of America | 2028 | 55 to 59 | 21646.1114598951 | 210.678003405471 | 185964.755435362 | 1809.96404141673 | 295.460581574831 | 2.87566870966793 | 24944.3405276972 | 242.779118474837 |
| United States of America | 2028 | 60 to 64 | 37319.7755619578 | 378.485692779103 | 319528.418637085 | 3240.55900844554 | 541.2325909 | 5.48901457816999 | 41366.7409217865 | 419.528771543734 |
| United States of America | 2028 | 65 to 69 | 59121.6408692202 | 609.087461163976 | 555039.015265977 | 5718.16511999603 | 1032.36777898596 | 10.6357377813818 | 68740.1650419515 | 708.180151799416 |
| United States of America | 2028 | 70 to 74 | 68481.960849703 | 816.484707740144 | 778658.347658443 | 9283.6511327798 | 1829.38803333988 | 21.811106679947 | 95825.943092056 | 1142.49674175006 |
| United States of America | 2028 | 75 to 79 | 61088.2738970958 | 996.452761145952 | 843412.62286209 | 13757.4821356384 | 2632.58974296737 | 42.9419780752564 | 104542.426837554 | 1705.26327286081 |
| United States of America | 2028 | 80 to 84 | 37342.5540731249 | 923.912660179884 | 750848.1423 | 18577.1466843305 | 4122.88896685475 | 102.00666257412 | 105785.356058229 | 2617.29365196727 |
| United States of America | 2028 | 85 to 89 | 12918.1987146866 | 633.140132403874 | 450190.281525217 | 22064.4952711373 | 4416.10921353825 | 216.440080689486 | 75399.7917442471 | 3695.45593643049 |
| United States of America | 2028 | 90 to 94 | 3754.85860426093 | 449.817802465756 | 198073.086330207 | 23728.4035994215 | 4474.31750789433 | 536.006247119719 | 52256.6815857602 | 6260.15201967535 |
| United States of America | 2028 | 95 plus | 989.319507211831 | 363.731105036851 | 63176.2377667487 | 23227.2411566328 | 2505.55473568662 | 921.186923029582 | 25156.408358823 | 9248.95157167232 |
| United States of America | 2029 | 30 to 34 | 1216.72806088929 | 10.3772567444795 | 2225.57454247815 | 18.9815285548632 | 4.85429623873957 | 0.0414014273216845 | 491.312730965483 | 4.19031870386373 |
| United States of America | 2029 | 35 to 39 | 3610.98756437712 | 29.5369066955034 | 14612.4175568127 | 119.525644 | 8.60572460201302 | 0.0703925117672629 | 1904.0636975764 | 15.5747287341629 |
| United States of America | 2029 | 40 to 44 | 5328.29848390135 | 45.4307208713253 | 35774.4441614408 | 305.02397565296 | 34.3506913050149 | 0.292884618444376 | 4916.42933347302 | 41.9189971071343 |
| United States of America | 2029 | 45 to 49 | 6794.43012580092 | 60.5004849420488 | 64455.7071089785 | 573.940928845181 | 86.12144609 | 0.766861849475258 | 8846.12689270877 | 78.7696623496724 |
| United States of America | 2029 | 50 to 54 | 11617.6748929744 | 114.310245516847 | 100125.96809352 | 985.173375982807 | 161.074418191421 | 1.58486585823461 | 14078.5934853663 | 138.524058614974 |
| United States of America | 2029 | 55 to 59 | 21280.9508950133 | 209.898158521986 | 183101.6 | 1805.96669964294 | 294.484206807154 | 2.9045550185971 | 24665.9442826536 | 243.285006796763 |
| United States of America | 2029 | 60 to 64 | 36963.9546208829 | 378.158318653128 | 316360.259156613 | 3236.5114858069 | 538.461136974063 | 5.50870598956762 | 41072.0867474763 | 420.186406657537 |
| United States of America | 2029 | 65 to 69 | 59159.9159790935 | 609.566874909132 | 554982.644493214 | 5718.38263516272 | 1037.52621541455 | 10.6903737488411 | 68930.7337233673 | 710.242589860932 |
| United States of America | 2029 | 70 to 74 | 69952.7253677165 | 818.386615901568 | 794490.819505199 | 9294.8580590942 | 1874.57725330027 | 21.9309387376989 | 98037.3741003064 | 1146.95280848705 |
| United States of America | 2029 | 75 to 79 | 64139.5002665502 | 999.241609071176 | 885021.248975923 | 13787.9162328005 | 2771.96453542039 | 43.1849685630655 | 109915.569386831 | 1712.39579291438 |
| United States of America | 2029 | 80 to 84 | 39061.5229703094 | 927.047856215627 | 785015.036775056 | 18630.7765698871 | 4303.01545075453 | 102.123546281511 | 110580.995658988 | 2624.42084098399 |
| United States of America | 2029 | 85 to 89 | 13707.6660562311 | 636.080098311344 | 477201.744161569 | 22143.7063826527 | 4673.685656 | 216.874150518835 | 79899.1264400851 | 3707.57822611951 |
| United States of America | 2029 | 90 to 94 | 3944.69066598539 | 453.03935331289 | 207478.471544434 | 23828.4622379526 | 4673.60128922817 | 536.753191821013 | 54676.0273161953 | 6279.42572801441 |
| United States of America | 2029 | 95 plus | 1032.51199248577 | 367.583267763328 | 65596.8214930344 | 23353.0401339427 | 2599.76065529656 | 925.537450442364 | 26118.0586144901 | 9298.2564880773 |
| United States of America | 2030 | 30 to 34 | 1227.67091355884 | 10.4171234308509 | 2255.22355153837 | 19.1361885673699 | 4.94440897858644 | 0.0419546623233361 | 500.350450805985 | 4.24561040516829 |
| United States of America | 2030 | 35 to 39 | 3604.840705 | 29.6288268995691 | 14623.8906760197 | 120.196358427659 | 8.66707070515155 | 0.0712361956249115 | 1914.04111466588 | 15.7318443470663 |
| United States of America | 2030 | 40 to 44 | 5412.0277728949 | 45.560372216691 | 36401.4649168048 | 306.440461955599 | 35.102913487806 | 0.295508794763496 | 5020.53803026486 | 42.2646725008522 |
| United States of America | 2030 | 45 to 49 | 6814.11030743981 | 60.2648009417908 | 64731.6051261404 | 572.494004581964 | 87.5496680517619 | 0.774299663434954 | 9014.59039111122 | 79.7261081756999 |
| United States of America | 2030 | 50 to 54 | 11807.7346075937 | 113.900500810708 | 101885.696315573 | 982.816113459074 | 165.911824763325 | 1.60042891875364 | 14375.3846744148 | 138.668725896787 |
| United States of America | 2030 | 55 to 59 | 20780.3560752664 | 208.935279588081 | 179057.94036719 | 1800.33107698349 | 292.974124942618 | 2.94569691131601 | 24228.622356701 | 243.60573840968 |
| United States of America | 2030 | 60 to 64 | 36879.7381096061 | 376.580701670466 | 315892.839836482 | 3225.59631320466 | 543.027547139689 | 5.54487925376542 | 41121.971275107 | 419.898339593117 |
| United States of America | 2030 | 65 to 69 | 58698.7973847134 | 608.576747088709 | 550238.975811219 | 5704.76161250657 | 1032.15726725206 | 10.7011887836703 | 68443.87598 | 709.611665919149 |
| United States of America | 2030 | 70 to 74 | 71028.4916801965 | 818.343115295467 | 805844.50350049 | 9284.40525539392 | 1908.51198666303 | 21.988607779771 | 99606.2384187591 | 1147.59693641639 |
| United States of America | 2030 | 75 to 79 | 67999.8628925022 | 1000.72365552877 | 936939.115410674 | 13788.5151042715 | 2943.90835680711 | 43.3241863593601 | 116554.891423157 | 1715.28635578472 |
| United States of America | 2030 | 80 to 84 | 40387.3614749069 | 928.977491580965 | 810848.338425386 | 18650.880571909 | 4455.14448501193 | 102.475843857455 | 114335.532379405 | 2629.91025383985 |
| United States of America | 2030 | 85 to 89 | 14258.0894163962 | 637.893319583365 | 495856.91614317 | 22184.1654263439 | 4846.62831153728 | 216.83352742044 | 82945.3391741385 | 3710.89535242461 |
| United States of America | 2030 | 90 to 94 | 4184.97040919627 | 454.910833497792 | 219781.215151092 | 23890.457039275 | 4941.3285881802 | 537.127789887343 | 57853.135403123 | 6288.69871788405 |
| United States of America | 2030 | 95 plus | 1052.18214810391 | 370.037154901234 | 66622.8268904165 | 23430.2790238677 | 2632.70193503442 | 925.882971402494 | 26478.3205006032 | 9312.04012752226 |
| United States of America | 2031 | 30 to 34 | 1242.61595202837 | 10.4569901172224 | 2292.35333540564 | 19.2908485798767 | 5.05126147305227 | 0.0425078973249877 | 511.081057331549 | 4.30090210647285 |
| United States of America | 2031 | 35 to 39 | 3585.53351835031 | 29.7207471036349 | 14581.4955328132 | 120.867072893218 | 8.69577144148445 | 0.07207987948256 | 1916.85620518031 | 15.8889599599697 |
| United States of America | 2031 | 40 to 44 | 5496.77118145018 | 45.6900235620566 | 37036.95182 | 307.856948258237 | 35.8671017418335 | 0.298132971082617 | 5126.26858290675 | 42.6103478945701 |
| United States of America | 2031 | 45 to 49 | 6831.65147187482 | 60.0291169415329 | 64988.3727353356 | 571.047080318747 | 88.9661261095089 | 0.78173747739465 | 9182.12888817562 | 80.6825540017275 |
| United States of America | 2031 | 50 to 54 | 12037.5468540291 | 113.490756104568 | 103993.66223014 | 980.45885093534 | 171.402322390968 | 1.61599197927266 | 14723.4257811672 | 138.8133932 |
| United States of America | 2031 | 55 to 59 | 20280.8529134894 | 207.972400654177 | 175013.388407136 | 1794.69545432405 | 291.267679126626 | 2.98683880403492 | 23786.9873342524 | 243.926470022598 |
| United States of America | 2031 | 60 to 64 | 37044.96847 | 375.003084687805 | 317564.751778887 | 3214.68114060241 | 551.328570397435 | 5.58105251796322 | 41451.5238716571 | 419.610272528696 |
| United States of America | 2031 | 65 to 69 | 57803.1358036585 | 607.586619268286 | 541430.245433991 | 5691.14058985042 | 1019.09323183535 | 10.7120038184994 | 67449.3295458841 | 708.980741977365 |
| United States of America | 2031 | 70 to 74 | 71942.1690027759 | 818.299614689365 | 815334.924551715 | 9273.95245169363 | 1938.23502361186 | 22.0462768218431 | 100949.519252121 | 1148.24106434572 |
| United States of America | 2031 | 75 to 79 | 70133.8539221547 | 1002.20570198636 | 964955.301465476 | 13789.1139757426 | 3041.54729111063 | 43.4634041556546 | 120237.161679022 | 1718.17691865507 |
| United States of America | 2031 | 80 to 84 | 42411.3932870433 | 930.907126946303 | 850635.307110482 | 18670.984573931 | 4684.7688894739 | 102.828141433399 | 120066.72684892 | 2635.39966669571 |
| United States of America | 2031 | 85 to 89 | 15401.1908537098 | 639.706540855386 | 535066.723965882 | 22224.6244700351 | 5219.37588871495 | 216.792904322045 | 89421.1511104094 | 3714.21247872972 |
| United States of America | 2031 | 90 to 94 | 4397.02766191784 | 456.782313682693 | 230568.457138257 | 23952.4518405975 | 5174.04636165697 | 537.502387953672 | 60624.8424762906 | 6297.97170775368 |
| United States of America | 2031 | 95 plus | 1101.3032899825 | 372.491042039139 | 69502.0923887401 | 23507.5179137926 | 2738.47789823441 | 926.228492362624 | 27572.6372911776 | 9325.82376696723 |
| United States of America | 2032 | 30 to 34 | 1257.61109382484 | 10.4968568035938 | 2329.73429936428 | 19.4455085923834 | 5.15908321318632 | 0.0430611323266394 | 521.908392390029 | 4.35619380777741 |
| United States of America | 2032 | 35 to 39 | 3566.64769528363 | 29.8126673077006 | 14540.2108673814 | 121.537787358777 | 8.72423310651049 | 0.0729235633402086 | 1919.67722544409 | 16.046075572873 |
| United States of America | 2032 | 40 to 44 | 5577.633608 | 45.8196749074222 | 37647.8861164388 | 309.273434560875 | 36.6111976289283 | 0.300757147401738 | 5229.04101048563 | 42.9560232882881 |
| United States of America | 2032 | 45 to 49 | 6854.61989528907 | 59.7934329412749 | 65298.0163539469 | 569.60015605553 | 90.4697453698785 | 0.789175291354346 | 9358.95941951412 | 81.638999827755 |
| United States of America | 2032 | 50 to 54 | 12235.2082507042 | 113.081011398429 | 105829.232304928 | 978.101588411607 | 176.531987443955 | 1.63155503979169 | 15035.0689901127 | 138.958060460413 |
| United States of America | 2032 | 55 to 59 | 20058.676149557 | 207.009521720272 | 173355.174570344 | 1789.0598316646 | 293.403335646407 | 3.02798069675384 | 23666.9090258691 | 244.247201635515 |
| United States of America | 2032 | 60 to 64 | 36993.2408537683 | 373.425467705144 | 317379.762075889 | 3203.76596800016 | 556.46816904721 | 5.61722578216101 | 41539.9823622143 | 419.322205464276 |
| United States of America | 2032 | 65 to 69 | 56867.9600599036 | 606.596491447862 | 532263.144509591 | 5677.51956719427 | 1005.25611815723 | 10.7228188533286 | 66407.2570949741 | 708.349818035582 |
| United States of America | 2032 | 70 to 74 | 72469.6709998983 | 818.256114083263 | 820431.109824179 | 9263.49964799335 | 1957.65806938351 | 22.1039458639152 | 101752.165939033 | 1148.88519227506 |
| United States of America | 2032 | 75 to 79 | 71398.6227427564 | 1003.68774844396 | 980949.012116109 | 13789.7128472136 | 3101.72875993422 | 43.6026219519492 | 122430.355475362 | 1721.06748152542 |
| United States of America | 2032 | 80 to 84 | 44449.0094100484 | 932.83676231164 | 890617.099971268 | 18691.0885759529 | 4916.47466068345 | 103.180439009342 | 125836.489609386 | 2640.88907955157 |
| United States of America | 2032 | 85 to 89 | 16904.1227144286 | 641.519762127407 | 586687.622396709 | 22265.0835137263 | 5711.44862051691 | 216.75228122365 | 97957.3511962202 | 3717.52960503482 |
| United States of America | 2032 | 90 to 94 | 4637.43475018144 | 458.653793867595 | 242809.349563053 | 24014.4466419199 | 5438.45806933866 | 537.876986020002 | 63772.3619203284 | 6307.24469762331 |
| United States of America | 2032 | 95 plus | 1158.20863567718 | 374.944929177045 | 72853.5496142515 | 23584.7568037175 | 2862.19639289412 | 926.574013322754 | 28850.1406744813 | 9339.60740641219 |
| United States of America | 2033 | 30 to 34 | 1272.58510746703 | 10.5367234899652 | 2367.23329545288 | 19.6001686048901 | 5.26757624288412 | 0.043614367328291 | 532.802323797421 | 4.41148550908197 |
| United States of America | 2033 | 35 to 39 | 3560.02481443037 | 29.9045875117664 | 14548.4467511151 | 122.208501824336 | 8.78170382431329 | 0.0737672471978571 | 1928.92688025297 | 16.2031911857764 |
| United States of America | 2033 | 40 to 44 | 5637.80220985816 | 45.9493262527879 | 38120.4353854634 | 310.689920863514 | 37.2236991657604 | 0.303381323720859 | 5312.9486856233 | 43.301698682006 |
| United States of America | 2033 | 45 to 49 | 6888.79944208179 | 59.5577489410169 | 65715.9435301018 | 568.153231792313 | 92.1409558456813 | 0.796613105314042 | 9553.47490553694 | 82.5954456537826 |
| United States of America | 2033 | 50 to 54 | 12372.5478468851 | 112.67126669229 | 107147.489442403 | 975.744325887874 | 180.871735126763 | 1.64711810031071 | 15275.0138091835 | 139.102727742227 |
| United States of America | 2033 | 55 to 59 | 20114.7801435465 | 206.046642786368 | 174102.258506637 | 1783.42420900515 | 299.6152972 | 3.06912258947275 | 23875.3232808278 | 244.567933248432 |
| United States of America | 2033 | 60 to 64 | 36586.087721714 | 371.847850722482 | 314144.398188153 | 3192.85079539792 | 556.237592967301 | 5.65339904635881 | 41228.7437351135 | 419.034138399856 |
| United States of America | 2033 | 65 to 69 | 56109.5083189095 | 605.606363627439 | 524760.936458267 | 5663.89854453812 | 994.472575110221 | 10.7336338881577 | 65570.2475412467 | 707.718894093799 |
| United States of America | 2033 | 70 to 74 | 72696.3333648133 | 818.212613477162 | 822112.207699102 | 9253.04684429306 | 1969.00917753113 | 22.1616149059872 | 102133.070668603 | 1149.52932020439 |
| United States of America | 2033 | 75 to 79 | 73530.0803196587 | 1005.16979490155 | 1008787.50381404 | 13790.3117186846 | 3199.79868707955 | 43.7418397482437 | 126110.806467834 | 1723.95804439576 |
| United States of America | 2033 | 80 to 84 | 46006.0238879087 | 934.766397676978 | 920901.280633157 | 18711.1925779748 | 5095.52928342337 | 103.532736585286 | 130245.75170942 | 2646.37849240743 |
| United States of America | 2033 | 85 to 89 | 18000.3989755475 | 643.332983399427 | 624107.073257711 | 22305.5425574175 | 6063.57269029539 | 216.711658125255 | 104108.956666685 | 3720.84673133993 |
| United States of America | 2033 | 90 to 94 | 5011.58340032697 | 460.525274052497 | 262007.540246625 | 24076.4414432423 | 5857.42597853437 | 538.251584086332 | 68738.3667609582 | 6316.51768749294 |
| United States of America | 2033 | 95 plus | 1213.25416671298 | 377.398816314951 | 76068.1105160079 | 23661.9956936425 | 2979.84238042193 | 926.919534282884 | 30069.0944664007 | 9353.39104585716 |
| United States of America | 2034 | 30 to 34 | 1286.40876960616 | 10.5765901763366 | 2402.73891223874 | 19.7548286173969 | 5.3720140444541 | 0.0441676023299427 | 543.284865870488 | 4.46677721038653 |
| United States of America | 2034 | 35 to 39 | 3570.37422623218 | 29.9965077158322 | 14625.8621482624 | 122.879216289895 | 8.88066530141986 | 0.0746109310555056 | 1947.3072759209 | 16.3603067986797 |
| United States of America | 2034 | 40 to 44 | 5662.81190214622 | 46.0789775981535 | 38355.8830807783 | 312.106407166152 | 37.6061205797683 | 0.30600550003998 | 5363.98336718612 | 43.6473740757239 |
| United States of America | 2034 | 45 to 49 | 6940.0225299273 | 59.3220649407589 | 66298.3418738275 | 566.706307529095 | 94.0650245493142 | 0.804050919273738 | 9774.64304162344 | 83.5518914798101 |
| United States of America | 2034 | 50 to 54 | 12450.9366296362 | 112.261521986151 | 107958.456536423 | 973.38706336414 | 184.408133815742 | 1.66268116082974 | 15443.9425068558 | 139.24739502404 |
| United States of America | 2034 | 55 to 59 | 20312.0353422469 | 205.083763852463 | 176076.857175661 | 1777.78858634571 | 308.048774311851 | 3.11026448219166 | 24254.4174250567 | 244.88866486135 |
| United States of America | 2034 | 60 to 64 | 35973.941504905 | 370.270233739821 | 309143.85099413 | 3181.93562279567 | 552.77557534293 | 5.68957231055661 | 40683.6556195192 | 418.746071335436 |
| United States of America | 2034 | 65 to 69 | 55598.664965294 | 604.616235807016 | 519582.287565824 | 5650.27752188197 | 988.02675238866 | 10.7444489229869 | 65021.6531168704 | 707.087970152016 |
| United States of America | 2034 | 70 to 74 | 72793.7332258075 | 818.16911287106 | 822327.4557 | 9242.59404059278 | 1976.88302178476 | 22.2192839480593 | 102332.65694063 | 1150.17344813373 |
| United States of America | 2034 | 75 to 79 | 75213.9452317207 | 1006.65184135914 | 1030414.63911002 | 13790.9105901556 | 3278.65834369182 | 43.8810575445383 | 129024.843778186 | 1726.84860726611 |
| United States of America | 2034 | 80 to 84 | 48428.1122373604 | 936.696033042315 | 968426.577169448 | 18731.2965799968 | 5370.95911231937 | 103.885034161229 | 137104.196051338 | 2651.86790526329 |
| United States of America | 2034 | 85 to 89 | 18881.319782644 | 645.146204671448 | 653994.395439216 | 22346.0016011087 | 6341.25268095164 | 216.671035026859 | 108994.098097456 | 3724.16385764503 |
| United States of America | 2034 | 90 to 94 | 5351.86788892695 | 462.396754237398 | 279382.847397479 | 24138.4362445648 | 6234.16177120951 | 538.626182152662 | 73215.9032000231 | 6325.79067736257 |
| United States of America | 2034 | 95 plus | 1282.61823553298 | 379.852703452856 | 80158.3742795654 | 23739.2345835674 | 3131.02173083466 | 927.265055243014 | 31629.3893858849 | 9367.17468530212 |
| United States of America | 2035 | 30 to 34 | 1294.9261604839 | 10.5967519429283 | 2423.72375012521 | 19.8340261723209 | 5.43242526671493 | 0.0444550930829618 | 549.350962703897 | 4.4954963912434 |
| United States of America | 2035 | 35 to 39 | 3594.19465202775 | 30.0428949185685 | 14741.4982779595 | 123.220172134288 | 8.97836090208878 | 0.0750476752755332 | 1966.95085294419 | 16.4412068644229 |
| United States of America | 2035 | 40 to 44 | 5644.82983004006 | 46.1443559136045 | 38267.7523088852 | 312.824449225661 | 37.5983565853443 | 0.307352391529654 | 5361.03444726778 | 43.8244356425644 |
| United States of America | 2035 | 45 to 49 | 7038.98251710786 | 59.4020748760656 | 67290.1889076043 | 567.862873675164 | 95.6229812233606 | 0.806963716232745 | 9935.90690039644 | 83.8492614840972 |
| United States of America | 2035 | 50 to 54 | 12481.1738124729 | 111.743933246378 | 108279.361630589 | 969.424986768266 | 186.657937707368 | 1.67114827855651 | 15669.8048069366 | 140.2917425 |
| United States of America | 2035 | 55 to 59 | 20639.0879015785 | 204.217171025671 | 179001.774906043 | 1771.16528861192 | 316.140704913846 | 3.12811112154957 | 24702.1859346327 | 244.420226018563 |
| United States of America | 2035 | 60 to 64 | 35140.7325648912 | 368.41343727909 | 302228.637606003 | 3168.54781040756 | 548.012647705796 | 5.74533336323839 | 39886.6655868702 | 418.169528575561 |
| United States of America | 2035 | 65 to 69 | 55512.9714006105 | 601.815555742858 | 518963.197613163 | 5626.07472274888 | 994.617938215025 | 10.7826429055481 | 65033.950149562 | 705.032389078375 |
| United States of America | 2035 | 70 to 74 | 72287.2832293266 | 816.269765890662 | 815793.029018029 | 9211.95478738957 | 1965.77315989332 | 22.1975584824481 | 101563.488560354 | 1146.85739076947 |
| United States of America | 2035 | 75 to 79 | 76490.5186783471 | 1005.83322346713 | 1046547.42393367 | 13761.8647005515 | 3339.22729387815 | 43.9100925307448 | 131136.67028274 | 1724.41790256252 |
| United States of America | 2035 | 80 to 84 | 51413.1337132185 | 937.374641545218 | 1026380.86645419 | 18713.1833307023 | 5705.46902878935 | 104.023264085413 | 145416.461542448 | 2651.26230728239 |
| United States of America | 2035 | 85 to 89 | 19529.667512941 | 646.04815614919 | 675549.846785685 | 22347.4225873821 | 6561.49782146707 | 217.056617391299 | 112611.843561589 | 3725.23873462349 |
| United States of America | 2035 | 90 to 94 | 5588.58604893864 | 463.385447585942 | 291359.644877099 | 24158.4934485345 | 6488.65844334956 | 538.016212092657 | 76239.0094326458 | 6321.46435611648 |
| United States of America | 2035 | 95 plus | 1359.67515088666 | 381.120805166165 | 84829.6422241341 | 23777.9895626811 | 3307.24545352958 | 927.030290514155 | 33417.5193588626 | 9367.02555489104 |
| United States of America | 2036 | 30 to 34 | 1305.22415483458 | 10.61691371 | 2448.09568209255 | 19.913223727245 | 5.50057227271876 | 0.044742583835981 | 556.198873603821 | 4.52421557210026 |
| United States of America | 2036 | 35 to 39 | 3629.2205684181 | 30.0892821213048 | 14903.3328648162 | 123.56112797868 | 9.10455778652223 | 0.0754844194955607 | 1992.81491870839 | 16.5221069301661 |
| United States of America | 2036 | 40 to 44 | 5606.57067813555 | 46.2097342290555 | 38041.7279457902 | 313.542491285169 | 37.4541073956098 | 0.308699283019328 | 5338.6479745905 | 44.0014972094049 |
| United States of America | 2036 | 45 to 49 | 7138.89884651989 | 59.4820848113722 | 68292.364591272 | 569.019439821233 | 97.1994597059378 | 0.809876513191752 | 10099.0792837193 | 84.1466314883843 |
| United States of America | 2036 | 50 to 54 | 12507.4013275042 | 111.226344506604 | 108566.294594256 | 965.462910172392 | 188.872734515901 | 1.67961539628328 | 15893.241899623 | 141.33608993316 |
| United States of America | 2036 | 55 to 59 | 21033.8802158914 | 203.350578198879 | 182518.118221145 | 1764.54199087812 | 325.406985774417 | 3.14595776090748 | 25233.5287922786 | 243.951787175775 |
| United States of America | 2036 | 60 to 64 | 34310.4310283137 | 366.556640818359 | 295329.254583018 | 3155.15999801946 | 542.993981508016 | 5.80109441592017 | 39087.5344823901 | 417.592985815687 |
| United States of America | 2036 | 65 to 69 | 55795.7377774392 | 599.014875678699 | 521791.009878889 | 5601.87192361579 | 1007.91583323757 | 10.8208368881094 | 65479.3582523517 | 702.976808004735 |
| United States of America | 2036 | 70 to 74 | 71242.1096344768 | 814.370418910264 | 803192.59232242 | 9181.31553418637 | 1939.96870507084 | 22.1758330168369 | 100038.379531301 | 1143.54133340521 |
| United States of America | 2036 | 75 to 79 | 77591.4290129112 | 1005.01460557512 | 1060232.38866966 | 13732.8188109475 | 3392.28870377441 | 43.9391275169513 | 132944.781779916 | 1721.98719785893 |
| United States of America | 2036 | 80 to 84 | 53156.3727239693 | 938.053250048121 | 1059387.74083142 | 18695.0700814079 | 5902.48709097864 | 104.161494009596 | 150203.942042408 | 2650.6567093015 |
| United States of America | 2036 | 85 to 89 | 20623.3267324066 | 646.950107626932 | 712431.295206966 | 22348.8435736554 | 6931.57243210752 | 217.442199755739 | 118786.568258516 | 3726.31361160194 |
| United States of America | 2036 | 90 to 94 | 6087.45275106171 | 464.374140934486 | 316955.169790639 | 24178.5506525042 | 7044.82618243152 | 537.406242032653 | 82810.9834708339 | 6317.13803487039 |
| United States of America | 2036 | 95 plus | 1437.66757319476 | 382.388906879474 | 89543.8144537856 | 23816.7445417947 | 3484.47313829498 | 926.795525785296 | 35216.6452931173 | 9366.87642447995 |
| United States of America | 2037 | 30 to 34 | 1320.03968606376 | 10.6370754761117 | 2481.0192963504 | 19.9924212821691 | 5.588141746 | 0.0450300745890002 | 565.010051443127 | 4.55293475295713 |
| United States of America | 2037 | 35 to 39 | 3664.16469201406 | 30.1356693240411 | 15065.1255138802 | 123.902083823073 | 9.23117533816812 | 0.0759211637155883 | 2018.74235350533 | 16.6030069959092 |
| United States of America | 2037 | 40 to 44 | 5569.0196040188 | 46.2751125445065 | 37819.9635772399 | 314.260533344678 | 37.3127828123812 | 0.310046174509003 | 5316.70797484456 | 44.1785587762454 |
| United States of America | 2037 | 45 to 49 | 7233.43163585209 | 59.5620947466789 | 69244.1925877302 | 570.176005967302 | 98.7080110988662 | 0.812789310150759 | 10255.1784730353 | 84.4440014926714 |
| United States of America | 2037 | 50 to 54 | 12543.0783966953 | 110.708755766831 | 108936.102212536 | 961.500833576518 | 191.256338910661 | 1.68808251401005 | 16131.4159475452 | 142.380437387721 |
| United States of America | 2037 | 55 to 59 | 21371.2896768084 | 202.483985372086 | 185540.54806076 | 1757.91869314433 | 333.925570432563 | 3.16380440026539 | 25698.5912201472 | 243.483348332988 |
| United States of America | 2037 | 60 to 64 | 33945.4299971629 | 364.699844357629 | 292428.991797983 | 3141.77218563136 | 545.142753387001 | 5.85685546860195 | 38814.9397221448 | 417.016443055812 |
| United States of America | 2037 | 65 to 69 | 55742.1161248363 | 596.21419561454 | 521475.473629676 | 5577.6691244827 | 1015.24815116519 | 10.8590308706706 | 65531.5366748087 | 700.921226931094 |
| United States of America | 2037 | 70 to 74 | 70139.5226790475 | 812.471071929866 | 789965.438417534 | 9150.67628098316 | 1912.53397531104 | 22.1541075512257 | 98434.0973742399 | 1140.22527604095 |
| United States of America | 2037 | 75 to 79 | 78258.7004533237 | 1004.19598768311 | 1067958.32017427 | 13703.7729213435 | 3426.51364974734 | 43.9681625031577 | 134007.960757634 | 1719.55649315533 |
| United States of America | 2037 | 80 to 84 | 54272.679141623 | 938.731858551024 | 1079806.20478339 | 18676.9568321134 | 6030.07706626183 | 104.299723933779 | 153212.413495378 | 2650.0511113206 |
| United States of America | 2037 | 85 to 89 | 21772.3317577278 | 647.852059104674 | 751124.223552299 | 22350.2645599288 | 7320.52738232376 | 217.827782120179 | 125266.158566297 | 3727.3884885804 |
| United States of America | 2037 | 90 to 94 | 6740.44559334899 | 465.362834283029 | 350499.411803369 | 24198.607856474 | 7775.10750620799 | 536.796271972648 | 91436.5324474142 | 6312.8117136243 |
| United States of America | 2037 | 95 plus | 1527.09301072237 | 383.657008592783 | 94953.4760730429 | 23855.4995209084 | 3688.0453908786 | 926.560761056437 | 37282.94665 | 9366.72729406887 |
| United States of America | 2038 | 30 to 34 | 1332.28392762569 | 10.6572372427034 | 2509.19582337313 | 20.0716188370931 | 5.66524536982002 | 0.0453175653420194 | 572.762317188967 | 4.581653934 |
| United States of America | 2038 | 35 to 39 | 3698.85267859458 | 30.1820565267774 | 15226.1559666426 | 124.243039667465 | 9.35776699142267 | 0.0763579079356158 | 2044.63583419724 | 16.6839070616524 |
| United States of America | 2038 | 40 to 44 | 5550.35583094149 | 46.3404908599575 | 37726.039154387 | 314.978575404187 | 37.2965906814326 | 0.311393065998677 | 5312.62123981955 | 44.3556203430859 |
| United States of America | 2038 | 45 to 49 | 7301.09122675049 | 59.6421046819855 | 69939.7053818869 | 571.332572113371 | 99.8542142269474 | 0.815702107109767 | 10373.6192288687 | 84.7413714969585 |
| United States of America | 2038 | 50 to 54 | 12598.816171294 | 110.191167027057 | 109481.141742751 | 957.538756980644 | 193.976681728765 | 1.69654963173682 | 16398.6147654792 | 143.424784842281 |
| United States of America | 2038 | 55 to 59 | 21603.2955465326 | 201.617392545294 | 187651.231566423 | 1751.29539541054 | 340.913781629596 | 3.18165103962331 | 26039.0378312786 | 243.014909490201 |
| United States of America | 2038 | 60 to 64 | 34048.0414021831 | 362.843047896898 | 293557.672606684 | 3128.38437324325 | 554.821191362893 | 5.91261652128373 | 39077.4001292868 | 416.439900295938 |
| United States of America | 2038 | 65 to 69 | 55150.8255298705 | 593.413515550381 | 516129.55278125 | 5553.46632534961 | 1012.76922566038 | 10.8972248532319 | 64951.3640884244 | 698.865645857454 |
| United States of America | 2038 | 70 to 74 | 69257.8095863334 | 810.571724949468 | 779244.782970528 | 9120.03702777996 | 1891.06066372229 | 22.1323820856145 | 97141.1162768677 | 1136.90921867669 |
| United States of America | 2038 | 75 to 79 | 78602.7770701689 | 1003.3773697911 | 1071253.50115781 | 13674.7270317395 | 3446.66125636131 | 43.9971974893642 | 134516.54344089 | 1717.12578845174 |
| United States of America | 2038 | 80 to 84 | 56007.4923459757 | 939.410467053926 | 1112437.08240426 | 18658.843582819 | 6226.57305455959 | 104.437953857963 | 157959.491099576 | 2649.44551333971 |
| United States of America | 2038 | 85 to 89 | 22638.4852880969 | 648.754010582416 | 779969.443190954 | 22351.6855462021 | 7614.62736410107 | 218.213364484619 | 130105.959534087 | 3728.46336555885 |
| United States of America | 2038 | 90 to 94 | 7222.40009897599 | 466.351527631573 | 375075.192351043 | 24218.6650604437 | 8303.93334331017 | 536.186301912644 | 97699.7025263231 | 6308.48539237821 |
| United States of America | 2038 | 95 plus | 1659.53917581597 | 384.925110306093 | 103016.01625358 | 23894.254500022 | 3993.69705774698 | 926.325996327578 | 40382.4094343227 | 9366.57816365778 |
| United States of America | 2039 | 30 to 34 | 1337.19292717026 | 10.6773990092952 | 2523.60421603188 | 20.1508163920172 | 5.71138705225871 | 0.0456050560950386 | 577.383903625491 | 4.61037311467087 |
| United States of America | 2039 | 35 to 39 | 3730.16547118257 | 30.2284437295137 | 15373.5641331269 | 124.583995511858 | 9.47639787233815 | 0.0767946521556434 | 2068.76362002683 | 16.7648071273955 |
| United States of America | 2039 | 40 to 44 | 5557.77850321036 | 46.4058691754085 | 37809.2664840278 | 315.696617463695 | 37.4551634030111 | 0.312739957488351 | 5333.43705456229 | 44.5326819099264 |
| United States of America | 2039 | 45 to 49 | 7323.59809629899 | 59.7221146172922 | 70203.1465224561 | 572.48913825944 | 100.385034780804 | 0.818614904068774 | 10428.123139328 | 85.0387415012456 |
| United States of America | 2039 | 50 to 54 | 12685.4218509933 | 109.673578287284 | 110295.685130874 | 953.57668038477 | 197.211188580894 | 1.7050167494636 | 16710.0583043972 | 144.469132296841 |
| United States of America | 2039 | 55 to 59 | 21733.1611270452 | 200.750799718502 | 188877.154491226 | 1744.67209767675 | 346.375699027899 | 3.19949767898122 | 26257.9353844066 | 242.546470647413 |
| United States of America | 2039 | 60 to 64 | 34389.1567006282 | 360.986251436167 | 296748.434121757 | 3114.99656085515 | 568.574206976159 | 5.96837757396551 | 39616.9940308264 | 415.863357536063 |
| United States of America | 2039 | 65 to 69 | 54254.9826037458 | 590.612835486223 | 507930.201312737 | 5529.26352621653 | 1004.55141346902 | 10.9354188357931 | 64010.4915969823 | 696.810064783813 |
| United States of America | 2039 | 70 to 74 | 68693.508079132 | 808.67237796907 | 772108.25604113 | 9089.39777457676 | 1878.2125005625 | 22.1106566200033 | 96294.2386886409 | 1133.59316131243 |
| United States of America | 2039 | 75 to 79 | 78821.2893813564 | 1002.55875189909 | 1072825.08887643 | 13645.6811421355 | 3461.34768037726 | 44.0262324755707 | 134809.53324769 | 1714.69508374815 |
| United States of America | 2039 | 80 to 84 | 57433.4635500017 | 940.089075556829 | 1138830.06833343 | 18640.7303335245 | 6388.93972456029 | 104.576183782146 | 161827.272206562 | 2648.83991535881 |
| United States of America | 2039 | 85 to 89 | 23927.7061182244 | 649.655962060158 | 823295.090284892 | 22353.1065324755 | 8051.2943210291 | 218.598946849059 | 137363.928349276 | 3729.53824253731 |
| United States of America | 2039 | 90 to 94 | 7595.58714354563 | 467.340220980116 | 393947.105219058 | 24238.7222644134 | 8704.61500633457 | 535.576331852639 | 102460.236551296 | 6304.15907113212 |
| United States of America | 2039 | 95 plus | 1781.62128307698 | 386.193212019402 | 110409.913300002 | 23933.0094791357 | 4272.32741782205 | 926.091231598719 | 43210.0533947866 | 9366.4290332467 |
| United States of America | 2040 | 30 to 34 | 1334.90121750841 | 10.6977142182134 | 2524.53545599757 | 20.2312789049793 | 5.72757348121706 | 0.0458999838057243 | 578.975050885539 | 4.63982619283964 |
| United States of America | 2040 | 35 to 39 | 3753.22689437672 | 30.2751174259649 | 15487.4990061813 | 124.928725132276 | 9.5756891115963 | 0.0772415631792638 | 2088.5711763321 | 16.8472995093041 |
| United States of America | 2040 | 40 to 44 | 5594.18552734219 | 46.4716180088427 | 38090.3283619379 | 316.421252176159 | 37.8121584295316 | 0.3141104588038 | 5382.43810833353 | 44.7126049903374 |
| United States of America | 2040 | 45 to 49 | 7301.26830071989 | 59.8025556214171 | 70037.3077515859 | 573.655126736755 | 100.305098375585 | 0.821569757151123 | 10419.1641937317 | 85.3403300030597 |
| United States of America | 2040 | 50 to 54 | 12866.6729176122 | 109.804351080173 | 111943.789424833 | 955.329729290301 | 200.412866517471 | 1.71032596359433 | 16982.5896032304 | 144.929636665519 |
| United States of America | 2040 | 55 to 59 | 21805.4386500588 | 199.925960118657 | 189597.297581508 | 1738.34713271331 | 350.518521594093 | 3.21377400811344 | 26631.2394430345 | 244.171933444877 |
| United States of America | 2040 | 60 to 64 | 34980.5148278541 | 359.622741227221 | 301985.948742456 | 3104.61453278523 | 583.464249461791 | 5.99839693132397 | 40381.4130678069 | 415.147533806732 |
| United States of America | 2040 | 65 to 69 | 53079.6226431162 | 587.912710547362 | 497326.529621317 | 5508.41497164826 | 995.773731977243 | 11.0292425738359 | 62833.1544535495 | 695.943345253192 |
| United States of America | 2040 | 70 to 74 | 68722.3407416628 | 805.136256254037 | 772800.6457 | 9053.96719597418 | 1893.05489565805 | 22.1786265590554 | 96501.671640949 | 1130.59295985456 |
| United States of America | 2040 | 75 to 79 | 78451.5121810977 | 1000.3236444422 | 1066894.61213178 | 13603.8156177256 | 3449.86177030553 | 43.988677884597 | 134118.790734942 | 1710.12889116092 |
| United States of America | 2040 | 80 to 84 | 58604.1525471397 | 939.314072783022 | 1160695.28522928 | 18603.7570418204 | 6528.17484076742 | 104.634334445643 | 165048.50720092 | 2645.41638719117 |
| United States of America | 2040 | 85 to 89 | 25480.2484435299 | 650.162409995101 | 875213.677910184 | 22332.2404156249 | 8576.84145364053 | 218.849510906586 | 146131.625176731 | 3728.74266952171 |
| United States of America | 2040 | 90 to 94 | 7850.03289709832 | 468.03723874082 | 406563.101891625 | 24240.23875 | 8996.14598845075 | 536.371169769259 | 105762.335450474 | 6305.79668848491 |
| United States of America | 2040 | 95 plus | 1871.88693879377 | 387.049384610916 | 115843.555687033 | 23952.9300678273 | 4474.3028785389 | 925.149986257862 | 45270.2160326263 | 9360.50617881982 |
| United States of America | 2041 | 30 to 34 | 1324.76590758143 | 10.7180294271316 | 2510.56434739606 | 20.3117414179413 | 5.70976636113405 | 0.0461949115164101 | 577.130528821851 | 4.66927927100841 |
| United States of America | 2041 | 35 to 39 | 3781.33505723448 | 30.3217911224161 | 15622.457930826 | 125.273454752694 | 9.68828489914795 | 0.0776884742028842 | 2111.26101790868 | 16.9297918912127 |
| United States of America | 2041 | 40 to 44 | 5647.75203666896 | 46.5373668422768 | 38488.6694313187 | 317.145886888623 | 38.2866147344016 | 0.315480960119249 | 5448.13520932727 | 44.8925280707484 |
| United States of America | 2041 | 45 to 49 | 7253.01717351629 | 59.882996625542 | 69622.2242587163 | 574.82111521407 | 99.8662641318022 | 0.824524610233472 | 10372.9328973458 | 85.6419185048738 |
| United States of America | 2041 | 50 to 54 | 13049.6465987239 | 109.935123873062 | 113608.750153424 | 957.082778195833 | 203.651317003128 | 1.71563517772507 | 17258.2691736047 | 145.390141034196 |
| United States of America | 2041 | 55 to 59 | 21871.1306642744 | 199.101120518812 | 190261.526633137 | 1732.02216774988 | 354.59926359422 | 3.22805033724566 | 27000.6866668876 | 245.797396242341 |
| United States of America | 2041 | 60 to 64 | 35685.7255169532 | 358.259231018276 | 308212.384465461 | 3094.23250471531 | 600.482528721993 | 6.02841628868243 | 41280.9914466356 | 414.431710077402 |
| United States of America | 2041 | 65 to 69 | 51907.4609682182 | 585.212585608502 | 486738.745218378 | 5487.566417 | 986.598963572187 | 11.1230663118787 | 61652.2332343546 | 695.07662572257 |
| United States of America | 2041 | 70 to 74 | 69194.9652935481 | 801.600134539004 | 778489.550274965 | 9018.5366173716 | 1920.34956864296 | 22.2465964981075 | 97334.9908771979 | 1127.59275839668 |
| United States of America | 2041 | 75 to 79 | 77486.9429189574 | 998.088536985313 | 1052886.6065576 | 13561.9500933158 | 3412.16040028236 | 43.9511232936232 | 132411.939995086 | 1705.56269857369 |
| United States of America | 2041 | 80 to 84 | 59638.9583926377 | 938.539070009215 | 1179816.24733792 | 18566.7837501163 | 6652.62743231833 | 104.69248510914 | 167884.009550506 | 2641.99285902352 |
| United States of America | 2041 | 85 to 89 | 26466.9187862143 | 650.668857930045 | 907548.170436606 | 22311.3742987743 | 8912.2198172762 | 219.100074964112 | 151639.765512089 | 3727.94709650612 |
| United States of America | 2041 | 90 to 94 | 8366.10963031613 | 468.734256501523 | 432674.119799619 | 24241.7552259666 | 9587.50005497986 | 537.166007685878 | 112576.979720883 | 6307.43430583771 |
| United States of America | 2041 | 95 plus | 2054.91417305759 | 387.90555720243 | 126995.217438882 | 23972.8506565189 | 4895.9588366582 | 924.208740917005 | 49555.5309776386 | 9354.58332439294 |
| United States of America | 2042 | 30 to 34 | 1308.47287784188 | 10.7383446360498 | 2484.80065292662 | 20.3922039309034 | 5.66481108453802 | 0.0464898392270958 | 572.54298008806 | 4.69873234917717 |
| United States of America | 2042 | 35 to 39 | 3822.30981808643 | 30.3684648188673 | 15810.8624299385 | 125.618184373112 | 9.83446650571828 | 0.0781353852265046 | 2141.24163315721 | 17.0122842731213 |
| United States of America | 2042 | 40 to 44 | 5701.27138148474 | 46.6031156757109 | 38887.2306399557 | 317.870521601087 | 38.7625621820982 | 0.316851461434698 | 5514.01493474404 | 45.0724511511594 |
| United States of America | 2042 | 45 to 49 | 7205.7625266476 | 59.9634376296669 | 69215.9497800081 | 575.987103691386 | 99.437603046654 | 0.827479463315821 | 10327.7684982336 | 85.943507006688 |
| United States of America | 2042 | 50 to 54 | 13222.669637975 | 110.065896665951 | 115188.898313295 | 958.835827101364 | 206.744140084533 | 1.72094439185581 | 17521.6389369062 | 145.850645402873 |
| United States of America | 2042 | 55 to 59 | 21953.056633496 | 198.276280918968 | 191068.38321483 | 1725.69720278645 | 358.988884607826 | 3.24232666637788 | 27394.5426641435 | 247.422859039805 |
| United States of America | 2042 | 60 to 64 | 36293.4610542856 | 356.89572080933 | 313603.107702052 | 3083.8504766454 | 616.094801223329 | 6.05843564604089 | 42071.6207374612 | 413.715886348071 |
| United States of America | 2042 | 65 to 69 | 51432.582382225 | 582.512460669642 | 482680.518972577 | 5466.71786251173 | 990.388464654891 | 11.2168900499215 | 61294.8401991753 | 694.209906191948 |
| United States of America | 2042 | 70 to 74 | 69231.0635131035 | 798.06401282397 | 779273.309811725 | 8983.10603876903 | 1935.76096836122 | 22.3145664371596 | 97557.0098196707 | 1124.5925569388 |
| United States of America | 2042 | 75 to 79 | 76443.8802110784 | 995.853429528427 | 1037831.16529358 | 13520.084568906 | 3370.90126519515 | 43.9135687026495 | 130572.199971904 | 1700.99650598646 |
| United States of America | 2042 | 80 to 84 | 60319.8918731898 | 937.764067235407 | 1191894.85109749 | 18529.8104584122 | 6737.88561986688 | 104.750635772637 | 169720.958925576 | 2638.56933085588 |
| United States of America | 2042 | 85 to 89 | 27165.4774517063 | 651.175305864988 | 929906.727 | 22290.5081819237 | 9150.78441195549 | 219.350639021638 | 155487.853667883 | 3727.15152349052 |
| United States of America | 2042 | 90 to 94 | 8941.32320125102 | 469.431274262226 | 461764.990256376 | 24243.2717067431 | 10246.6155407092 | 537.960845602498 | 120169.776191079 | 6309.0719231905 |
| United States of America | 2042 | 95 plus | 2290.4324948713 | 388.761729793945 | 141356.050995994 | 23992.7712452104 | 5439.53200961139 | 923.267495576148 | 55078.6615114568 | 9348.66046996606 |
| United States of America | 2043 | 30 to 34 | 1292.36142069111 | 10.758659844968 | 2459.23606396981 | 20.4726664438655 | 5.61992188039044 | 0.0467847669377816 | 567.963345270534 | 4.72818542734594 |
| United States of America | 2043 | 35 to 39 | 3856.07019234938 | 30.4151385153185 | 15969.7394686296 | 125.96291399353 | 9.96276410393005 | 0.078582296250125 | 2167.2976656388 | 17.0947766550298 |
| United States of America | 2043 | 40 to 44 | 5754.47493691548 | 46.668864509145 | 39284.175034294 | 318.595156313551 | 39.2381586370703 | 0.318221962750147 | 5579.81549562802 | 45.2523742315704 |
| United States of America | 2043 | 45 to 49 | 7182.75198376127 | 60.0438786337918 | 69041.9675083355 | 577.153092168701 | 99.3407466208469 | 0.83043431639817 | 10317.0738624593 | 86.2450955085021 |
| United States of America | 2043 | 50 to 54 | 13346.7142581137 | 110.196669458841 | 116343.85422488 | 960.588876006896 | 209.079037772066 | 1.72625360598655 | 17720.7997153338 | 146.31114977155 |
| United States of America | 2043 | 55 to 59 | 22069.8682939992 | 197.451441319123 | 192180.510730146 | 1719.37223782301 | 364.002403409097 | 3.2566029955101 | 27837.0399581262 | 249.048321837269 |
| United States of America | 2043 | 60 to 64 | 36723.1637948169 | 355.532210600384 | 317460.646011906 | 3073.46844857548 | 628.880657450521 | 6.08845500339935 | 42659.0573079322 | 413.00006261874 |
| United States of America | 2043 | 65 to 69 | 51661.0123999382 | 579.812335730782 | 485224.450237882 | 5445.86930794347 | 1007.77939557928 | 11.3107137879643 | 61776.5589936583 | 693.343186661326 |
| United States of America | 2043 | 70 to 74 | 68590.8777643325 | 794.527891108937 | 772444.769568256 | 8947.67546016645 | 1932.26422107562 | 22.3825363762118 | 96826.0586155486 | 1121.59235548093 |
| United States of America | 2043 | 75 to 79 | 75643.8349652601 | 993.618322071541 | 1026092.36804521 | 13478.2190444961 | 3340.26647523722 | 43.8760141116758 | 129148.67993708 | 1696.43031339923 |
| United States of America | 2043 | 80 to 84 | 60750.9977790581 | 936.9890645 | 1199008.98767555 | 18492.8371667081 | 6795.42440088771 | 104.808786436133 | 170853.367321416 | 2635.14580268824 |
| United States of America | 2043 | 85 to 89 | 28143.2675469023 | 651.681753799932 | 961727.854365418 | 22269.642065073 | 9483.60971569557 | 219.601203079165 | 160924.91753483 | 3726.35595047493 |
| United States of America | 2043 | 90 to 94 | 9360.4799557567 | 470.12829202293 | 482725.370311848 | 24244.7881875197 | 10726.8842615937 | 538.755683519118 | 125649.256092796 | 6310.70954054329 |
| United States of America | 2043 | 95 plus | 2475.1412332002 | 389.617902385459 | 152546.387920903 | 24012.691833902 | 5859.29886291974 | 922.326250235291 | 59351.9829597404 | 9342.73761553919 |
| United States of America | 2044 | 30 to 34 | 1282.28250192413 | 10.7789750538862 | 2445.0300227412 | 20.5531289568276 | 5.60066874093891 | 0.0470796946484673 | 565.975575191863 | 4.75763850551471 |
| United States of America | 2044 | 35 to 39 | 3869.06229921397 | 30.4618122117697 | 16042.7796813895 | 126.307643613948 | 10.0377785889401 | 0.0790292072737454 | 2181.74557614125 | 17.1772690369384 |
| United States of America | 2044 | 40 to 44 | 5802.50369680981 | 46.7346133425791 | 39646.2950128847 | 319.319791026015 | 39.68014971 | 0.319592464065596 | 5640.8099747164 | 45.4322973119814 |
| United States of America | 2044 | 45 to 49 | 7193.13110684281 | 60.1243196379167 | 69188.7241922683 | 578.319080646017 | 99.7047051043176 | 0.833389169480519 | 10354.2401593531 | 86.5466840103162 |
| United States of America | 2044 | 50 to 54 | 13388.7128350724 | 110.32744225173 | 116784.359528658 | 962.341924912427 | 210.132645888244 | 1.73156282011729 | 17811.375751179 | 146.771654140228 |
| United States of America | 2044 | 55 to 59 | 22240.8635664593 | 196.626601719278 | 193766.511476202 | 1713.04727285958 | 369.976290927221 | 3.27087932464232 | 28354.2582488851 | 250.673784634733 |
| United States of America | 2044 | 60 to 64 | 36980.9817289955 | 354.168700391438 | 319836.119978571 | 3063.08642050556 | 638.868393210452 | 6.11847436075781 | 43049.1906503058 | 412.284238889409 |
| United States of America | 2044 | 65 to 69 | 52251.2317063551 | 577.112210791922 | 491176.604992328 | 5425.02075337521 | 1032.55679161166 | 11.4045375260071 | 62696.2099547229 | 692.476467130705 |
| United States of America | 2044 | 70 to 74 | 67577.0766817486 | 790.991769393903 | 761402.936252355 | 8912.24488156388 | 1918.02196370915 | 22.4505063152639 | 95565.0750019053 | 1118.59215402305 |
| United States of America | 2044 | 75 to 79 | 75208.8289463685 | 991.383214614655 | 1019315.63764462 | 13436.3535200863 | 3325.69601215845 | 43.8384595207021 | 128349.075702382 | 1691.864121 |
| United States of America | 2044 | 80 to 84 | 61101.1962298201 | 936.214061687793 | 1204505.89920056 | 18455.863875004 | 6844.04941557208 | 104.86693709963 | 171757.064648208 | 2631.72227452059 |
| United States of America | 2044 | 85 to 89 | 28990.5677586033 | 652.188201734875 | 988985.456892903 | 22248.7759482224 | 9772.68146689948 | 219.851767136691 | 165605.741217331 | 3725.56037745933 |
| United States of America | 2044 | 90 to 94 | 9950.39384744872 | 470.825309783633 | 512419.948081494 | 24246.3046682963 | 11402.8283469916 | 539.550521435737 | 133404.766094146 | 6312.34715789609 |
| United States of America | 2044 | 95 plus | 2613.55622130263 | 390.474074976974 | 160857.244402021 | 24032.6124225936 | 6167.09703940986 | 921.385004894433 | 62494.0088724054 | 9336.81476111231 |
| United States of America | 2045 | 30 to 34 | 1278.35507757631 | 10.7992902628044 | 2442.48055030897 | 20.6335914697897 | 5.60792307339616 | 0.0473746223591531 | 566.667131256032 | 4.78709158368348 |
| United States of America | 2045 | 35 to 39 | 3861.63479028234 | 30.5084859082209 | 16031.1203979435 | 126.652373234366 | 10.059750075339 | 0.0794761182973658 | 2184.66741900924 | 17.259761418847 |
| United States of America | 2045 | 40 to 44 | 5837.84114155702 | 46.8003621760133 | 39922.0952324101 | 320.044425738479 | 40.0366731601482 | 0.320962965381045 | 5689.63325033733 | 45.6122203923924 |
| United States of America | 2045 | 45 to 49 | 7240.55865123349 | 60.2047606420416 | 69692.0905549055 | 579.485069123332 | 100.583373862736 | 0.836344022562868 | 10444.8552602212 | 86.8482725121304 |
| United States of America | 2045 | 50 to 54 | 13349.2950293594 | 110.458215044619 | 116514.541146801 | 964.094973817959 | 209.907585452604 | 1.73687203424803 | 17793.5658380035 | 147.232158508905 |
| United States of America | 2045 | 55 to 59 | 22445.0066180061 | 195.801762119433 | 195643.76275869 | 1706.72230789615 | 376.581597591413 | 3.28515565377454 | 28921.3856761894 | 252.299247432197 |
| United States of America | 2045 | 60 to 64 | 37134.9019064194 | 352.805190182493 | 321315.789327689 | 3052.70439243565 | 647.166531128355 | 6.14849371811627 | 43320.0903786149 | 411.568415160079 |
| United States of America | 2045 | 65 to 69 | 53148.8510884397 | 574.412085853061 | 500033.948666167 | 5404.17219880694 | 1063.91335703964 | 11.4983612640499 | 63992.8448439944 | 691.609747600083 |
| United States of America | 2045 | 70 to 74 | 66211.6725965648 | 787.45564767887 | 746389.62087661 | 8876.8143029613 | 1893.42216481547 | 22.518476254316 | 93802.3739271352 | 1115.59195256517 |
| United States of America | 2045 | 75 to 79 | 75578.9373780026 | 989.148107157768 | 1023447.51216733 | 13394.4879956765 | 3346.74436197022 | 43.8009049297283 | 128923.24570257 | 1687.29792822477 |
| United States of America | 2045 | 80 to 84 | 61079.1004673839 | 935.439058913986 | 1202653.72470252 | 18418.8905832999 | 6851.0395369565 | 104.925087763127 | 171613.662757643 | 2628.29874635295 |
| United States of America | 2045 | 85 to 89 | 29765.0951867526 | 652.694649669819 | 1013668.26320398 | 22227.9098313718 | 10037.4146503824 | 220.102331194217 | 169861.939282969 | 3724.76480444373 |
| United States of America | 2045 | 90 to 94 | 10629.8343193449 | 471.522327544337 | 546634.393247297 | 24247.8211490729 | 12181.3566603638 | 540.345359352357 | 142340.262878539 | 6313.98477524888 |
| United States of America | 2045 | 95 plus | 2700.09652887058 | 391.330247568488 | 165957.426745935 | 24052.5330112852 | 6350.86864773017 | 920.443759553576 | 64381.1946688291 | 9330.89190668543 |

AF/AFL, atrial fibrillation and atrial flutter; DALYs, disability-adjusted life years; ASIR, age-standardized incidence rate; ASPR, age-standardized Prevalence rate; ASDAR, age-standardized DALYs rate; ASDR, age-standardized Deaths rate;

**Supplymental Table4** Prediction of future burden of atrial fibrillation/atrial flutter in different age groups in mainland China and the United States in Female.

|  | Location | Age | Incidence Number(N) | Incidence ASR (per 100 000) | Prevalence Number(N) | Prevalence ASR (per 100 000) | Deaths Number(N) | Deaths ASR (per 100 000) | DALYs Number(N) | DALYs ASR (per 100 000) |
| --- | --- | --- | --- | --- | --- | --- | --- | --- | --- | --- |
| China | 2022 | 30 to 34 | 2596.28440042598 | 4.58513514101849 | 4562.69230620271 | 8.05788488633689 | 0.967527273223434 | 0.00170868927133818 | 431.37414900966 | 0.761822845458154 |
| China | 2022 | 35 to 39 | 7374.00702467541 | 13.751507484122 | 28677.0105961136 | 53.4786750968765 | 1.90441077720572 | 0.00355146380630683 | 2492.4609568167 | 4.64809639953596 |
| China | 2022 | 40 to 44 | 11711.5576353946 | 24.6855141996804 | 70537.8738906661 | 148.679086228536 | 16.8267136811506 | 0.0354671933863563 | 6717.31979685592 | 14.1587053055982 |
| China | 2022 | 45 to 49 | 19000.5437594225 | 36.7739403924694 | 155283.546435643 | 300.538129479783 | 64.8874148221741 | 0.125584086176942 | 15680.949799152 | 30.3491479250618 |
| China | 2022 | 50 to 54 | 32411.3975548493 | 52.9697629154551 | 316621.498795908 | 517.452716957773 | 210.039528285494 | 0.34326640797666 | 33944.6276074405 | 55.4755120811048 |
| China | 2022 | 55 to 59 | 42002.7203040797 | 73.2602396471187 | 473180.248540551 | 825.310793048728 | 450.090727779628 | 0.785038548488462 | 53042.0552525905 | 92.5148097801432 |
| China | 2022 | 60 to 64 | 37451.2916703066 | 105.362447598919 | 442857.381100556 | 1245.89928755373 | 539.170159431707 | 1.51685790092695 | 50384.4429699492 | 141.747533808853 |
| China | 2022 | 65 to 69 | 59956.6406634638 | 150.10860992331 | 743136.73126526 | 1860.53155211475 | 1172.50784456908 | 2.93551340977138 | 85471.0620411601 | 213.987010774702 |
| China | 2022 | 70 to 74 | 67248.1258321645 | 227.856093370963 | 806498.171729933 | 2732.64898385186 | 2436.60652958302 | 8.25592740381502 | 109309.010641773 | 370.370531919965 |
| China | 2022 | 75 to 79 | 58011.3043448638 | 333.268804833156 | 700599.721042981 | 4024.87126147697 | 3683.04411913159 | 21.1586987328176 | 109955.686298137 | 631.683782514949 |
| China | 2022 | 80 to 84 | 58487.4822388942 | 511.485725260036 | 663947.745681557 | 5806.36712737248 | 8009.90213678154 | 70.048332512579 | 147042.235094321 | 1285.91625732627 |
| China | 2022 | 85 to 89 | 49650.3898208385 | 743.90597193494 | 549021.361610772 | 8225.92271875154 | 12283.8667194026 | 184.047735455668 | 158800.678540368 | 2379.29195601037 |
| China | 2022 | 90 to 94 | 24631.9886138991 | 990.088204949762 | 268452.423748554 | 10790.504271086 | 13082.7047450767 | 525.862197323025 | 130113.409780636 | 5229.93714997542 |
| China | 2022 | 95 plus | 7079.14603875844 | 1261.31103775988 | 69401.8662529373 | 12365.5225456144 | 4513.00312147673 | 804.09425365686 | 41036.4300315492 | 7311.57428673864 |
| China | 2023 | 30 to 34 | 2501.78012029625 | 4.65918870118627 | 4444.53691782392 | 8.2772806536964 | 0.901737546401907 | 0.00167935037677639 | 408.998220923918 | 0.761697590557378 |
| China | 2023 | 35 to 39 | 7719.91666301867 | 13.9825172146544 | 30014.7706529701 | 54.3635463527081 | 2.02769706287898 | 0.00367261854310267 | 2601.1932860203 | 4.71135006866776 |
| China | 2023 | 40 to 44 | 12340.1668427198 | 25.21693596 | 74165.3033539543 | 151.555625548429 | 18.0931900388883 | 0.0369731479614331 | 7085.38776131517 | 14.4788779369571 |
| China | 2023 | 45 to 49 | 18265.7597793157 | 37.5389717119509 | 148978.274628112 | 306.173480026434 | 62.5556059819279 | 0.128561480702201 | 15074.107037512 | 30.9796298922575 |
| China | 2023 | 50 to 54 | 32857.0542621104 | 53.8387871110029 | 321070.580356538 | 526.098611443554 | 211.690727955341 | 0.346871388556084 | 34386.1953242588 | 56.3444012616493 |
| China | 2023 | 55 to 59 | 42679.5652385548 | 74.1511796699906 | 481793.110036983 | 837.063996936946 | 457.168295650431 | 0.79428101576759 | 53859.2724585254 | 93.5747252026676 |
| China | 2023 | 60 to 64 | 40866.2999374811 | 106.505658387527 | 483917.451946457 | 1261.18456781308 | 583.57730024454 | 1.52091783884634 | 54770.5366598927 | 142.742848658287 |
| China | 2023 | 65 to 69 | 60792.3424165457 | 151.596358407626 | 753435.948405034 | 1878.82456130713 | 1181.42040527861 | 2.94607879988441 | 86204.9846136013 | 214.967234762295 |
| China | 2023 | 70 to 74 | 73493.4007563667 | 230.356759866201 | 880034.125105794 | 2758.36752068513 | 2646.79363864349 | 8.29607557083373 | 118852.593747765 | 372.530024677859 |
| China | 2023 | 75 to 79 | 62553.5334847609 | 336.426978815768 | 754751.059061649 | 4059.22102897626 | 3956.70760381962 | 21.2800638278044 | 118129.542366676 | 635.32726024169 |
| China | 2023 | 80 to 84 | 60477.9374833159 | 516.805488676129 | 685352.605440208 | 5856.58180336751 | 8198.09114810174 | 70.055605040681 | 150912.393785887 | 1289.60008663185 |
| China | 2023 | 85 to 89 | 51200.9560546684 | 749.836248116011 | 566139.725756148 | 8291.09689704147 | 12612.7754650729 | 184.713664779266 | 163005.165282915 | 2387.20506368605 |
| China | 2023 | 90 to 94 | 26203.7646148025 | 995.063168501985 | 285897.521582509 | 10856.6878795747 | 13770.0487559313 | 522.904572946876 | 137060.555909365 | 5204.74493053463 |
| China | 2023 | 95 plus | 7641.60549078573 | 1263.48252590268 | 75127.1517042035 | 12421.714718662 | 4815.55696568814 | 796.216460791187 | 43806.2562138982 | 7243.03803935123 |
| China | 2024 | 30 to 34 | 2391.44883230231 | 4.73324226135405 | 4292.9065920602 | 8.49667642105592 | 0.833660694835991 | 0.00165001148221459 | 384.78091174203 | 0.761572335656601 |
| China | 2024 | 35 to 39 | 8106.41887382766 | 14.2135269451869 | 31509.9001801688 | 55.2484176085396 | 2.16370753281658 | 0.00379377327989851 | 2723.10581352597 | 4.77460373779956 |
| China | 2024 | 40 to 44 | 12823.1636402137 | 25.7483577227396 | 76910.1059859072 | 154.432164868322 | 19.1633126223993 | 0.0384791025365099 | 7370.20392526704 | 14.7990505683161 |
| China | 2024 | 45 to 49 | 17785.7406280684 | 38.3040030314323 | 144782.543525891 | 311.808830573085 | 61.0775932579069 | 0.13153887522746 | 14677.5586430252 | 31.6101118594533 |
| China | 2024 | 50 to 54 | 32602.2749124543 | 54.7078113065508 | 318672.726505971 | 534.744505929335 | 208.860977326402 | 0.350476369135507 | 34095.3764936881 | 57.2132904421938 |
| China | 2024 | 55 to 59 | 43407.5348111722 | 75.0421196928624 | 490991.756948526 | 848.817200825164 | 464.791955567085 | 0.803523483046718 | 54740.6772902166 | 94.6346406251919 |
| China | 2024 | 60 to 64 | 46440.3411435818 | 107.648869176134 | 550676.432160069 | 1276.46984807242 | 657.884181518949 | 1.52497777676572 | 62009.4702316708 | 143.73816350772 |
| China | 2024 | 65 to 69 | 59667.5326404704 | 153.084106891942 | 739438.775577738 | 1897.1175704995 | 1152.41005284407 | 2.95664418999744 | 84169.7567774346 | 215.947458749887 |
| China | 2024 | 70 to 74 | 78927.8606233707 | 232.85742636144 | 943675.964064809 | 2784.0860575184 | 2825.59296298831 | 8.33622373785245 | 127002.357070216 | 374.689517435754 |
| China | 2024 | 75 to 79 | 68109.9488849809 | 339.585152798381 | 821039.716864588 | 4093.57079647555 | 4292.44393632928 | 21.4014289227912 | 128157.146869926 | 638.970737968431 |
| China | 2024 | 80 to 84 | 62942.3035647678 | 522.125252092222 | 712065.496946448 | 5906.79647936253 | 8446.0939034923 | 70.0628775687829 | 155905.63472304 | 1293.28391593743 |
| China | 2024 | 85 to 89 | 52381.5429551504 | 755.766524297083 | 579166.128963516 | 8356.27107533141 | 12848.5039484105 | 185.379594102864 | 166003.628557818 | 2395.11817136173 |
| China | 2024 | 90 to 94 | 27934.8651731758 | 1000.03813205421 | 305117.307573268 | 10922.8714880635 | 14524.094071985 | 519.946948570727 | 144684.589521153 | 5179.55271109384 |
| China | 2024 | 95 plus | 8218.61293598539 | 1265.65401404549 | 81026.1618547981 | 12477.9068917095 | 5119.13232388108 | 788.338667925514 | 46588.1296012425 | 7174.50179196382 |
| China | 2025 | 30 to 34 | 2237.70581490194 | 4.7920140564137 | 4052.18738878375 | 8.67769963189956 | 0.760823074331994 | 0.00162929141192877 | 355.584255831679 | 0.761478448524067 |
| China | 2025 | 35 to 39 | 8427.32567386556 | 14.3544965325795 | 32904.7656150582 | 56.0475959047681 | 2.20349900691729 | 0.00375328070593339 | 2802.8641842889 | 4.7741959634285 |
| China | 2025 | 40 to 44 | 13176.6473783966 | 25.9590019191039 | 78948.4300221878 | 155.53443813153 | 19.7614707873229 | 0.0389316070591283 | 7575.55877111469 | 14.9244294872813 |
| China | 2025 | 45 to 49 | 17638.3382540342 | 38.7333575345217 | 143286.175158162 | 314.652920939749 | 61.764294773845 | 0.135632874134034 | 14603.8267575253 | 32.0696448284786 |
| China | 2025 | 50 to 54 | 31698.8813739363 | 55.4371062703982 | 308822.119340098 | 540.088605857975 | 203.648111984479 | 0.356153325812064 | 33178.7571040676 | 58.025211104461 |
| China | 2025 | 55 to 59 | 44627.2070182176 | 75.9766677585501 | 503646.757135049 | 857.445600817013 | 474.047122390604 | 0.807052986871105 | 56212.2742656418 | 95.6999456427944 |
| China | 2025 | 60 to 64 | 52289.9793337053 | 108.699357795678 | 619976.777449935 | 1288.79526088477 | 740.462748887893 | 1.53925907604739 | 69820.7863607293 | 145.142047002272 |
| China | 2025 | 65 to 69 | 56889.5926821986 | 154.387425935294 | 705104.872778811 | 1913.51917266964 | 1092.57742637062 | 2.96504524886764 | 80124.1676967019 | 217.441599116465 |
| China | 2025 | 70 to 74 | 83457.6373925559 | 234.872122474887 | 996574.028333264 | 2804.6259701435 | 2967.30026060147 | 8.3507768971413 | 133707.75666322 | 376.289403582463 |
| China | 2025 | 75 to 79 | 75147.1302399341 | 342.9817357 | 903959.095549148 | 4125.79241071396 | 4703.00386454606 | 21.4651500797335 | 140733.927047593 | 642.328807798763 |
| China | 2025 | 80 to 84 | 65947.1202044579 | 526.801215238152 | 745223.271611719 | 5953.01999377172 | 8814.20298262533 | 70.4099409982862 | 162889.837289081 | 1301.20259941286 |
| China | 2025 | 85 to 89 | 53810.9750093668 | 762.574132233241 | 594613.86343812 | 8426.48085908592 | 13084.5093881071 | 185.425155195509 | 169536.488402368 | 2402.56082523691 |
| China | 2025 | 90 to 94 | 29689.9848350986 | 1006.82651224465 | 324641.657330096 | 11009.0264240412 | 15360.2619677891 | 520.886725610948 | 153088.334006702 | 5191.42715125609 |
| China | 2025 | 95 plus | 8826.86627437975 | 1270.87008109961 | 87223.3042974431 | 12558.1927221455 | 5465.97365438718 | 786.977185958045 | 49771.8775790012 | 7166.03017753883 |
| China | 2026 | 30 to 34 | 2114.23194260728 | 4.85078585147334 | 3861.10526795235 | 8.8587228427432 | 0.70110143316906 | 0.00160857134164295 | 331.852118314619 | 0.761384561391534 |
| China | 2026 | 35 to 39 | 8509.13997316429 | 14.4954661199721 | 33370.2383004206 | 56.8467742009966 | 2.17948382233064 | 0.00371278813196827 | 2802.31291403316 | 4.77378818905743 |
| China | 2026 | 40 to 44 | 13610.7180599086 | 26.1696461154682 | 81466.0659612399 | 156.636711394737 | 20.4835035374166 | 0.0393841115817468 | 7827.33928342989 | 15.0498084062465 |
| China | 2026 | 45 to 49 | 17920.824914987 | 39.1627120376111 | 145286.372026266 | 317.497011306414 | 63.9389025273457 | 0.139726873040607 | 14885.325086212 | 32.5291777975038 |
| China | 2026 | 50 to 54 | 30428.6494346155 | 56.1664012342455 | 295493.039073961 | 545.432705786615 | 196.024786682633 | 0.361830282488621 | 31875.5415502117 | 58.8371317667281 |
| China | 2026 | 55 to 59 | 45813.6262496958 | 76.9112158242378 | 515893.425327074 | 866.074000808862 | 482.838853544268 | 0.810582490695492 | 57640.0591279755 | 96.765250660397 |
| China | 2026 | 60 to 64 | 58353.7924967545 | 109.749846415222 | 691803.481153922 | 1301.12067369711 | 826.014574583571 | 1.55354037532905 | 77918.2030661085 | 146.545930496824 |
| China | 2026 | 65 to 69 | 54004.3132597948 | 155.690744978646 | 669429.940137601 | 1929.92077483978 | 1031.39673386674 | 2.97344630773785 | 75942.04948 | 218.935739483044 |
| China | 2026 | 70 to 74 | 86637.3599869218 | 236.886818588334 | 1033256.78932583 | 2825.16588276861 | 3059.47842867458 | 8.36533005643015 | 138206.63650502 | 377.889289729172 |
| China | 2026 | 75 to 79 | 82843.6142203352 | 346.37831868482 | 994475.956560483 | 4158.01402495238 | 5149.07950965983 | 21.5288712366757 | 154429.511640741 | 645.686877629096 |
| China | 2026 | 80 to 84 | 69630.4939784386 | 531.477178384081 | 785979.729631398 | 5999.24350818092 | 9270.0973270786 | 70.7570044277894 | 171512.089912023 | 1309.12128288829 |
| China | 2026 | 85 to 89 | 55832.8788114831 | 769.381740169399 | 616592.094914966 | 8496.69064284043 | 13459.3316749569 | 185.470716288155 | 174890.337473946 | 2410.00347911209 |
| China | 2026 | 90 to 94 | 31369.0800150602 | 1013.61489243509 | 343370.677030899 | 11095.1813600189 | 16149.3457108927 | 521.826502651169 | 161030.372759785 | 5203.30159141833 |
| China | 2026 | 95 plus | 9423.11779156485 | 1276.08614815372 | 93327.454638897 | 12638.4785525815 | 5801.29274839097 | 785.615703990575 | 52854.2038778722 | 7157.55856311383 |
| China | 2027 | 30 to 34 | 2050.14417763952 | 4.90955764653298 | 3774.83758688287 | 9.03974605358684 | 0.66305852243718 | 0.00158785127135712 | 317.901480273997 | 0.761290674 |
| China | 2027 | 35 to 39 | 8252.863461 | 14.6364357073648 | 32504.100354366 | 57.645952497225 | 2.07065124570493 | 0.00367229555800314 | 2691.5061562388 | 4.77338041468637 |
| China | 2027 | 40 to 44 | 14078.3027885812 | 26.3802903118325 | 84180.1648627745 | 157.738984657945 | 21.2595061297778 | 0.0398366161043652 | 8098.50382662178 | 15.1751873252117 |
| China | 2027 | 45 to 49 | 18661.3836354729 | 39.5920665407005 | 150990.05217083 | 320.341101673078 | 67.788744076683 | 0.14382087194718 | 15548.9480858749 | 32.9887107665291 |
| China | 2027 | 50 to 54 | 29111.6422211921 | 56.8956961980929 | 281814.238741155 | 550.776805715255 | 188.041275090916 | 0.367507239165178 | 30520.4433583194 | 59.6490524289953 |
| China | 2027 | 55 to 59 | 46949.9603437947 | 77.8457638899255 | 527546.278411305 | 874.702400800711 | 491.003285832776 | 0.814111994519879 | 59003.0912406256 | 97.8305556779995 |
| China | 2027 | 60 to 64 | 62077.8012250137 | 110.800335034766 | 735880.853180839 | 1313.44608650946 | 878.399169480977 | 1.56782167461072 | 82891.4383819558 | 147.949813991376 |
| China | 2027 | 65 to 69 | 53605.9716871334 | 156.994064021998 | 664576.096465714 | 1946.32237700992 | 1018.15840302948 | 2.98184736660806 | 75266.2718289866 | 220.429879849622 |
| China | 2027 | 70 to 74 | 89029.2510911139 | 238.901514701781 | 1060483.25438958 | 2845.70579539371 | 3122.8547372659 | 8.379883216 | 141420.774027272 | 379.489175875881 |
| China | 2027 | 75 to 79 | 91344.2262315189 | 349.774901628039 | 1094286.15491871 | 4190.23563919079 | 5638.93702877821 | 21.5925923936179 | 169499.035634653 | 649.044947459428 |
| China | 2027 | 80 to 84 | 74337.2524044056 | 536.153141530011 | 838199.710400533 | 6045.46702259012 | 9858.52852452545 | 71.1040678572927 | 182606.656237953 | 1317.03996636371 |
| China | 2027 | 85 to 89 | 58071.5763152024 | 776.189348105558 | 640943.364041234 | 8566.90042659494 | 13879.6321875913 | 185.5162774 | 180864.254247109 | 2417.44613298727 |
| China | 2027 | 90 to 94 | 32825.2202246653 | 1020.40327262553 | 359690.953732198 | 11181.3362959967 | 16816.800491776 | 522.76627969139 | 167766.319787032 | 5215.17603158058 |
| China | 2027 | 95 plus | 10038.464709223 | 1281.30221520783 | 99646.1770599007 | 12718.7643830174 | 6144.30244277779 | 784.254222023106 | 56010.0936264586 | 7149.08694868884 |
| China | 2028 | 30 to 34 | 2000.46550583261 | 4.96832944159263 | 3712.68271711502 | 9.22076926443048 | 0.630995176087239 | 0.0015671312010713 | 306.490930945387 | 0.761196787126466 |
| China | 2028 | 35 to 39 | 7901.26714643701 | 14.7774052947574 | 31249.7750854379 | 58.4451307934535 | 1.94187308446443 | 0.00363180298403802 | 2552.04016953811 | 4.7729726403153 |
| China | 2028 | 40 to 44 | 14612.4723652475 | 26.5909345081968 | 87287.7743773357 | 158.841257921153 | 22.1400139810973 | 0.0402891206269837 | 8408.0949222282 | 15.3005662441769 |
| China | 2028 | 45 to 49 | 19459.6991988919 | 40.0214210437899 | 157143.011382535 | 323.185192039742 | 71.9209567973004 | 0.147914870853753 | 16263.609458368 | 33.4482437355544 |
| China | 2028 | 50 to 54 | 27771.2920894116 | 57.6249911619403 | 268012.121065021 | 556.120905643895 | 179.84917822804 | 0.373184195841735 | 29138.0408026247 | 60.4609730912625 |
| China | 2028 | 55 to 59 | 47399.7873210804 | 78.7803119556132 | 531474.058078336 | 883.330800792561 | 491.950744600298 | 0.817641498344265 | 59502.7189857774 | 98.8958606956021 |
| China | 2028 | 60 to 64 | 62937.5055012627 | 111.85082365431 | 746000.327095206 | 1325.77149932181 | 890.235863891908 | 1.58210297389239 | 84040.0530818288 | 149.353697485927 |
| China | 2028 | 65 to 69 | 58462.4037604597 | 158.29738306535 | 724873.396635938 | 1962.72397918006 | 1104.35881761999 | 2.99024842547827 | 81960.9991194087 | 221.9240202 |
| China | 2028 | 70 to 74 | 90246.8849253379 | 240.916210815228 | 1073691.74412968 | 2866.24570801881 | 3144.54444964447 | 8.39443637500785 | 142755.444352538 | 381.08906202259 |
| China | 2028 | 75 to 79 | 99885.7215921515 | 353.171484571259 | 1194216.43047654 | 4222.45725342921 | 6124.94666337403 | 21.6563135505601 | 184515.876840946 | 652.403017289761 |
| China | 2028 | 80 to 84 | 80437.0368088406 | 540.829104675941 | 906011.772880235 | 6091.69053699931 | 10626.8638792896 | 71.451131286796 | 197059.934026964 | 1324.95864983914 |
| China | 2028 | 85 to 89 | 60283.2253545254 | 782.996956041716 | 664974.310825582 | 8637.11021034945 | 14286.4746019508 | 185.561838473445 | 186693.084909391 | 2424.88878686245 |
| China | 2028 | 90 to 94 | 34017.8860272182 | 1027.19165281597 | 373149.676101051 | 11267.4912319744 | 17343.7672520217 | 523.70605673161 | 173106.164481382 | 5227.05047174283 |
| China | 2028 | 95 plus | 10822.8350495286 | 1286.51828226195 | 107672.009920677 | 12799.0502134534 | 6586.08517571804 | 782.892740055637 | 60070.4264994946 | 7140.61533426385 |
| China | 2029 | 30 to 34 | 1959.07593051698 | 5.02710123665227 | 3663.9057928125 | 9.40179247527412 | 0.602640902259378 | 0.00154641113078547 | 296.604007326045 | 0.761102899993932 |
| China | 2029 | 35 to 39 | 7505.28969497332 | 14.91837488 | 29805.2372332208 | 59.244309089682 | 1.8067534315967 | 0.0035913104100729 | 2401.03109017734 | 4.77256486594423 |
| China | 2029 | 40 to 44 | 15215.5620365924 | 26.8015787045612 | 90801.7676090525 | 159.943531184361 | 23.1294854593721 | 0.0407416251496021 | 8757.48507915005 | 15.425945163142 |
| China | 2029 | 45 to 49 | 20018.404795536 | 40.4507755468793 | 161346.378707765 | 326.029282406407 | 75.2266191743978 | 0.152008869760326 | 16780.3853105924 | 33.9077767045797 |
| China | 2029 | 50 to 54 | 26842.1383186564 | 58.3542861257877 | 258265.884843087 | 561.465005572535 | 174.270720021157 | 0.378861152518292 | 28184.6561497009 | 61.2728937535297 |
| China | 2029 | 55 to 59 | 46845.2027541737 | 79.714860021301 | 524168.387149286 | 891.95920078441 | 482.569022666029 | 0.821171002168652 | 58743.1386585556 | 99.9611657132046 |
| China | 2029 | 60 to 64 | 63882.0226041411 | 112.901312273854 | 757124.390017219 | 1338.09691213415 | 903.269006975191 | 1.59638427317405 | 85301.9243264447 | 150.757580980479 |
| China | 2029 | 65 to 69 | 66352.5139991916 | 159.600702108703 | 822803.133743462 | 1979.1255813502 | 1246.66075562365 | 2.99864948434847 | 92884.0314101229 | 223.418160582778 |
| China | 2029 | 70 to 74 | 88507.214095359 | 242.930906928676 | 1051744.94346555 | 2886.78562064391 | 3063.65396831184 | 8.4089895342967 | 139425.374464569 | 382.688948169299 |
| China | 2029 | 75 to 79 | 107296.138052657 | 356.568067514479 | 1280290.22435232 | 4254.67886766762 | 6535.85122955531 | 21.7200347075023 | 197327.350773891 | 655.761087120093 |
| China | 2029 | 80 to 84 | 87853.934840905 | 545.505067821871 | 988514.924865193 | 6137.91405140851 | 11563.1444919227 | 71.7981947162992 | 214660.734243032 | 1332.87733331457 |
| China | 2029 | 85 to 89 | 62968.0850364302 | 789.804563977874 | 694201.187021132 | 8707.31999410397 | 14797.76523 | 185.60739956609 | 193920.445617278 | 2432.33144073762 |
| China | 2029 | 90 to 94 | 34992.6036844239 | 1033.98003300642 | 384237.246412921 | 11353.6461679521 | 17755.3948333816 | 524.645833771831 | 177298.997391386 | 5238.92491190508 |
| China | 2029 | 95 plus | 11674.4367022487 | 1291.73434931606 | 116400.863297463 | 12879.3360438894 | 7063.32320434924 | 781.531258088167 | 64458.8885111596 | 7132.14371983886 |
| China | 2030 | 30 to 34 | 1928.9803461228 | 5.06787793105518 | 3627.70152697406 | 9.53081172960622 | 0.583574722518083 | 0.0015331858944626 | 289.673876805663 | 0.761040334297509 |
| China | 2030 | 35 to 39 | 6981.32418419831 | 15.0154212985061 | 27804.6985216168 | 59.8023027386414 | 1.65765869518983 | 0.00356528976748347 | 2218.84717899236 | 4.77229309376326 |
| China | 2030 | 40 to 44 | 15754.3720677892 | 26.9564296372686 | 94196.5496628375 | 161.174476020616 | 23.7042256829154 | 0.0405589819116866 | 9015.10837494404 | 15.425250434347 |
| China | 2030 | 45 to 49 | 20502.8484257736 | 40.6436996760127 | 164925.293701788 | 326.938675397283 | 77.5011914702058 | 0.153634026123341 | 17227.6877064182 | 34.1512042966478 |
| China | 2030 | 50 to 54 | 26534.5069974259 | 58.8067393297271 | 254625.394051046 | 564.310057697377 | 175.478738430508 | 0.388902361358863 | 27998.629293595 | 62.051580405011 |
| China | 2030 | 55 to 59 | 45404.9195035329 | 80.5074060801701 | 506187.695840581 | 897.520771480552 | 470.030677562835 | 0.833410807517108 | 57090.9342444597 | 101.227864226417 |
| China | 2030 | 60 to 64 | 65500.0082960111 | 113.942776163743 | 774364.496232404 | 1347.07220287073 | 922.205488730059 | 1.60425404993031 | 87551.3643346747 | 152.302965583345 |
| China | 2030 | 65 to 69 | 74536.91112 | 160.708957953267 | 923858.030214122 | 1991.92935544741 | 1403.50344141596 | 3.02609233669804 | 104548.010188948 | 225.415857997916 |
| China | 2030 | 70 to 74 | 84223.1230297428 | 244.355339922877 | 1000728.80852718 | 2903.40015166522 | 2907.32346188977 | 8.43497589782976 | 132717.582449363 | 385.051620107983 |
| China | 2030 | 75 to 79 | 113366.430372544 | 358.773203138405 | 1350805.25083305 | 4274.92270035274 | 6877.51408131681 | 21.7654181090022 | 208002.311378982 | 658.26943009844 |
| China | 2030 | 80 to 84 | 97079.0768988334 | 549.578142675609 | 1090048.28316204 | 6170.91478435895 | 12718.7464894571 | 72.0025910436087 | 236512.164540024 | 1338.92822491039 |
| China | 2030 | 85 to 89 | 66218.9410110984 | 795.125963332345 | 729132.800089647 | 8755.08443983305 | 15527.0836866786 | 186.441933162946 | 203660.396540192 | 2445.45845220519 |
| China | 2030 | 90 to 94 | 36170.0685602833 | 1041.25211870895 | 396850.970850349 | 11424.4161169041 | 18234.5270977262 | 524.929609755053 | 182469.428211372 | 5252.87029544996 |
| China | 2030 | 95 plus | 12521.5920221825 | 1298.32100557134 | 124960.580209233 | 12956.7347240364 | 7552.38202932653 | 783.080634906763 | 68933.9416249587 | 7147.52439226277 |
| China | 2031 | 30 to 34 | 1911.03586693412 | 5.10865462545809 | 3613.53131739109 | 9.65983098393832 | 0.568584010271262 | 0.00151996065813972 | 284.665125430312 | 0.760977768601086 |
| China | 2031 | 35 to 39 | 6557.93824323964 | 15.1124677148621 | 26192.8828251024 | 60.3602963876009 | 1.53583840741205 | 0.00353926912489404 | 2070.78166933539 | 4.77202132158228 |
| China | 2031 | 40 to 44 | 15843.9226456583 | 27.111280569976 | 94910.2687588066 | 162.405420856871 | 23.5960667741596 | 0.0403763386737711 | 9014.16171809504 | 15.4245557055519 |
| China | 2031 | 45 to 49 | 21110.0945189882 | 40.8366238051462 | 169477.862434592 | 327.848068388159 | 80.2597206092929 | 0.155259182486357 | 17779.9696072108 | 34.3946318887159 |
| China | 2031 | 50 to 54 | 26874.8230723996 | 59.2591925336665 | 257212.300394071 | 567.155109822219 | 180.926155193391 | 0.398943570199433 | 28494.3523281311 | 62.8302670564923 |
| China | 2031 | 55 to 59 | 43452.7707815254 | 81.2999521390392 | 482674.700033479 | 903.082342176693 | 451.978891442163 | 0.845650612865564 | 54780.7547716766 | 102.494562739628 |
| China | 2031 | 60 to 64 | 67060.5462111456 | 114.984240053632 | 790867.388149407 | 1356.0474936073 | 940.214974914625 | 1.61212382668656 | 89726.6824771692 | 153.84835018621 |
| China | 2031 | 65 to 69 | 82961.656326287 | 161.817213797831 | 1027801.53616408 | 2004.73312954462 | 1565.50920009344 | 3.05353518904762 | 116592.078094288 | 227.413555413054 |
| China | 2031 | 70 to 74 | 79814.8282240103 | 245.779772917078 | 948249.188873832 | 2920.01468268653 | 2747.62337634817 | 8.46096226136282 | 125809.397592957 | 387.414292046668 |
| China | 2031 | 75 to 79 | 117582.546390721 | 360.978338762332 | 1399077.35145102 | 4295.16653303786 | 7104.49715409634 | 21.8108015105021 | 215237.106534288 | 660.777773076786 |
| China | 2031 | 80 to 84 | 107140.767640286 | 553.651217529348 | 1200561.38206682 | 6203.91551730938 | 13973.2593571015 | 72.2069873709182 | 260275.947094781 | 1344.97911650622 |
| China | 2031 | 85 to 89 | 70261.0362485067 | 800.447362686815 | 772689.515226254 | 8802.84888556214 | 16438.6057508332 | 187.276466759802 | 215807.77254775 | 2458.58546367275 |
| China | 2031 | 90 to 94 | 37814.7003895965 | 1048.52420441148 | 414570.321957421 | 11495.1860658561 | 18941.6579405013 | 525.213385738276 | 189946.074750088 | 5266.81567899484 |
| China | 2031 | 95 plus | 13326.2259565615 | 1304.90766182662 | 133109.653635548 | 13034.1334041834 | 8012.94768544394 | 784.630011725359 | 73150.3800535557 | 7162.90506468669 |
| China | 2032 | 30 to 34 | 1896.28369641895 | 5.14943132 | 3604.75477008984 | 9.78885023827042 | 0.554856961425661 | 0.00150673542181684 | 280.207852867213 | 0.760915202904663 |
| China | 2032 | 35 to 39 | 6323.25526935585 | 15.2095141312182 | 25326.3776311686 | 60.9182900365603 | 1.46060990421064 | 0.00351324848230461 | 1983.82342933406 | 4.77174954940131 |
| China | 2032 | 40 to 44 | 15305.8712758081 | 27.2661315026834 | 91857.4440636562 | 163.636365693125 | 22.5627727343608 | 0.0401936954358555 | 8658.20040011787 | 15.4238609767568 |
| China | 2032 | 45 to 49 | 21766.3011398475 | 41.0295479342797 | 174406.842546984 | 328.757461379035 | 83.2276233945121 | 0.156884338849372 | 18375.5968933985 | 34.638059480784 |
| China | 2032 | 50 to 54 | 27899.0602956525 | 59.711645737606 | 266321.061666493 | 570.000161947061 | 191.089876513905 | 0.408984779040004 | 29719.9987191858 | 63.6089537079737 |
| China | 2032 | 55 to 59 | 41448.4662194247 | 82.0924981979083 | 458773.911806173 | 908.643912872835 | 433.148494684475 | 0.85789041821402 | 52389.0151516042 | 103.76126125284 |
| China | 2032 | 60 to 64 | 68533.9688245391 | 116.025703943522 | 806290.552587786 | 1365.02278434388 | 956.896509486785 | 1.61999360344282 | 91787.8453962863 | 155.393734789076 |
| China | 2032 | 65 to 69 | 88031.7561582941 | 162.925469642396 | 1090113.88539549 | 2017.53690364183 | 1664.71152892574 | 3.08097804139719 | 123955.300010901 | 229.411252828191 |
| China | 2032 | 70 to 74 | 79205.2683036807 | 247.204205911279 | 940908.363280984 | 2936.62921370785 | 2719.25406947035 | 8.48694862489587 | 124886.180221926 | 389.776963985353 |
| China | 2032 | 75 to 79 | 120761.951844603 | 363.183474386259 | 1434914.89984726 | 4315.41036572298 | 7267.38889843548 | 21.856184912002 | 220548.928173573 | 663.286116055133 |
| China | 2032 | 80 to 84 | 118224.465773315 | 557.724292383087 | 1322079.92699985 | 6236.91625025982 | 15349.5145729627 | 72.4113836982277 | 286386.666553642 | 1351.03000810204 |
| China | 2032 | 85 to 89 | 75372.2385009762 | 805.768762041285 | 827893.274487379 | 8850.61333129122 | 17596.0496999421 | 188.111000356658 | 231205.912860355 | 2471.71247514031 |
| China | 2032 | 90 to 94 | 39586.2689346813 | 1055.79629011401 | 433656.614989101 | 11565.9560148082 | 19703.111445933 | 525.497161721498 | 197998.069853908 | 5280.76106253972 |
| China | 2032 | 95 plus | 14040.661445237 | 1311.49431808189 | 140370.095765028 | 13111.5320843304 | 8416.7186068433 | 786.179388543955 | 76849.6503586456 | 7178.2857371106 |
| China | 2033 | 30 to 34 | 1891.56170891366 | 5.19020801426391 | 3614.54918852043 | 9.91786949260252 | 0.544307024109431 | 0.00149351018549396 | 277.291336053164 | 0.76085263720824 |
| China | 2033 | 35 to 39 | 6135.87694528301 | 15.3065605475743 | 24643.7408701485 | 61.4762836855197 | 1.39791044749426 | 0.00348722783971518 | 1912.7223517772 | 4.77147777722033 |
| China | 2033 | 40 to 44 | 14596.4935974734 | 27.4209824353908 | 87760.701070609 | 164.86731052938 | 21.2983276077534 | 0.04001105219794 | 8209.92274516707 | 15.4231662479618 |
| China | 2033 | 45 to 49 | 22521.3629563831 | 41.2224720634132 | 180109.2101 | 329.666854369911 | 86.5996068411349 | 0.158509495212387 | 19057.0480493654 | 34.8814870728521 |
| China | 2033 | 50 to 54 | 29002.9726643607 | 60.1640989415454 | 276148.307328252 | 572.845214071903 | 201.997528193096 | 0.419025987880574 | 31038.9917928021 | 64.387640359455 |
| China | 2033 | 55 to 59 | 39426.0815101027 | 82.8850442567774 | 434861.804507345 | 914.205483568977 | 413.896444481574 | 0.870130223562475 | 49958.8428733641 | 105.027959766052 |
| China | 2033 | 60 to 64 | 69003.276521656 | 117.067167833411 | 809880.095928492 | 1373.99807508045 | 959.51673763219 | 1.62786338019907 | 92505.1289177654 | 156.939119391942 |
| China | 2033 | 65 to 69 | 89075.2339511839 | 164.03372548696 | 1102535.89823263 | 2030.34067773904 | 1687.9657979313 | 3.10842089374676 | 125662.002250635 | 231.408950243329 |
| China | 2033 | 70 to 74 | 86469.2888051649 | 248.62863890548 | 1027093.60996869 | 2953.24374472916 | 2960.67032878661 | 8.51293498842893 | 136380.247987274 | 392.139635924038 |
| China | 2033 | 75 to 79 | 122350.109328225 | 365.388610010186 | 1451790.64330939 | 4335.65419840809 | 7333.72416163123 | 21.9015683135019 | 222940.788577405 | 665.794459033479 |
| China | 2033 | 80 to 84 | 129300.180921761 | 561.797367236826 | 1443049.48291393 | 6269.91698321026 | 16712.8470915719 | 72.6157800255372 | 312338.248787893 | 1357.08089969786 |
| China | 2033 | 85 to 89 | 81896.1629194069 | 811.090161395756 | 898473.475367073 | 8898.37777702031 | 19077.9212571426 | 188.945533953514 | 250895.435685751 | 2484.83948660787 |
| China | 2033 | 90 to 94 | 41310.029152265 | 1063.06837581654 | 452194.327039989 | 11636.7259637602 | 20431.4476456928 | 525.78093770472 | 205748.268517948 | 5294.7064460846 |
| China | 2033 | 95 plus | 14716.1763663284 | 1318.08097433717 | 147252.433645775 | 13188.9307644774 | 8794.87350595808 | 787.728765362551 | 80316.2064124915 | 7193.66640953452 |
| China | 2034 | 30 to 34 | 1898.35971223182 | 5.23098470866682 | 3646.0838432305 | 10.0468887469346 | 0.537205414780443 | 0.00148028494917108 | 276.095859892522 | 0.760790071511817 |
| China | 2034 | 35 to 39 | 5976.27186728485 | 15.4036069639303 | 24067.980136699 | 62.0342773344792 | 1.34287477260771 | 0.00346120719712576 | 1851.12644640354 | 4.77120600503935 |
| China | 2034 | 40 to 44 | 13811.5160822455 | 27.5758333680982 | 83191.2745697593 | 166.098255365635 | 19.9482896324002 | 0.0398284089600245 | 7724.43431070946 | 15.4224715191667 |
| China | 2034 | 45 to 49 | 23377.767739889 | 41.4153961925466 | 186600.526412817 | 330.576247360787 | 90.3912804367072 | 0.160134651575403 | 19826.9767383682 | 35.1249146649202 |
| China | 2034 | 50 to 54 | 29744.6506414178 | 60.6165521454848 | 282492.244108319 | 575.690266196744 | 210.544041461348 | 0.429067196721145 | 31977.2333119918 | 65.1663270109363 |
| China | 2034 | 55 to 59 | 38001.242963167 | 83.6775903156465 | 417701.933896508 | 919.767054265119 | 400.718492556676 | 0.882370028910931 | 48272.5317461949 | 106.294658279264 |
| China | 2034 | 60 to 64 | 68018.9228182738 | 118.1086317 | 796456.256047537 | 1382.97336581703 | 942.020966044998 | 1.63573315695533 | 91271.44297 | 158.484503994807 |
| China | 2034 | 65 to 69 | 90256.1865367891 | 165.141981331524 | 1116653.83495879 | 2043.14445183625 | 1713.86525061388 | 3.13586374609633 | 127565.345650651 | 233.406647658467 |
| China | 2034 | 70 to 74 | 98100.1510254502 | 250.053071899681 | 1165126.83952225 | 2969.85827575047 | 3349.96674049251 | 8.53892135196199 | 154770.088154518 | 394.502307862723 |
| China | 2034 | 75 to 79 | 119833.032031751 | 367.593745634113 | 1419992.78955793 | 4355.89803109321 | 7154.55526406277 | 21.9469517150017 | 217862.115532577 | 668.302802011825 |
| China | 2034 | 80 to 84 | 138822.222792912 | 565.870442090565 | 1546263.9190089 | 6302.9177161607 | 17864.6170457797 | 72.8201763528466 | 334410.443012301 | 1363.13179129368 |
| China | 2034 | 85 to 89 | 89778.5750244884 | 816.411560750226 | 983783.105651749 | 8946.14222274939 | 20869.6016223318 | 189.78006755037 | 274694.631283811 | 2497.96649807544 |
| China | 2034 | 90 to 94 | 43368.9217956034 | 1070.34046151907 | 474373.802462963 | 11707.4959127122 | 21315.5161816261 | 526.064713687943 | 215100.254840909 | 5308.65182962947 |
| China | 2034 | 95 plus | 15339.0550618268 | 1324.66763059245 | 153618.1251 | 13266.3294446244 | 9139.48571129371 | 789.278142181147 | 83477.5211378918 | 7209.04708195844 |
| China | 2035 | 30 to 34 | 1898.89729209149 | 5.25186325967095 | 3656.88296718041 | 10.1140010996055 | 0.532892544394987 | 0.00147384420785501 | 275.06481927519 | 0.760758796379282 |
| China | 2035 | 35 to 39 | 5855.89793876614 | 15.4530680185825 | 23616.3911163269 | 62.3210482986905 | 1.30679314161676 | 0.00344848279713629 | 1807.98400535187 | 4.77107014216482 |
| China | 2035 | 40 to 44 | 12800.7004659062 | 27.6545893712771 | 77174.6165220775 | 166.727776771921 | 18.3939950763375 | 0.0397383238587791 | 7138.56169779407 | 15.4221242017045 |
| China | 2035 | 45 to 49 | 24132.2885333229 | 41.524388917338 | 192751.375816903 | 331.666973180805 | 92.9042193860915 | 0.159860136452569 | 20412.784043968 | 35.124243701832 |
| China | 2035 | 50 to 54 | 30382.9821506809 | 60.7340825520669 | 287864.459503515 | 575.426854434064 | 216.818153884165 | 0.433408794155926 | 32805.8507597434 | 65.5772773834972 |
| China | 2035 | 55 to 59 | 37469.4935372061 | 84.0857207886501 | 410628.879051069 | 921.496983602387 | 402.448974280641 | 0.903140365359844 | 47904.5518663805 | 107.503155033097 |
| China | 2035 | 60 to 64 | 65755.6271937724 | 118.933695018375 | 767060.008502657 | 1387.39884334473 | 917.088587259385 | 1.65875893815929 | 88633.9987433322 | 160.314324791307 |
| China | 2035 | 65 to 69 | 92362.1817345756 | 166.214231396698 | 1139690.51082671 | 2050.97777823776 | 1751.72260332944 | 3.15238575642729 | 130917.711732732 | 235.5984497 |
| China | 2035 | 70 to 74 | 109996.710254976 | 251.171677288027 | 1305551.69684167 | 2981.15833393407 | 3770.14739249646 | 8.60893241262716 | 174144.361184728 | 397.649449584713 |
| China | 2035 | 75 to 79 | 113903.629577299 | 368.942860077967 | 1349240.09930004 | 4370.29533663648 | 6797.34793564343 | 22.0171472816804 | 207465.132733054 | 671.995964668041 |
| China | 2035 | 80 to 84 | 146703.507578702 | 568.162659254867 | 1631610.37462382 | 6319.00425977736 | 18843.0707807924 | 72.9766409818067 | 353135.10614488 | 1367.64406178828 |
| China | 2035 | 85 to 89 | 99607.2182723843 | 820.822479284258 | 1089157.70135918 | 8975.30460409458 | 23093.547788691 | 190.304512867199 | 304344.365265179 | 2507.97784323245 |
| China | 2035 | 90 to 94 | 45897.1126953947 | 1075.62626819732 | 501325.326071524 | 11748.8586529154 | 22536.8199590368 | 528.163845740259 | 227566.691165658 | 5333.16142148286 |
| China | 2035 | 95 plus | 16072.4765166014 | 1331.75209170533 | 160817.942062112 | 13325.2414774878 | 9533.28473131697 | 789.920076637854 | 87221.3871894297 | 7227.0918990601 |
| China | 2036 | 30 to 34 | 1907.64461064658 | 5.27274181067507 | 3683.46240058547 | 10.1811134522764 | 0.530897285529842 | 0.00146740346653893 | 275.226401781864 | 0.760727521246747 |
| China | 2036 | 35 to 39 | 5773.61790954041 | 15.5025290732346 | 23317.0745796342 | 62.6078192629019 | 1.27958193923786 | 0.00343575839714683 | 1776.84244099942 | 4.77093427929028 |
| China | 2036 | 40 to 44 | 11981.2805513047 | 27.733345374456 | 72301.2213170759 | 167.357298178206 | 17.1287187140669 | 0.0396482387575337 | 6662.47194324843 | 15.4217768842422 |
| China | 2036 | 45 to 49 | 24195.8792300655 | 41.6333816421293 | 193387.247932585 | 332.757699000823 | 92.7456350709189 | 0.159585621329735 | 20412.6037951982 | 35.1235727387437 |
| China | 2036 | 50 to 54 | 31200.4584655784 | 60.8516129586489 | 294903.655490318 | 575.163442671384 | 224.447837075374 | 0.437750391590707 | 33834.1558952446 | 65.988227756058 |
| China | 2036 | 55 to 59 | 37853.7810562379 | 84.4938512616537 | 413611.510255594 | 923.226912939656 | 413.917852004173 | 0.923910701808757 | 48703.5092324166 | 108.711651786929 |
| China | 2036 | 60 to 64 | 62767.9580120704 | 119.75875831345 | 729482.935219176 | 1391.82432087244 | 881.456973476975 | 1.68178471936326 | 84982.986342598 | 162.144145587806 |
| China | 2036 | 65 to 69 | 94373.9943521942 | 167.286481461871 | 1161469.98767348 | 2058.81110463926 | 1787.72654591836 | 3.16890776675825 | 134148.412277736 | 237.790251834532 |
| China | 2036 | 70 to 74 | 122110.484862792 | 252.290282676374 | 1448373.44235708 | 2992.45839211766 | 4200.67703114806 | 8.67894347329233 | 193988.708469584 | 400.796591306704 |
| China | 2036 | 75 to 79 | 107852.040329891 | 370.291974521822 | 1277095.04989738 | 4384.69264217975 | 6433.20718667362 | 22.0873428483591 | 196802.674712978 | 675.689127324257 |
| China | 2036 | 80 to 84 | 152148.159295543 | 570.454876419168 | 1689655.82476373 | 6335.09080339401 | 19505.6048465936 | 73.1331056107668 | 365972.960969415 | 1372.15633228289 |
| China | 2036 | 85 to 89 | 110288.985890477 | 825.233397818289 | 1203409.28388736 | 9004.46698543977 | 25503.4906879605 | 190.828958184028 | 336518.704653552 | 2517.98918838946 |
| China | 2036 | 90 to 94 | 49102.0199419733 | 1080.91207487558 | 535588.138407857 | 11790.2213931185 | 24087.975252805 | 530.262977792574 | 243380.081557188 | 5357.67101333624 |
| China | 2036 | 95 plus | 17021.1780189646 | 1338.83655281822 | 170158.231080033 | 13384.1535103512 | 10050.7389774697 | 790.562011094561 | 92110.3935786182 | 7245.13671616177 |
| China | 2037 | 30 to 34 | 1936.71187606953 | 5.2936203616792 | 3749.39252704321 | 10.2482258049474 | 0.534504491655019 | 0.00146096272522285 | 278.306594215904 | 0.760696246114211 |
| China | 2037 | 35 to 39 | 5701.88601574912 | 15.5519901278868 | 23059.2857591389 | 62.8945902271132 | 1.25500013305869 | 0.00342303399715737 | 1749.13619098441 | 4.77079841641575 |
| China | 2037 | 40 to 44 | 11511.5739393723 | 27.8121013776349 | 69530.6215172195 | 167.986819584492 | 16.3733262918998 | 0.0395581536562884 | 6383.01019755256 | 15.42142957 |
| China | 2037 | 45 to 49 | 23303.2477692011 | 41.7423743669207 | 186375.420156328 | 333.848424820841 | 88.9375900779356 | 0.159311106206901 | 19607.8372364948 | 35.1229017756555 |
| China | 2037 | 50 to 54 | 32087.0657463928 | 60.969143365231 | 302560.509647747 | 574.900030908704 | 232.665803303773 | 0.442091989025488 | 34944.8044784968 | 66.3991781286189 |
| China | 2037 | 55 to 59 | 39197.5927743267 | 84.9019817346574 | 427034.574419105 | 924.956842276924 | 436.140851870561 | 0.944681038257669 | 50747.9935351791 | 109.920148540762 |
| China | 2037 | 60 to 64 | 59722.6478466692 | 120.583821608525 | 691533.357489303 | 1396.24979840014 | 844.357027439587 | 1.70481050056722 | 81212.8801339888 | 163.973966384306 |
| China | 2037 | 65 to 69 | 96240.9253747572 | 168.358731527044 | 1181380.79718189 | 2066.64443104076 | 1820.92551234342 | 3.1854297770892 | 137183.826068043 | 239.982053922564 |
| China | 2037 | 70 to 74 | 129249.32559755 | 253.40888806472 | 1532044.73972604 | 3003.75845030125 | 4462.33943029193 | 8.7489545339575 | 206028.507808224 | 403.943733028694 |
| China | 2037 | 75 to 79 | 107175.120815624 | 371.641088965677 | 1268624.51603019 | 4399.08994772302 | 6389.86626375921 | 22.1575384150378 | 195922.574684355 | 679.382289980473 |
| China | 2037 | 80 to 84 | 156348.375602055 | 572.74709358347 | 1733742.99492796 | 6351.17734701067 | 20006.5707603352 | 73.2895702397268 | 375802.692319106 | 1376.66860277749 |
| China | 2037 | 85 to 89 | 122025.745337732 | 829.64431635232 | 1328684.27452547 | 9033.62936678496 | 28144.641293713 | 191.353403500857 | 371823.374475083 | 2528.00053354647 |
| China | 2037 | 90 to 94 | 53102.1072052769 | 1086.19788155384 | 578423.195004878 | 11831.5841333217 | 26026.1507678236 | 532.36210984489 | 263124.368357346 | 5382.18060518962 |
| China | 2037 | 95 plus | 17989.6341090436 | 1345.9210139311 | 179680.551773227 | 13443.0655432145 | 10575.2635843961 | 791.203945551267 | 97079.9749008604 | 7263.18153326344 |
| China | 2038 | 30 to 34 | 1979.46313687294 | 5.31449891268332 | 3842.09912596908 | 10.3153381576183 | 0.541758065288802 | 0.00145452198390677 | 283.320835003899 | 0.760664970981677 |
| China | 2038 | 35 to 39 | 5661.06893724648 | 15.6014511825389 | 22925.6905058592 | 63.1813611913246 | 1.23744884376705 | 0.00341030959716791 | 1731.06009665071 | 4.77066255354121 |
| China | 2038 | 40 to 44 | 11131.2358814199 | 27.8908573808139 | 67294.7496523146 | 168.616340990778 | 15.7516986613846 | 0.039468068555043 | 6154.55099569707 | 15.4210822493177 |
| China | 2038 | 45 to 49 | 22156.2422220493 | 41.851367091712 | 177317.814612463 | 334.939150640859 | 84.1944595622394 | 0.159036591084066 | 18593.8168174217 | 35.1222308125673 |
| China | 2038 | 50 to 54 | 33113.8451813555 | 61.086673771813 | 311498.840369324 | 574.636619146023 | 242.002580153971 | 0.446433586460269 | 36216.4137467797 | 66.8101285011798 |
| China | 2038 | 55 to 59 | 40645.0415163755 | 85.310112207661 | 441509.468575666 | 926.686771614193 | 459.978426841958 | 0.965451374706582 | 52945.9906307769 | 111.128645294594 |
| China | 2038 | 60 to 64 | 56671.4286931631 | 121.408884903601 | 653809.390351032 | 1400.67527592785 | 806.522115029753 | 1.72783628177118 | 77394.1504341641 | 165.803787180805 |
| China | 2038 | 65 to 69 | 96695.4912636049 | 169.430981592217 | 1183919.51688082 | 2074.47775744226 | 1827.37713125066 | 3.20195178742016 | 138210.377807442 | 242.173856010596 |
| China | 2038 | 70 to 74 | 130600.294187436 | 254.527493453066 | 1547053.02306786 | 3015.05850848485 | 4525.08876530847 | 8.81896559462267 | 208881.906163365 | 407.090874750685 |
| China | 2038 | 75 to 79 | 117391.385754379 | 372.990203409531 | 1389058.96169438 | 4413.48725326629 | 6995.74538537793 | 22.2277339817165 | 214984.665084562 | 683.075452636688 |
| China | 2038 | 80 to 84 | 158492.950120363 | 575.039310747772 | 1754952.08650707 | 6367.26389062732 | 20243.2747177648 | 73.4460348686869 | 380682.550154788 | 1381.18087327209 |
| China | 2038 | 85 to 89 | 133684.016136397 | 834.055234886352 | 1452602.11509004 | 9062.79174813015 | 30754.5596078608 | 191.877848817686 | 406797.543801996 | 2538.01187870349 |
| China | 2038 | 90 to 94 | 58098.323284821 | 1091.48368823209 | 631982.239623603 | 11872.9468735248 | 28448.7091742483 | 534.461241897206 | 287791.414892755 | 5406.690197 |
| China | 2038 | 95 plus | 18914.2317979394 | 1353.00547504398 | 188749.815366575 | 13501.9775760779 | 11069.5461319011 | 791.845880007974 | 101787.321266821 | 7281.2263503651 |
| China | 2039 | 30 to 34 | 2030.29578234709 | 5.33537746368745 | 3950.8817557772 | 10.3824505102892 | 0.551045030897566 | 0.0014480812425907 | 289.447446802812 | 0.760633695849141 |
| China | 2039 | 35 to 39 | 5655.18449801781 | 15.650912237191 | 22933.1039395407 | 63.4681321555359 | 1.2276582250663 | 0.00339758519717844 | 1723.74671709745 | 4.77052669066668 |
| China | 2039 | 40 to 44 | 10804.0068815871 | 27.9696133839928 | 65375.7145983469 | 169.245862397064 | 15.2107931696094 | 0.0393779834537977 | 5956.668905 | 15.4207349318554 |
| China | 2039 | 45 to 49 | 20902.0837061648 | 41.9603598165033 | 167389.522784668 | 336.029876460877 | 79.0855515924579 | 0.158762075961232 | 17495.4120287633 | 35.1215598494791 |
| China | 2039 | 50 to 54 | 34282.802968246 | 61.204204178395 | 321728.282612219 | 574.373207383343 | 252.496327987613 | 0.45077518389505 | 37653.0833670225 | 67.2210788737407 |
| China | 2039 | 55 to 59 | 41578.721376756 | 85.7182426806646 | 450340.302404441 | 928.416700951461 | 478.379356149487 | 0.986221711155495 | 54490.5563089219 | 112.337142048427 |
| China | 2039 | 60 to 64 | 54498.7550984821 | 122.233948198676 | 626472.784195778 | 1405.10075345556 | 780.632583561916 | 1.75086206297515 | 74740.4717106981 | 167.633607977304 |
| China | 2039 | 65 to 69 | 95124.7486805311 | 170.503231657391 | 1161733.51437316 | 2082.31108384376 | 1795.60532765233 | 3.21847379775112 | 136333.027748668 | 244.365658098629 |
| China | 2039 | 70 to 74 | 132207.840178814 | 255.646098841412 | 1565086.78 | 3026.35856666844 | 4596.95027744008 | 8.88897665528784 | 212155.328647274 | 410.238016472675 |
| China | 2039 | 75 to 79 | 133322.187116626 | 374.339317853386 | 1577005.74191792 | 4427.88455880956 | 7941.48141480773 | 22.2979295483951 | 244594.915540684 | 686.768615292904 |
| China | 2039 | 80 to 84 | 155056.924801976 | 577.331527912074 | 1714409.55571366 | 6383.35043424398 | 19767.8052870554 | 73.602499497647 | 372162.799368837 | 1385.69314376669 |
| China | 2039 | 85 to 89 | 143618.796407978 | 838.466153420383 | 1557338.36571113 | 9091.95412947534 | 32956.1137286345 | 192.402294134515 | 436444.604397702 | 2548.0232238605 |
| China | 2039 | 90 to 94 | 64078.8564493918 | 1096.76949491035 | 696094.611469921 | 11914.309613728 | 31348.5881384345 | 536.560373949522 | 317318.336474252 | 5431.19978889638 |
| China | 2039 | 95 plus | 19984.9303065999 | 1360.08993615687 | 199261.40656255 | 13560.8896089413 | 11644.6812228156 | 792.487814464681 | 107254.249658658 | 7299.27116746677 |
| China | 2040 | 30 to 34 | 2078.35122538827 | 5.35658802435373 | 4055.11194293231 | 10.451344222085 | 0.559398871437921 | 0.00144175308724423 | 295.113041479272 | 0.760602425859545 |
| China | 2040 | 35 to 39 | 5652.53742078586 | 15.7010065404526 | 22954.3762639182 | 63.7601814941515 | 1.21865524044716 | 0.00338504860320898 | 1717.39389343427 | 4.77039084326768 |
| China | 2040 | 40 to 44 | 10582.9066386248 | 28.0492665305869 | 64097.0752046557 | 169.884891517864 | 14.8235179265421 | 0.039288715231125 | 5818.06738538722 | 15.4203876456806 |
| China | 2040 | 45 to 49 | 19368.8516230534 | 42.0704968764951 | 155213.684498724 | 337.134950278661 | 72.9670148937289 | 0.158489446453298 | 16169.3190527379 | 35.1208889376593 |
| China | 2040 | 50 to 54 | 35386.546372938 | 61.3531136067285 | 332258.443670913 | 576.068933839948 | 259.630349899251 | 0.450146510067601 | 38770.3537407502 | 67.219951123809 |
| China | 2040 | 55 to 59 | 42473.9662627657 | 85.8732754624667 | 459015.213543639 | 928.030587730878 | 491.963570412691 | 0.994645118334113 | 55877.1476282876 | 112.971641514611 |
| China | 2040 | 60 to 64 | 53742.8147849153 | 122.786227682168 | 616066.800108227 | 1407.52803306361 | 781.980503704369 | 1.78659112953293 | 74132.3235751427 | 169.370145526142 |
| China | 2040 | 65 to 69 | 91960.5530513982 | 171.608898676655 | 1119142.45957559 | 2088.44769390096 | 1745.82379670557 | 3.25790666862005 | 132332.810570318 | 246.948143820601 |
| China | 2040 | 70 to 74 | 135377.777025093 | 257.165440608859 | 1598787.69033948 | 3037.0785357922 | 4699.33838283121 | 8.92692620862657 | 217766.776929228 | 413.672689636465 |
| China | 2040 | 75 to 79 | 149568.524132669 | 375.883025045233 | 1768095.90261969 | 4443.4297944751 | 8932.49450377492 | 22.448393301659 | 275221.796229129 | 691.664251719332 |
| China | 2040 | 80 to 84 | 147491.373836335 | 579.266262118802 | 1630287.59196982 | 6402.88699613679 | 18787.4818118337 | 73.7870566982597 | 354504.499466948 | 1392.30173920806 |
| China | 2040 | 85 to 89 | 152238.455380545 | 841.603818725105 | 1648551.1474096 | 9113.51167847487 | 34865.3481124176 | 192.742431859569 | 462259.429720063 | 2555.46011894056 |
| China | 2040 | 90 to 94 | 71671.409449043 | 1102.35198326468 | 777014.03222497 | 11950.9713291835 | 34963.6736918458 | 537.763598242451 | 354329.516537875 | 5449.81392562746 |
| China | 2040 | 95 plus | 21354.0081374854 | 1366.48948112489 | 212641.665981262 | 13607.403253827 | 12429.6350883847 | 795.399416032004 | 114558.909462796 | 7330.87407957148 |
| China | 2041 | 30 to 34 | 2118.46242943256 | 5.37779858502002 | 4144.2103974846 | 10.5202379338809 | 0.565453268734661 | 0.00143542493189776 | 299.60984836969 | 0.760571155869948 |
| China | 2041 | 35 to 39 | 5674.40401383576 | 15.7511008437141 | 23075.1005494092 | 64.052230832767 | 1.21496242528185 | 0.00337251200923951 | 1718.50554219388 | 4.77025499586868 |
| China | 2041 | 40 to 44 | 10430.8703529842 | 28.128919677181 | 63234.3128949749 | 170.523920638664 | 14.5360843696227 | 0.0391994470084524 | 5718.11657446163 | 15.4200403595057 |
| China | 2041 | 45 to 49 | 18125.9012431767 | 42.1806339364869 | 145348.817717947 | 338.240024096446 | 67.9890777194011 | 0.158216816945363 | 15091.8927518665 | 35.1202180258395 |
| China | 2041 | 50 to 54 | 35476.0329764277 | 61.5020230350619 | 333270.3077 | 577.764660296554 | 259.294065381504 | 0.449517836240153 | 38773.6382799776 | 67.2188233738772 |
| China | 2041 | 55 to 59 | 43620.6657392197 | 86.0283082442688 | 470362.260670671 | 927.644474510295 | 508.606036290823 | 1.00306852551273 | 57604.0096891724 | 113.606140980796 |
| China | 2041 | 60 to 64 | 54299.4163978559 | 123.33850716566 | 620728.69523463 | 1409.95531267166 | 802.271126930764 | 1.82232019609072 | 75329.2158811788 | 171.106683074981 |
| China | 2041 | 65 to 69 | 87784.9621737497 | 172.714565695919 | 1064606.23718572 | 2094.58430395817 | 1675.92597405865 | 3.29733953948899 | 126827.964898761 | 249.530629542574 |
| China | 2041 | 70 to 74 | 138390.721829915 | 258.684782376305 | 1630505.78860061 | 3047.79850491596 | 4796.01318800859 | 8.96487576196531 | 223143.350328701 | 417.107362800255 |
| China | 2041 | 75 to 79 | 165924.449479817 | 377.426732237079 | 1960255.89585319 | 4458.97503014064 | 9934.91609215373 | 22.5988570549229 | 306221.860029006 | 696.55988814576 |
| China | 2041 | 80 to 84 | 139850.240158689 | 581.20099632553 | 1545381.86044022 | 6422.42355802959 | 17799.2605554463 | 73.9716138988725 | 336609.791617801 | 1398.91033464943 |
| China | 2041 | 85 to 89 | 158305.32282194 | 844.741484029827 | 1711920.2861417 | 9135.0692274744 | 36183.8492452969 | 193.082569584624 | 480289.232663749 | 2562.89701402063 |
| China | 2041 | 90 to 94 | 79897.3427407497 | 1107.93447161901 | 864473.532101828 | 11987.633044639 | 38866.9348676155 | 538.966822535381 | 394349.018234751 | 5468.42806235855 |
| China | 2041 | 95 plus | 23127.1668357314 | 1372.88902609292 | 230008.695587294 | 13653.9168987128 | 13448.0440442916 | 798.311017599327 | 124025.48966052 | 7362.47699167618 |
| China | 2042 | 30 to 34 | 2167.29341301063 | 5.3990091456863 | 4250.73465258233 | 10.5891316456768 | 0.573674158868394 | 0.0014290967765513 | 305.299183706201 | 0.760539885880351 |
| China | 2042 | 35 to 39 | 5756.77832251948 | 15.8011951469757 | 23442.262045579 | 64.3442801713826 | 1.22412472315596 | 0.00335997541527004 | 1737.87604382579 | 4.77011914846969 |
| China | 2042 | 40 to 44 | 10297.9598170542 | 28.2085728237751 | 62485.5851376438 | 171.162949759464 | 14.2777535074128 | 0.0391101787857797 | 5629.1887098462 | 15.4196930733308 |
| China | 2042 | 45 to 49 | 17412.299212823 | 42.2907709964787 | 139717.915801991 | 339.34509791423 | 65.0300617790981 | 0.157944187437428 | 14459.7047573113 | 35.1195471140198 |
| China | 2042 | 50 to 54 | 34162.7366290357 | 61.6509324633953 | 321097.374988789 | 579.460386753159 | 248.743719168219 | 0.448889162412704 | 37247.4566183564 | 67.2176956239455 |
| China | 2042 | 55 to 59 | 44865.3977890253 | 86.183341026071 | 482712.955162389 | 927.258361289712 | 526.563340203524 | 1.01149193269135 | 59471.4908508401 | 114.240640446981 |
| China | 2042 | 60 to 64 | 56230.6545139298 | 123.890786649152 | 641041.999457771 | 1412.38259227971 | 843.317965634734 | 1.8580492626485 | 78448.8313202537 | 172.843220623819 |
| China | 2042 | 65 to 69 | 83528.9825728389 | 173.820232715183 | 1009497.44386035 | 2100.72091401538 | 1603.47964192992 | 3.33677241035792 | 121152.478525459 | 252.113115264546 |
| China | 2042 | 70 to 74 | 141140.857752901 | 260.204124143751 | 1659012.60135513 | 3058.51847403971 | 4883.34491769836 | 9.00282531530405 | 228111.925098946 | 420.542035964044 |
| China | 2042 | 75 to 79 | 175523.747871568 | 378.970439428926 | 2072416.43481505 | 4474.52026580618 | 10536.5633683797 | 22.7493208081867 | 324885.768989426 | 701.455524572188 |
| China | 2042 | 80 to 84 | 139807.537808844 | 583.135730532258 | 1544468.18446035 | 6441.9601199224 | 17779.0369410006 | 74.1561710994853 | 336974.962553501 | 1405.5189300908 |
| China | 2042 | 85 to 89 | 163273.255870995 | 847.879149334549 | 1763261.03521215 | 9156.62677647393 | 37246.7647147825 | 193.422707309679 | 494960.615960264 | 2570.33390910069 |
| China | 2042 | 90 to 94 | 88923.2262543816 | 1113.51695997334 | 960236.010708682 | 12024.2947600945 | 43136.8941979147 | 540.17004682831 | 438184.161064478 | 5487.04219908963 |
| China | 2042 | 95 plus | 25267.4218123226 | 1379.28857106095 | 250980.516201374 | 13700.4305435985 | 14677.7333683591 | 801.22261916665 | 135453.406750946 | 7394.07990378089 |
| China | 2043 | 30 to 34 | 2211.42058781423 | 5.42021970635258 | 4348.41721883292 | 10.6580253574726 | 0.580481971411762 | 0.00142276862120483 | 310.283438957247 | 0.760508615890755 |
| China | 2043 | 35 to 39 | 5879.77395826378 | 15.8512894502372 | 23975.7786395703 | 64.6363295099981 | 1.24167713107212 | 0.00334743882130057 | 1769.34650540804 | 4.76998330107069 |
| China | 2043 | 40 to 44 | 10221.0002528825 | 28.2882259703691 | 62074.8742399129 | 171.801978880264 | 14.0988953195929 | 0.0390209105631071 | 5571.26266436379 | 15.4193457871559 |
| China | 2043 | 45 to 49 | 16834.0784780782 | 42.4009080564705 | 135166.088923828 | 340.450171732015 | 62.5990220872381 | 0.157671557929494 | 13942.9541759432 | 35.1188762 |
| China | 2043 | 50 to 54 | 32477.0034710678 | 61.7998418917287 | 305408.695689105 | 581.156113209765 | 235.569493352932 | 0.448260488585255 | 35323.5969758975 | 67.2165678740138 |
| China | 2043 | 55 to 59 | 46304.9690123589 | 86.3383738078731 | 497099.827485316 | 926.872248069128 | 547.000668706151 | 1.01991533986997 | 61609.7982781842 | 114.875139913166 |
| China | 2043 | 60 to 64 | 58303.729348188 | 124.443066132644 | 662862.901190198 | 1414.80987188776 | 887.267909598241 | 1.89377832920629 | 81793.6369337061 | 174.579758172657 |
| China | 2043 | 65 to 69 | 79270.038212088 | 174.925899734447 | 954750.992815792 | 2106.85752407258 | 1529.9731032454 | 3.37620528122686 | 115418.757618522 | 254.695600986519 |
| China | 2043 | 70 to 74 | 141820.157286933 | 261.723465911198 | 1663128.97181422 | 3069.23844316347 | 4898.92684785743 | 9.04077486864279 | 229740.36112951 | 423.976709127834 |
| China | 2043 | 75 to 79 | 177564.129741606 | 380.514146620772 | 2095256.06428034 | 4490.06550147171 | 10686.0161521844 | 22.8997845614506 | 329613.577598971 | 706.351160998616 |
| China | 2043 | 80 to 84 | 154656.83041525 | 585.070464738985 | 1708024.34368998 | 6461.4966818152 | 19651.1396533813 | 74.3407283000981 | 373280.109667419 | 1412.12752553217 |
| China | 2043 | 85 to 89 | 166116.048309496 | 851.016814639271 | 1791555.33072525 | 9178.18432547346 | 37821.9531890433 | 193.762845034734 | 503173.488551584 | 2577.77080418076 |
| China | 2043 | 90 to 94 | 97846.1549196666 | 1119.09944832767 | 1054524.88386938 | 12060.9564755501 | 47333.8567315471 | 541.37327112124 | 481375.720070433 | 5505.65633582072 |
| China | 2043 | 95 plus | 27837.8506913699 | 1385.68811602898 | 276169.922621764 | 13746.9441884842 | 16154.6946341462 | 804.134220733973 | 149178.626213875 | 7425.6828158856 |
| China | 2044 | 30 to 34 | 2239.86829285968 | 5.44143026701887 | 4415.54604658928 | 10.7269190692685 | 0.583052604281137 | 0.00141644046585837 | 313.037016176869 | 0.760477345901158 |
| China | 2044 | 35 to 39 | 6026.65004380387 | 15.9013837534988 | 24607.9601183145 | 64.9283788486137 | 1.26393331335107 | 0.0033349022273311 | 1807.77986439597 | 4.7698474536717 |
| China | 2044 | 40 to 44 | 10207.2298415736 | 28.3678791169632 | 62047.1130577747 | 172.441008001065 | 14.0082457289212 | 0.0389316423404345 | 5548.0094573683 | 15.418998500981 |
| China | 2044 | 45 to 49 | 16336.3935320785 | 42.5110451164623 | 131254.851273616 | 341.555245549799 | 60.4861825715572 | 0.157398928421559 | 13495.4297216703 | 35.1182052903802 |
| China | 2044 | 50 to 54 | 30635.0581890381 | 61.9487513200621 | 288233.412995115 | 582.85183966637 | 221.364053353067 | 0.447631814757806 | 33239.5548824598 | 67.2154401240821 |
| China | 2044 | 55 to 59 | 47941.4763163164 | 86.4934065896752 | 513531.780543133 | 926.486134848545 | 569.986541525179 | 1.02833874704858 | 64024.5639402577 | 115.50963937935 |
| China | 2044 | 60 to 64 | 59636.4780988233 | 124.995345616137 | 676177.436282932 | 1417.23715149581 | 920.586482494965 | 1.92950739576407 | 84122.1955620012 | 176.316295721495 |
| China | 2044 | 65 to 69 | 76249.4233014868 | 176.031566753711 | 915259.615863328 | 2112.99413412979 | 1479.50986352492 | 3.41563815209579 | 111441.976580708 | 257.278086708491 |
| China | 2044 | 70 to 74 | 139551.114318731 | 263.242807678644 | 1632757.34778946 | 3079.95841228723 | 4812.84226092451 | 9.07872442198152 | 226580.681148631 | 427.411382291624 |
| China | 2044 | 75 to 79 | 180083.206270447 | 382.057853812619 | 2123722.4144283 | 4505.61073713725 | 10864.7488342946 | 23.0502483147144 | 335246.618939309 | 711.246797425043 |
| China | 2044 | 80 to 84 | 176561.401946412 | 587.005198945713 | 1949387.02012453 | 6481.03324370801 | 22415.966523116 | 74.5252855007109 | 426732.23160715 | 1418.73612097354 |
| China | 2044 | 85 to 89 | 162668.74952626 | 854.154479943992 | 1752037.29749567 | 9199.74187447299 | 36965.7833872422 | 194.102982759789 | 492337.760415414 | 2585.20769926082 |
| China | 2044 | 90 to 94 | 105404.235126191 | 1124.681937 | 1133778.49388558 | 12097.6181910056 | 50849.8079601948 | 542.576495414169 | 517729.933057653 | 5524.2704725518 |
| China | 2044 | 95 plus | 30893.4460277479 | 1392.087661 | 306106.761125978 | 13793.45783 | 17910.0980863001 | 807.045822301296 | 165493.352614135 | 7457.28572799031 |
| China | 2045 | 30 to 34 | 2256.7064585999 | 5.46264082768515 | 4459.92720322917 | 10.7958127810644 | 0.582540507213235 | 0.0014101123105119 | 314.15273767025 | 0.760446075911561 |
| China | 2045 | 35 to 39 | 6164.69881421095 | 15.9514780567603 | 25205.4571292687 | 65.2204281872292 | 1.28398029370573 | 0.00332236563336163 | 1843.32983932207 | 4.7697116062727 |
| China | 2045 | 40 to 44 | 10198.9654579309 | 28.4475322635573 | 62052.3883656738 | 173.080037121865 | 13.9257081514438 | 0.0388423741177618 | 5527.87108880942 | 15.4186512148061 |
| China | 2045 | 45 to 49 | 15999.0115018508 | 42.6211821764541 | 128626.802703246 | 342.660319367584 | 58.9815987072995 | 0.157126298913624 | 13182.3147024209 | 35.1175343785605 |
| China | 2045 | 50 to 54 | 28384.9599556002 | 62.0976607483956 | 267197.814806139 | 584.547566122976 | 204.325993965297 | 0.447003140930357 | 30723.7912377039 | 67.2143123741504 |
| China | 2045 | 55 to 59 | 49461.8093873639 | 86.6484393714773 | 528648.67590996 | 926.100021627962 | 591.818299606893 | 1.0367621542272 | 66298.9350842066 | 116.144138845535 |
| China | 2045 | 60 to 64 | 61096.2012415644 | 125.547625099629 | 690862.162540968 | 1419.66443110386 | 956.358053718623 | 1.96523646232186 | 86647.2123585167 | 178.052833270333 |
| China | 2045 | 65 to 69 | 75379.7259788782 | 177.137233772975 | 901783.839613076 | 2119.130744 | 1470.28550351213 | 3.45507102296473 | 110582.164603675 | 259.860572430464 |
| China | 2045 | 70 to 74 | 134912.66155789 | 264.76214944609 | 1574891.45381217 | 3090.67838141099 | 4645.50825387691 | 9.11667397532026 | 219542.665689997 | 430.846055455413 |
| China | 2045 | 75 to 79 | 184391.209470183 | 383.601561004465 | 2173248.24186193 | 4521.15597280279 | 11152.2157198265 | 23.2007120679783 | 344238.352901947 | 716.142433851471 |
| China | 2045 | 80 to 84 | 198084.392812254 | 588.939933152441 | 2186405.35374057 | 6500.56980560082 | 25127.9510787735 | 74.7098427013237 | 479401.25436548 | 1425.34471641491 |
| China | 2045 | 85 to 89 | 155032.111442276 | 857.292145248714 | 1667573.33283117 | 9221.29942347252 | 35162.9577982949 | 194.443120484844 | 468852.03358939 | 2592.64459434088 |
| China | 2045 | 90 to 94 | 112372.403691442 | 1130.26442503634 | 1206406.36823544 | 12134.2799064611 | 54063.3083981041 | 543.779719707098 | 551081.015320281 | 5542.88460928289 |
| China | 2045 | 95 plus | 34806.1229618121 | 1398.48720596503 | 344454.884539134 | 13839.9714782557 | 20158.552447786 | 809.957423868619 | 186386.530919027 | 7488.88864009501 |
| United States of America | 2022 | 30 to 34 | 419.335208961821 | 3.58012806409557 | 731.074180934435 | 6.24163947162699 | 1.83716708131777 | 0.0156850493011121 | 160.845808568348 | 1.37324169528563 |
| United States of America | 2022 | 35 to 39 | 1193.85917387201 | 10.6624633434321 | 4675.11494537902 | 41.7538708269619 | 3.38659495847915 | 0.0302459832734904 | 585.089722731075 | 5.22548878273959 |
| United States of America | 2022 | 40 to 44 | 1960.3511352584 | 17.9927714621364 | 12366.8850862222 | 113.507490088284 | 11.5327054741229 | 0.105851105041276 | 1547.09559031709 | 14.1997710950824 |
| United States of America | 2022 | 45 to 49 | 2572.42528994696 | 25.4174339705246 | 22463.6641369517 | 221.957349808509 | 26.5337395871442 | 0.262172657290754 | 2916.62632645711 | 28.8183907066728 |
| United States of America | 2022 | 50 to 54 | 5392.94096112387 | 50.3184001944074 | 42485.0435975447 | 396.403268908139 | 63.042756804505 | 0.588215352089949 | 5755.81133732857 | 53.7041329402667 |
| United States of America | 2022 | 55 to 59 | 10072.1794989031 | 92.6098227820806 | 81729.1023625003 | 751.467712301388 | 138.091379741708 | 1.26969720972558 | 11028.3681745562 | 101.401610478885 |
| United States of America | 2022 | 60 to 64 | 23081.3068407399 | 210.00585618161 | 158331.511900746 | 1440.58328008342 | 290.156773923838 | 2.63999877282581 | 20613.6834090167 | 187.554121750432 |
| United States of America | 2022 | 65 to 69 | 39485.5824506774 | 399.104451029767 | 291590.352159975 | 2947.27848 | 560.16798853548 | 5.66195364670544 | 35745.5161811943 | 361.301359302259 |
| United States of America | 2022 | 70 to 74 | 50079.7576610793 | 621.903601259998 | 437203.889874922 | 5429.31288602073 | 1078.31749323531 | 13.3908302209272 | 53875.5587366725 | 669.040857285751 |
| United States of America | 2022 | 75 to 79 | 51545.6920827079 | 866.981509846367 | 538710.583674665 | 9060.93402441989 | 1803.18108544391 | 30.328910076057 | 67822.1033118305 | 1140.74536890296 |
| United States of America | 2022 | 80 to 84 | 31486.0516206123 | 831.438704551974 | 503695.506006995 | 13300.8718923952 | 2958.74594693631 | 78.1303393280559 | 72513.0349206394 | 1914.82071312109 |
| United States of America | 2022 | 85 to 89 | 13427.988216577 | 557.031626820716 | 393459.768652679 | 16321.8444554886 | 4166.44233498497 | 172.836028337162 | 68151.3618991787 | 2827.11478268068 |
| United States of America | 2022 | 90 to 94 | 4617.06153988772 | 380.099229511815 | 214703.998099267 | 17675.4898208747 | 4916.1059274173 | 404.718035284239 | 56497.6333422428 | 4651.16323815552 |
| United States of America | 2022 | 95 plus | 1235.38503698075 | 289.743472127802 | 73039.2409298411 | 17130.4027773496 | 3059.61098653964 | 717.591911884372 | 29375.3313652549 | 6889.6014197121 |
| United States of America | 2023 | 30 to 34 | 426.510256262462 | 3.61533748014123 | 749.944171381768 | 6.35694272529172 | 1.95123859287996 | 0.0165397804952102 | 166.443673642421 | 1.41086888959018 |
| United States of America | 2023 | 35 to 39 | 1214.26359121955 | 10.746528591239 | 4762.94806561169 | 42.153250691042 | 3.55113355381956 | 0.0314283970493603 | 609.776361253637 | 5.3966693458151 |
| United States of America | 2023 | 40 to 44 | 2001.70597196916 | 18.1511027595738 | 12639.8009260993 | 114.615397407487 | 11.9605118947637 | 0.108455729012685 | 1588.31406143279 | 14.4025407064064 |
| United States of America | 2023 | 45 to 49 | 2609.8338841832 | 25.6224983013775 | 22809.2421224142 | 223.933703627338 | 27.4615002547169 | 0.269608057391736 | 2974.37038788009 | 29.2013988602868 |
| United States of America | 2023 | 50 to 54 | 5388.72483116302 | 50.6369914844229 | 42498.3028672205 | 399.349803119455 | 63.4802073677164 | 0.596513427688736 | 5761.59452630712 | 54.1407887962883 |
| United States of America | 2023 | 55 to 59 | 9945.01552906914 | 93.1346918372102 | 80757.6221140003 | 756.292056770402 | 137.387938564187 | 1.28663281449122 | 10913.7096963116 | 102.206475836634 |
| United States of America | 2023 | 60 to 64 | 23186.9007672045 | 210.830486427981 | 159259.982130497 | 1448.09605381049 | 294.932006333312 | 2.68171494684536 | 20795.0367429104 | 189.082092333641 |
| United States of America | 2023 | 65 to 69 | 40469.5553892143 | 400.8065678 | 298857.496801798 | 2959.85578333783 | 579.733050329768 | 5.74162013726779 | 36755.2434570923 | 364.020381214041 |
| United States of America | 2023 | 70 to 74 | 51125.4447128371 | 624.310757306932 | 446324.838576915 | 5450.22932400787 | 1103.4324433584 | 13.4744010192863 | 55015.6915215814 | 671.815927089214 |
| United States of America | 2023 | 75 to 79 | 54815.904742133 | 870.74540298228 | 572463.634999726 | 9093.53007845913 | 1912.90350899439 | 30.3862892465455 | 72012.9233183439 | 1143.91839794932 |
| United States of America | 2023 | 80 to 84 | 33345.9780336785 | 837.080014009376 | 532021.004729085 | 13355.258305578 | 3121.81598251919 | 78.366565339382 | 76579.7257742394 | 1922.37150336967 |
| United States of America | 2023 | 85 to 89 | 13709.9986506947 | 561.801841312672 | 400270.379991348 | 16402.0903452581 | 4225.90314660408 | 173.167060731321 | 69260.5805772888 | 2838.12731788517 |
| United States of America | 2023 | 90 to 94 | 4908.05611874554 | 382.887295959254 | 227798.775087714 | 17771.0390643329 | 5228.58537053234 | 407.892425387941 | 59995.7875178217 | 4680.39164505644 |
| United States of America | 2023 | 95 plus | 1272.60707379322 | 290.205686365521 | 75531.6362207265 | 17224.2562399181 | 3183.10322121109 | 725.875782169323 | 30471.9303393625 | 6948.82783627675 |
| United States of America | 2024 | 30 to 34 | 430.65841235178 | 3.65054689618689 | 763.536877326252 | 6.47224597895645 | 2.05204672088357 | 0.0173945116893083 | 170.880430118418 | 1.44849608389472 |
| United States of America | 2024 | 35 to 39 | 1239.01072918209 | 10.830593839046 | 4867.984766 | 42.5526305551221 | 3.73064903921713 | 0.0326108108252302 | 636.957296904663 | 5.56784990889061 |
| United States of America | 2024 | 40 to 44 | 2034.82151724672 | 18.3094340570113 | 12860.9256720634 | 115.72330472669 | 12.3427078773366 | 0.111060352984093 | 1623.16320690441 | 14.6053103177304 |
| United States of America | 2024 | 45 to 49 | 2665.10615529776 | 25.8275626322303 | 23311.3086672809 | 225.910057446167 | 28.5876849613144 | 0.277043457492718 | 3052.76910393355 | 29.5844070139008 |
| United States of America | 2024 | 50 to 54 | 5355.98614633407 | 50.9555827744385 | 42285.7220376142 | 402.296337330771 | 63.5722693446745 | 0.604811503287524 | 5736.68323555558 | 54.5774446523098 |
| United States of America | 2024 | 55 to 59 | 9852.16628471634 | 93.6595608923398 | 80062.7856418758 | 761.116401239417 | 137.123991482153 | 1.30356841925686 | 10835.8917444136 | 103.011341194383 |
| United States of America | 2024 | 60 to 64 | 23273.0738413735 | 211.655116674351 | 160055.151322225 | 1455.60882753755 | 299.461759175437 | 2.72343112086491 | 20959.0117114814 | 190.61006291685 |
| United States of America | 2024 | 65 to 69 | 41256.741694076 | 402.508684660233 | 304671.448682675 | 2972.43308674567 | 596.676116278827 | 5.82128662783013 | 37590.4257483877 | 366.739403125823 |
| United States of America | 2024 | 70 to 74 | 53429.1874348717 | 626.717913353866 | 466428.142825529 | 5471.145762 | 1155.84922984748 | 13.5579718176454 | 57510.4812657634 | 674.590996892677 |
| United States of America | 2024 | 75 to 79 | 57157.5952807525 | 874.509296118192 | 596480.136092163 | 9126.12613249837 | 1989.78659914334 | 30.4436684170339 | 74973.4597736953 | 1147.09142699568 |
| United States of America | 2024 | 80 to 84 | 35199.9724756264 | 842.721323466777 | 560112.948212252 | 13409.6447187607 | 3283.19221907218 | 78.602791350708 | 80611.7155502808 | 1929.92229361824 |
| United States of America | 2024 | 85 to 89 | 14110.6243585488 | 566.572055804627 | 410496.869340784 | 16482.3362350276 | 4321.0151187943 | 173.49809312548 | 70958.5688175597 | 2849.13985308965 |
| United States of America | 2024 | 90 to 94 | 5121.95008018123 | 385.675362406694 | 237276.690023971 | 17866.5883077911 | 5459.16051113231 | 411.066815491643 | 62545.967811418 | 4709.62005195735 |
| United States of America | 2024 | 95 plus | 1334.04648992963 | 290.667900603241 | 79483.0231094356 | 17318.1097024866 | 3369.49179930761 | 734.159652454274 | 32164.0957182984 | 7008.0542528414 |
| United States of America | 2025 | 30 to 34 | 430.994448131134 | 3.67787892139592 | 769.265256091756 | 6.5645032844629 | 2.12939305899959 | 0.0181711153844806 | 173.383255796056 | 1.47956110474372 |
| United States of America | 2025 | 35 to 39 | 1264.47315527708 | 10.895644952095 | 4986.24618085129 | 42.9652205770953 | 3.93215557292336 | 0.0338823887562774 | 656.650075540713 | 5.65818740476955 |
| United States of America | 2025 | 40 to 44 | 2052.8187701025 | 18.3804323436288 | 12975.4096906608 | 116.178614217439 | 12.7079896950582 | 0.113784201613611 | 1666.66544017126 | 14.9229107842786 |
| United States of America | 2025 | 45 to 49 | 2726.48185879441 | 25.9529563369127 | 23839.1937196302 | 226.921573571864 | 29.5165120499494 | 0.280963082875241 | 3134.03637877756 | 29.8324043618158 |
| United States of America | 2025 | 50 to 54 | 5281.15646114485 | 51.1895271825672 | 41686.8420121225 | 404.064857391545 | 63.2516496813104 | 0.613089588336446 | 5666.36242627021 | 54.9232759112306 |
| United States of America | 2025 | 55 to 59 | 9847.15686707068 | 94.0219516044412 | 80014.8180595545 | 763.992028642433 | 137.333722974394 | 1.31128048729839 | 10828.6694409854 | 103.39356302178 |
| United States of America | 2025 | 60 to 64 | 23206.9810671685 | 212.360990618284 | 159625.996207144 | 1460.69558056116 | 300.028978292629 | 2.74548640600878 | 20937.4214171129 | 191.592846146665 |
| United States of America | 2025 | 65 to 69 | 41847.5842375492 | 403.543093393259 | 309146.72924055 | 2981.15242977873 | 608.640105751765 | 5.86921600167024 | 38202.8322808852 | 368.396154662119 |
| United States of America | 2025 | 70 to 74 | 56379.0194429305 | 628.667274042486 | 491932.640641618 | 5485.40849522467 | 1224.52329452701 | 13.6543297343273 | 60753.0711809351 | 677.441147902348 |
| United States of America | 2025 | 75 to 79 | 59024.1155924238 | 877.394335276297 | 615524.789050713 | 9149.78492629127 | 2053.80074113548 | 30.5297778369394 | 77349.024406229 | 1149.79437086025 |
| United States of America | 2025 | 80 to 84 | 36635.9972970928 | 846.02933107638 | 582261.473103667 | 13446.0727411543 | 3404.24233710694 | 78.61363358 | 83683.6018953934 | 1932.49227418302 |
| United States of America | 2025 | 85 to 89 | 14646.6468083247 | 569.636473493967 | 425160.470013412 | 16535.3144632276 | 4465.35783800717 | 173.66641832896 | 73413.301493373 | 2855.1855395643 |
| United States of America | 2025 | 90 to 94 | 5246.10019378576 | 388.565187400667 | 242134.059346368 | 17934.2488078008 | 5553.933092 | 411.365580729875 | 63728.4729976283 | 4720.20455926752 |
| United States of America | 2025 | 95 plus | 1430.35513157066 | 293.460960214247 | 84797.6858933711 | 17397.6446666703 | 3595.24680155172 | 737.624213248364 | 34312.5304775328 | 7039.78188288128 |
| United States of America | 2026 | 30 to 34 | 429.721880654175 | 3.70521094660496 | 772.035849242895 | 6.65676058996936 | 2.19751306858144 | 0.0189477190796528 | 175.198958712417 | 1.51062612559273 |
| United States of America | 2026 | 35 to 39 | 1290.46949838845 | 10.9606960651441 | 5107.13381269488 | 43.3778105990685 | 4.13889058575586 | 0.0351539666873246 | 676.808845069992 | 5.74852490064849 |
| United States of America | 2026 | 40 to 44 | 2070.88450787008 | 18.4514306302462 | 13090.3337816773 | 116.633923708188 | 13.0762064538854 | 0.116508050243129 | 1710.50902630934 | 15.2405112508268 |
| United States of America | 2026 | 45 to 49 | 2795.96135274108 | 26.0783500415952 | 24437.5932061944 | 227.93308969756 | 30.5433833460501 | 0.284882708257765 | 3225.0368800629 | 30.0804017097309 |
| United States of America | 2026 | 50 to 54 | 5202.57895710906 | 51.4234715906959 | 41058.6863219079 | 405.833377452318 | 62.8645690806965 | 0.621367673385369 | 5591.64686955246 | 55.2691071701513 |
| United States of America | 2026 | 55 to 59 | 9929.00062621818 | 94.3843423165426 | 80672.5909215474 | 766.867656045449 | 138.754772100074 | 1.31899255533993 | 10916.9572777026 | 103.775784849176 |
| United States of America | 2026 | 60 to 64 | 22932.9832200523 | 213.066864562218 | 157766.256754262 | 1465.78233358477 | 297.878261335496 | 2.76754169115266 | 20727.4542015527 | 192.575629376479 |
| United States of America | 2026 | 65 to 69 | 42382.1676050174 | 404.577502126285 | 313208.831254453 | 2989.87177281179 | 619.860090415642 | 5.91714537551036 | 38765.487973994 | 370.052906198416 |
| United States of America | 2026 | 70 to 74 | 57836.9478988663 | 630.616634731106 | 504401.85174728 | 5499.67122845435 | 1261.14308035041 | 13.7506876510091 | 62392.855253499 | 680.29129891202 |
| United States of America | 2026 | 75 to 79 | 61810.9641575852 | 880.279374434402 | 644135.734008387 | 9173.44372008417 | 2149.76922650426 | 30.6158872568448 | 80925.4110468506 | 1152.49731472481 |
| United States of America | 2026 | 80 to 84 | 39092.432700953 | 849.337338685983 | 620558.792993805 | 13482.5007635479 | 3618.84717541631 | 78.624475805632 | 89065.0716980317 | 1935.0622547478 |
| United States of America | 2026 | 85 to 89 | 15266.7281906477 | 572.700891183307 | 442201.085358314 | 16588.2926914275 | 4633.98576893653 | 173.83474353244 | 76273.0425096352 | 2861.23122603895 |
| United States of America | 2026 | 90 to 94 | 5363.52868142541 | 391.45501239464 | 246653.520419152 | 18001.9093078105 | 5640.42726956894 | 411.664345968107 | 64819.0010114956 | 4730.7890665777 |
| United States of America | 2026 | 95 plus | 1524.51133585996 | 296.254019825253 | 89936.8672931895 | 17477.179630854 | 3813.61318767151 | 741.088774042454 | 36389.7050931118 | 7071.50951292116 |
| United States of America | 2027 | 30 to 34 | 428.780576891277 | 3.732542972 | 775.301934505333 | 6.74901789547581 | 2.26585643140473 | 0.0197243227748251 | 177.103712978338 | 1.54169114644173 |
| United States of America | 2027 | 35 to 39 | 1314.47910412437 | 11.0257471781932 | 5220.649961 | 43.7904006210417 | 4.34261882513491 | 0.0364255446183719 | 696.103627994724 | 5.83886239652744 |
| United States of America | 2027 | 40 to 44 | 2092.51324645588 | 18.5224289168637 | 13227.7884604576 | 117.089233198937 | 13.4698493869749 | 0.119231898872647 | 1757.62881879965 | 15.558111717375 |
| United States of America | 2027 | 45 to 49 | 2857.7737420331 | 26.2037437462776 | 24968.6414749329 | 228.944605823257 | 31.4967103062264 | 0.288802333640288 | 3307.60761912711 | 30.328399057646 |
| United States of America | 2027 | 50 to 54 | 5191.46131073412 | 51.6574159988247 | 40963.130660062 | 407.601897513092 | 63.2780701700961 | 0.629645758434292 | 5589.18396460166 | 55.6149384290721 |
| United States of America | 2027 | 55 to 59 | 9979.95763010616 | 94.746733028644 | 81079.3692754772 | 769.743283448466 | 139.745778094639 | 1.32670462338146 | 10971.2753172635 | 104.158006676573 |
| United States of America | 2027 | 60 to 64 | 22592.5291144748 | 213.772738506151 | 155448.505244389 | 1470.86908660837 | 294.817998520504 | 2.78959697629653 | 20456.1821246137 | 193.558412606294 |
| United States of America | 2027 | 65 to 69 | 42746.1737763009 | 405.611910859311 | 316012.162094607 | 2998.59111584485 | 628.640616800828 | 5.96507474935047 | 39173.3210945292 | 371.709657734712 |
| United States of America | 2027 | 70 to 74 | 58665.9605769925 | 632.565995419727 | 511377.840039688 | 5513.93396168403 | 1284.21419308662 | 13.847045567691 | 63356.4713560409 | 683.141449921691 |
| United States of America | 2027 | 75 to 79 | 64011.7566971849 | 883.164413592507 | 666605.990205811 | 9197.10251387708 | 2225.28071913085 | 30.7019966767502 | 83728.9147425581 | 1155.20025858938 |
| United States of America | 2027 | 80 to 84 | 42536.4470307626 | 852.645346295586 | 674427.182080113 | 13518.9287859415 | 3922.92886461414 | 78.635318033094 | 96663.8606550242 | 1937.63223531257 |
| United States of America | 2027 | 85 to 89 | 16023.4506658233 | 575.765308872648 | 463123.740677177 | 16641.2709196275 | 4842.47582239896 | 174.00306873592 | 79795.8290381782 | 2867.2769125136 |
| United States of America | 2027 | 90 to 94 | 5477.52866029532 | 394.344837388613 | 250989.938544584 | 18069.5698078202 | 5722.25000727828 | 411.963111206339 | 65858.6272160173 | 4741.37357388787 |
| United States of America | 2027 | 95 plus | 1625.44049556784 | 299.047079436259 | 95427.7665098678 | 17556.7145950377 | 4046.94519617055 | 744.553334836545 | 38608.9351668504 | 7103.23714296103 |
| United States of America | 2028 | 30 to 34 | 429.718369334761 | 3.75987499702303 | 781.893447485385 | 6.84127520098227 | 2.34306318681189 | 0.0205009264699974 | 179.751246013359 | 1.57275616729074 |
| United States of America | 2028 | 35 to 39 | 1331.6874561311 | 11.0907982912423 | 5307.51408663389 | 44.2029906430149 | 4.52634552653787 | 0.0376971225494191 | 711.927213377085 | 5.92919989240638 |
| United States of America | 2028 | 40 to 44 | 2119.75575406055 | 18.5934272034811 | 13400.7419932904 | 117.544542689686 | 13.9036442652202 | 0.121955747502165 | 1809.92088674108 | 15.8757121839232 |
| United States of America | 2028 | 45 to 49 | 2906.69069859545 | 26.3291374509601 | 25386.7534399511 | 229.956121948953 | 32.3159920126901 | 0.292721959022812 | 3375.58065448023 | 30.576396405561 |
| United States of America | 2028 | 50 to 54 | 5250.13122137209 | 51.8913604069534 | 41418.2321210196 | 409.370417573865 | 64.5422255509054 | 0.637923843483215 | 5661.85549592134 | 55.9607696879929 |
| United States of America | 2028 | 55 to 59 | 9953.04425542566 | 95.1091237407453 | 80853.5491636453 | 772.618910851482 | 139.644945327384 | 1.334416691 | 10939.9969198388 | 104.54022850397 |
| United States of America | 2028 | 60 to 64 | 22277.4194774567 | 214.478612450084 | 153304.271200168 | 1475.95583963198 | 292.040106644318 | 2.81165226144041 | 20206.5640754539 | 194.541195836109 |
| United States of America | 2028 | 65 to 69 | 42933.6736914638 | 406.646319592338 | 317511.507444179 | 3007.31045887791 | 634.852313896008 | 6.01300412319058 | 39419.9844205281 | 373.366409271009 |
| United States of America | 2028 | 70 to 74 | 60158.261319254 | 634.515356108347 | 524127.112441495 | 5528.1966949137 | 1321.97101680462 | 13.9434034843729 | 65038.7127661502 | 685.991600931362 |
| United States of America | 2028 | 75 to 79 | 65505.9993221284 | 886.049452750613 | 681694.663988178 | 9220.76130766998 | 2276.17730684925 | 30.7881060966556 | 85604.2584977331 | 1157.90320245394 |
| United States of America | 2028 | 80 to 84 | 45389.5800462197 | 855.953353905189 | 718814.816368076 | 13555.3568083351 | 4170.45645091279 | 78.646160260556 | 102885.237123766 | 1940.20221587735 |
| United States of America | 2028 | 85 to 89 | 17045.1925622849 | 578.829726561988 | 491606.906745495 | 16694.2491478274 | 5128.94347388137 | 174.171393939399 | 84612.6844317741 | 2873.32259898824 |
| United States of America | 2028 | 90 to 94 | 5619.50795782163 | 397.234662382586 | 256579.598155837 | 18137.2303078299 | 5832.09149345446 | 412.26187644457 | 67223.9076327339 | 4751.95808119805 |
| United States of America | 2028 | 95 plus | 1721.65389550613 | 301.840139047265 | 100594.69841086 | 17636.2495592214 | 4266.58935417175 | 748.017895630635 | 40696.840170129 | 7134.96477300091 |
| United States of America | 2029 | 30 to 34 | 432.869809506379 | 3.78720702223207 | 792.488205073398 | 6.93353250648873 | 2.43197701506555 | 0.0212775301651697 | 183.313393779877 | 1.60382118813974 |
| United States of America | 2029 | 35 to 39 | 1339.5889301011 | 11.1558494042914 | 5357.41706461724 | 44.6155806649881 | 4.67934245903116 | 0.0389687004804663 | 722.823100011981 | 6.01953738828532 |
| United States of America | 2029 | 40 to 44 | 2154.16586659502 | 18.6644254900986 | 13619.0237393161 | 117.999852180434 | 14.3899703952959 | 0.124679596131683 | 1868.96089554175 | 16.1933126504714 |
| United States of America | 2029 | 45 to 49 | 2943.29648094097 | 26.4545311556425 | 25697.1568445797 | 230.96763807465 | 33.003954080462 | 0.296641584405335 | 3429.48166905594 | 30.8243937534761 |
| United States of America | 2029 | 50 to 54 | 5343.30609499963 | 52.1253048150822 | 42145.3879099273 | 411.138937634639 | 66.2414294856502 | 0.646201928532138 | 5771.92603660905 | 56.3066009469136 |
| United States of America | 2029 | 55 to 59 | 9870.78364912237 | 95.4715144528467 | 80178.2484760611 | 775.494538254498 | 138.762464279149 | 1.34212875946453 | 10847.9143029435 | 104.922450331367 |
| United States of America | 2029 | 60 to 64 | 22028.3304317572 | 215.184486394017 | 151613.604499282 | 1481.04259265559 | 290.085253027135 | 2.83370754658428 | 20015.6939302289 | 195.523979065924 |
| United States of America | 2029 | 65 to 69 | 43059.4138284381 | 407.680728325364 | 318554.364570651 | 3016.02980191097 | 640.158402157786 | 6.06093349703069 | 39610.1075044267 | 375.023160807305 |
| United States of America | 2029 | 70 to 74 | 61310.7798266424 | 636.464716796967 | 533906.280629532 | 5542.45942814338 | 1352.45316411349 | 14.0397614010547 | 66356.2706212476 | 688.841751941033 |
| United States of America | 2029 | 75 to 79 | 68509.3545823274 | 888.934491908718 | 712458.85990905 | 9244.42010146289 | 2379.44707684093 | 30.874215516561 | 89446.8363330515 | 1160.60614631851 |
| United States of America | 2029 | 80 to 84 | 47367.5558887282 | 859.261361514791 | 749259.371400269 | 13591.7848307287 | 4336.03805345423 | 78.657002488018 | 107097.065823849 | 1942.77219644213 |
| United States of America | 2029 | 85 to 89 | 18031.5437169925 | 581.894144251328 | 518957.555343303 | 16747.2273760274 | 5402.38168469222 | 174.339719142879 | 89224.9142383898 | 2879.36828546289 |
| United States of America | 2029 | 90 to 94 | 5816.65768793153 | 400.12448737656 | 264646.682260461 | 18204.8908078396 | 5997.4435554199 | 412.560641682802 | 69233.6531142546 | 4762.54258850822 |
| United States of America | 2029 | 95 plus | 1811.39469420188 | 304.633198658271 | 105340.712143845 | 17715.7845234051 | 4468.42740826988 | 751.482456424725 | 42614.228032341 | 7166.69240304079 |
| United States of America | 2030 | 30 to 34 | 437.444951948738 | 3.80587865130822 | 804.334249383131 | 6.99790575844159 | 2.51350439262342 | 0.0218681062959801 | 186.887952588914 | 1.62597114397197 |
| United States of America | 2030 | 35 to 39 | 1336.35849781264 | 11.200078193562 | 5357.2589073447 | 44.8994179059201 | 4.76328406470441 | 0.0399212891377273 | 725.798831702125 | 6.0829513047325 |
| United States of America | 2030 | 40 to 44 | 2192.96660469598 | 18.7311338794741 | 13887.144986448 | 118.616476597411 | 14.8770388043588 | 0.127071613848496 | 1912.1738958849 | 16.3327545289368 |
| United States of America | 2030 | 45 to 49 | 2963.88502126729 | 26.5055212254261 | 25856.613787118 | 231.231313169856 | 33.5597016600397 | 0.300118721976878 | 3497.33228532654 | 31.2760496631731 |
| United States of America | 2030 | 50 to 54 | 5455.42016765964 | 52.2685752823617 | 42994.5551587953 | 411.932367074343 | 67.7842874467207 | 0.6494436766527 | 5900.28055640314 | 56.5307985400718 |
| United States of America | 2030 | 55 to 59 | 9718.52453796985 | 95.733815430574 | 78887.0266399226 | 777.088746209076 | 137.021451419259 | 1.3497508072291 | 10678.9172048514 | 105.194310586284 |
| United States of America | 2030 | 60 to 64 | 21990.4991777657 | 215.651947205264 | 151286.30985265 | 1483.60376185593 | 288.975716360445 | 2.83386818209089 | 19951.3427056277 | 195.654762952285 |
| United States of America | 2030 | 65 to 69 | 42903.3383462178 | 408.478111334831 | 317303.73401041 | 3021.01503016229 | 638.218415514405 | 6.07640950652072 | 39476.5127903253 | 375.851670481929 |
| United States of America | 2030 | 70 to 74 | 62177.788476936 | 637.394692121017 | 541413.036649843 | 5550.11370231894 | 1373.98719611933 | 14.0849677561876 | 67327.8159189678 | 690.188466815115 |
| United States of America | 2030 | 75 to 79 | 72277.72383 | 890.77609560468 | 751036.913928288 | 9256.04314026318 | 2512.38229894937 | 30.9634833024058 | 94356.8456519533 | 1162.88696032992 |
| United States of America | 2030 | 80 to 84 | 48886.6830554395 | 861.389107621027 | 772446.856145096 | 13610.6044941713 | 4463.06396552049 | 78.6397122127413 | 110312.361850921 | 1943.71679556525 |
| United States of America | 2030 | 85 to 89 | 18806.0593466491 | 583.828605482593 | 540334.40043294 | 16774.5232365889 | 5604.27603211241 | 173.98310832996 | 92723.4985154282 | 2878.57385940747 |
| United States of America | 2030 | 90 to 94 | 6070.58702077668 | 402.053587883706 | 275478.507427746 | 18244.8784470275 | 6222.93149221692 | 412.143327331829 | 71948.8724706793 | 4765.15734343642 |
| United States of America | 2030 | 95 plus | 1874.91509537283 | 306.709487219505 | 108603.263351227 | 17765.9517996445 | 4589.32822651432 | 750.748932850371 | 43845.3390850937 | 7172.4749034997 |
| United States of America | 2031 | 30 to 34 | 443.448955910007 | 3.82455028038437 | 818.857126697217 | 7.06227901039446 | 2.6040393100215 | 0.0224586824267905 | 191.096345280096 | 1.64812109980419 |
| United States of America | 2031 | 35 to 39 | 1328.38095032551 | 11.2443069828327 | 5337.86346303178 | 45.1832551468521 | 4.82876185359333 | 0.0408738777949883 | 726.119847672596 | 6.14636522117969 |
| United States of America | 2031 | 40 to 44 | 2232.58213058672 | 18.7978422688495 | 14161.0769412768 | 119.233101014388 | 15.3761365937486 | 0.129463631565309 | 1956.36982291435 | 16.4721964074023 |
| United States of America | 2031 | 45 to 49 | 2984.57317373051 | 26.5565112952098 | 26016.7355624603 | 231.494988265062 | 34.1198453362853 | 0.30359585954842 | 3565.74166930914 | 31.7277055728702 |
| United States of America | 2031 | 50 to 54 | 5583.19230910037 | 52.4118457496412 | 43965.7764367194 | 412.725796514046 | 69.5275694212874 | 0.652685424773262 | 6045.84809754082 | 56.7549961332301 |
| United States of America | 2031 | 55 to 59 | 9560.34346945215 | 95.9961164083013 | 77549.7673671351 | 778.682954163653 | 135.182038559304 | 1.35737285499368 | 10503.4750922759 | 105.466170841202 |
| United States of America | 2031 | 60 to 64 | 22145.1225095864 | 216.119408016511 | 152282.966022087 | 1486.16493105626 | 290.39462923478 | 2.83402881759749 | 20061.5713329037 | 195.785546838645 |
| United States of America | 2031 | 65 to 69 | 42363.3206106291 | 409.275494344298 | 313215.476827896 | 3026.0002584136 | 630.559373342081 | 6.09188551601074 | 38989.4422220589 | 376.680180156553 |
| United States of America | 2031 | 70 to 74 | 62959.4620130754 | 638.324667445066 | 548175.716276398 | 5557.76797649451 | 1393.69227850871 | 14.1301741113205 | 68207.7400776824 | 691.535181689196 |
| United States of America | 2031 | 75 to 79 | 74169.9879241474 | 892.617699300642 | 770075.127487082 | 9267.66617906347 | 2580.25599876815 | 31.0527510882507 | 96816.9013679793 | 1165.16777434134 |
| United States of America | 2031 | 80 to 84 | 51273.1438457861 | 863.516853727263 | 809275.953737536 | 13629.4241576138 | 4668.37298207147 | 78.6224219374646 | 115468.392991779 | 1944.66139468838 |
| United States of America | 2031 | 85 to 89 | 20136.9995343876 | 585.763066713858 | 577602.519793997 | 16801.8190971504 | 5968.82408321234 | 173.62649751704 | 98930.5171365281 | 2877.77943335205 |
| United States of America | 2031 | 90 to 94 | 6357.74219578117 | 403.982688390852 | 287761.005610391 | 18284.8660862155 | 6479.60400544829 | 411.726012980856 | 75033.5762415488 | 4767.77209836461 |
| United States of America | 2031 | 95 plus | 1936.06519914093 | 308.785775780739 | 111705.819477457 | 17816.1190758839 | 4702.54411508198 | 750.015409276018 | 45007.1714448022 | 7178.25740395861 |
| United States of America | 2032 | 30 to 34 | 449.384594270158 | 3.84322190946052 | 833.313248848804 | 7.12665226234732 | 2.69512975028532 | 0.0230492585576008 | 195.303341399748 | 1.67027105563641 |
| United States of America | 2032 | 35 to 39 | 1321.52472537358 | 11.2885357721033 | 5322.73520626018 | 45.4670923877841 | 4.89653491892662 | 0.0418264664522493 | 726.965554714623 | 6.20977913762687 |
| United States of America | 2032 | 40 to 44 | 2268.69670301624 | 18.864550658225 | 14413.4192152087 | 119.849725431364 | 15.8572807918957 | 0.131855649282121 | 1997.75598653948 | 16.6116382858677 |
| United States of America | 2032 | 45 to 49 | 3010.29447838134 | 26.6075013649934 | 26220.4938021104 | 231.758663360267 | 34.7413360995411 | 0.307072997119963 | 3640.6783508149 | 32.1793614825672 |
| United States of America | 2032 | 50 to 54 | 5695.27233769994 | 52.5551162169207 | 44812.0902056578 | 413.51922595375 | 71.0812600605605 | 0.655927172893824 | 6174.69904385569 | 56.9791937263883 |
| United States of America | 2032 | 55 to 59 | 9525.82250484956 | 96.2584173860286 | 77216.9536209809 | 780.277162118231 | 135.081165022217 | 1.36499490275826 | 10463.9338936409 | 105.738031096119 |
| United States of America | 2032 | 60 to 64 | 22228.7175311062 | 216.586868827758 | 152790.758474403 | 1488.72610025659 | 290.878191848246 | 2.8341894531041 | 20107.2613450091 | 195.916330725005 |
| United States of America | 2032 | 65 to 69 | 41699.9828398025 | 410.072877353765 | 308218.489350566 | 3030.98548666492 | 621.052707638952 | 6.10736152550076 | 38388.5566619805 | 377.508689831177 |
| United States of America | 2032 | 70 to 74 | 63479.5330232087 | 639.254642769116 | 552659.897813385 | 5565.42225067007 | 1407.64958473956 | 14.1753804664535 | 68804.8491029232 | 692.881896563278 |
| United States of America | 2032 | 75 to 79 | 75267.1224312327 | 894.459302996603 | 780835.29937686 | 9279.28921786377 | 2620.54420978079 | 31.1420188740955 | 98238.6739534533 | 1167.44858835275 |
| United States of America | 2032 | 80 to 84 | 53235.9136744284 | 865.644599833499 | 839347.614488743 | 13648.2438210564 | 4834.1039779376 | 78.6051316621879 | 119652.005858896 | 1945.60599381151 |
| United States of America | 2032 | 85 to 89 | 22002.4022374432 | 587.697527945123 | 630053.622812456 | 16829.1149577119 | 6486.93173209373 | 173.26988670412 | 107709.456568523 | 2876.98500729663 |
| United States of America | 2032 | 90 to 94 | 6704.05534191089 | 405.911788897998 | 302654.017122914 | 18324.8537254035 | 6793.19091891908 | 411.308698629882 | 78787.9000844581 | 4770.3868532928 |
| United States of America | 2032 | 95 plus | 2000.45333921218 | 310.862064341973 | 114972.768607457 | 17866.2863521233 | 4821.76380522963 | 749.281885701664 | 46230.5899121096 | 7184.03990441753 |
| United States of America | 2033 | 30 to 34 | 455.365135673352 | 3.86189353853667 | 847.91107685237 | 7.19102551430019 | 2.78742964371382 | 0.0236398346884112 | 199.557423272695 | 1.69242101146864 |
| United States of America | 2033 | 35 to 39 | 1320.40612442123 | 11.332764561374 | 5330.54466565198 | 45.7509296287162 | 4.98428481926414 | 0.0427790551095103 | 730.903962878401 | 6.27319305407406 |
| United States of America | 2033 | 40 to 44 | 2293.156282 | 18.9312590476005 | 14592.1708742475 | 120.466349848341 | 16.2615111919944 | 0.134247666998934 | 2029.06973104018 | 16.7510801643331 |
| United States of America | 2033 | 45 to 49 | 3043.93213191177 | 26.658491434777 | 26492.8813797985 | 232.022338455473 | 35.4593783324075 | 0.310550134691506 | 3725.88983815152 | 32.6310173922642 |
| United States of America | 2033 | 50 to 54 | 5781.48572378533 | 52.6983866842003 | 45453.8146811807 | 414.312655393454 | 72.3167433804996 | 0.659168921014386 | 6275.72514217439 | 57.2033913195465 |
| United States of America | 2033 | 55 to 59 | 9618.58879917521 | 96.5207183637559 | 77915.9058290026 | 781.871370072808 | 136.785534232147 | 1.37261695052283 | 10564.214303794 | 106.009891351037 |
| United States of America | 2033 | 60 to 64 | 22138.6039484745 | 217.054329639005 | 152104.859123603 | 1491.28726945693 | 289.091464645909 | 2.8343500886107 | 19995.9587668242 | 196.047114611366 |
| United States of America | 2033 | 65 to 69 | 41085.4277510104 | 410.870260363232 | 303585.261565543 | 3035.97071491624 | 612.259935661067 | 6.12283753499078 | 37832.248218874 | 378.337199505801 |
| United States of America | 2033 | 70 to 74 | 63736.2556259687 | 640.184618093165 | 554850.928890881 | 5573.07652484564 | 1415.78996307595 | 14.2205868215864 | 69116.8312872421 | 694.228611437359 |
| United States of America | 2033 | 75 to 79 | 77188.0358549493 | 896.300906692565 | 800118.869720789 | 9290.91225666406 | 2689.58968634687 | 31.2312866599404 | 100735.271353734 | 1169.72940236417 |
| United States of America | 2033 | 80 to 84 | 54572.9532439718 | 867.772345939734 | 859501.941968722 | 13667.063484499 | 4942.27617833125 | 78.5878413869113 | 122415.763756805 | 1946.55059293463 |
| United States of America | 2033 | 85 to 89 | 23550.279012178 | 589.631989176388 | 673255.836184765 | 16856.4108182734 | 6906.26690359112 | 172.9132759 | 114876.892576746 | 2876.19058124121 |
| United States of America | 2033 | 90 to 94 | 7170.08346186719 | 407.840889405144 | 322864.746445924 | 18364.8413645915 | 7223.71296153994 | 410.891384278909 | 83912.184343474 | 4773.001608 |
| United States of America | 2033 | 95 plus | 2074.04274523541 | 312.938352903207 | 118743.804725482 | 17916.4536283628 | 4961.10906676945 | 748.54836212731 | 47651.5545634553 | 7189.82240487644 |
| United States of America | 2034 | 30 to 34 | 461.018786625783 | 3.88056516761282 | 861.955666566432 | 7.25539876625305 | 2.87862053923284 | 0.0242304108192216 | 203.694408620148 | 1.71457096730086 |
| United States of America | 2034 | 35 to 39 | 1325.86206285157 | 11.3769933506446 | 5364.84017204991 | 46.0347668696482 | 5.09643678513851 | 0.0437316437667713 | 738.461079344738 | 6.33660697052124 |
| United States of America | 2034 | 40 to 44 | 2301.6974149691 | 18.9979674369759 | 14669.7992713064 | 121.082974265318 | 16.5545714369584 | 0.136639684715747 | 2046.37001575871 | 16.8905220427985 |
| United States of America | 2034 | 45 to 49 | 3087.5679848605 | 26.7094815045606 | 26851.8450516341 | 232.286013550679 | 36.3009874245184 | 0.314027272263049 | 3824.29748489478 | 33.0826733019613 |
| United States of America | 2034 | 50 to 54 | 5843.11102566547 | 52.8416571514798 | 45901.4927210988 | 415.106084833157 | 73.2478747448303 | 0.662410669134948 | 6350.21299561595 | 57.4275889127047 |
| United States of America | 2034 | 55 to 59 | 9773.96710961641 | 96.7830193414832 | 79120.9743533397 | 783.465578027385 | 139.388197066598 | 1.38023899828741 | 10733.2293579703 | 106.281751605954 |
| United States of America | 2034 | 60 to 64 | 21927.1187697769 | 217.521790450252 | 150586.256534032 | 1493.84843865726 | 285.730699316495 | 2.83451072411731 | 19775.5639628604 | 196.177898497726 |
| United States of America | 2034 | 65 to 69 | 40597.6091202485 | 411.667643372699 | 299891.29026797 | 3040.95594316755 | 605.344767674023 | 6.1383135444808 | 37392.351575158 | 379.165709180425 |
| United States of America | 2034 | 70 to 74 | 63907.0976539238 | 641.114593417215 | 556294.166152624 | 5580.7307990212 | 1422.03195343892 | 14.2657931767193 | 69335.811040129 | 695.575326311441 |
| United States of America | 2034 | 75 to 79 | 78689.7680909294 | 898.142510388527 | 815031.397123285 | 9302.53529546436 | 2744.11592515758 | 31.3205544457852 | 102684.385896508 | 1172.01021637558 |
| United States of America | 2034 | 80 to 84 | 57140.7184931804 | 869.90009204597 | 898978.173973755 | 13685.8831479415 | 5161.02686271092 | 78.5705511116346 | 127924.201357958 | 1947.49519205776 |
| United States of America | 2034 | 85 to 89 | 24600.2494305507 | 591.566450407652 | 702107.760379884 | 16883.7066788349 | 7175.75683831375 | 172.556665078281 | 119573.14783567 | 2875.39615518579 |
| United States of America | 2034 | 90 to 94 | 7616.98487128522 | 409.76998991229 | 342117.059647015 | 18404.8290037795 | 7630.07262041124 | 410.474069927936 | 88771.2582294169 | 4775.61636314919 |
| United States of America | 2034 | 95 plus | 2165.5461017224 | 315.014641464441 | 123510.277745224 | 17966.6209046022 | 5140.8007605936 | 747.814838552956 | 49465.6822293845 | 7195.60490533535 |
| United States of America | 2035 | 30 to 34 | 464.505009389226 | 3.89003805403741 | 870.307852771681 | 7.28846965603458 | 2.93140662449701 | 0.0245493225921176 | 206.111106058273 | 1.72609558502002 |
| United States of America | 2035 | 35 to 39 | 1335.83553812675 | 11.3993692209069 | 5411.53115628073 | 46.1793685975679 | 5.18445980806603 | 0.0442416524162643 | 746.386737048788 | 6.36930052717299 |
| United States of America | 2035 | 40 to 44 | 2291.52624135086 | 19.0316773625782 | 14616.8045590501 | 121.396082409999 | 16.6042904336466 | 0.137902631296694 | 2042.3274374929 | 16.9620212754766 |
| United States of America | 2035 | 45 to 49 | 3137.09250361975 | 26.7537427153826 | 27299.2016674257 | 232.812968346622 | 37.1095984453227 | 0.316477964207863 | 3893.55082980713 | 33.204968304164 |
| United States of America | 2035 | 50 to 54 | 5874.77866231885 | 52.8538669329599 | 46093.6567167511 | 414.692729478779 | 73.8602201849559 | 0.664501332507035 | 6443.55473075375 | 57.9709983797205 |
| United States of America | 2035 | 55 to 59 | 9964.7940555882 | 96.8934723039206 | 80567.2487525426 | 783.402089602621 | 141.742688100779 | 1.37824636888247 | 10936.5849892022 | 106.34275920198 |
| United States of America | 2035 | 60 to 64 | 21565.3309920147 | 217.790378004314 | 147954.418572466 | 1494.20608291325 | 280.637842314699 | 2.83418890174537 | 19419.5441786457 | 196.119867991119 |
| United States of America | 2035 | 65 to 69 | 40505.0846953759 | 412.057011107926 | 298967.612964837 | 3041.38855511208 | 600.886092184495 | 6.1127961840833 | 37209.9187566693 | 378.535386896557 |
| United States of America | 2035 | 70 to 74 | 63662.0755977474 | 641.695799718286 | 553803.916725466 | 5582.18757232668 | 1413.26219332283 | 14.245285043581 | 69003.8485155088 | 695.539366900027 |
| United States of America | 2035 | 75 to 79 | 79838.5533787334 | 898.630052592122 | 826615.345571506 | 9304.04372359585 | 2781.73196928617 | 31.3100355663843 | 104122.071352471 | 1171.95538365548 |
| United States of America | 2035 | 80 to 84 | 60298.8891097788 | 870.989479752282 | 947629.950998704 | 13688.0750243239 | 5438.58500934528 | 78.5578374297115 | 134844.721510866 | 1947.76944600532 |
| United States of America | 2035 | 85 to 89 | 25368.9575473425 | 592.614960751778 | 723029.36746308 | 16889.8552264873 | 7369.51240090683 | 172.15068037672 | 122962.011007072 | 2872.37373435349 |
| United States of America | 2035 | 90 to 94 | 7962.1400994645 | 410.835086797292 | 356925.555422583 | 18416.8502074047 | 7927.02138880353 | 409.023011354965 | 92405.0065318812 | 4767.96670302033 |
| United States of America | 2035 | 95 plus | 2266.4141248868 | 316.28516911561 | 128904.80342901 | 17989.0678868741 | 5345.18869669413 | 745.937775592177 | 51523.372173831 | 7190.24749381165 |
| United States of America | 2036 | 30 to 34 | 468.466849944887 | 3.89951094 | 879.571589517293 | 7.32154054581611 | 2.987539616 | 0.0248682343650136 | 208.748603403423 | 1.73762020273917 |
| United States of America | 2036 | 35 to 39 | 1349.96437825312 | 11.4217450911693 | 5475.14493621577 | 46.3239703254875 | 5.28930980548131 | 0.0447516610657574 | 756.667557712574 | 6.40199408382474 |
| United States of America | 2036 | 40 to 44 | 2273.36879143664 | 19.0653872881805 | 14512.68056 | 121.709190554679 | 16.5941911804439 | 0.13916557787764 | 2031.08771651027 | 17.0335205081546 |
| United States of America | 2036 | 45 to 49 | 3187.55101635156 | 26.7980039262046 | 27755.1608402134 | 233.339923142566 | 37.935712109849 | 0.318928656152677 | 3964.1889864993 | 33.3272633063668 |
| United States of America | 2036 | 50 to 54 | 5906.50735708601 | 52.8660767144399 | 46285.7152115166 | 414.2793741 | 74.4755573427915 | 0.666591995879123 | 6537.57195392328 | 58.5144078467363 |
| United States of America | 2036 | 55 to 59 | 10183.2072537463 | 97.003925266358 | 82232.7478372701 | 783.338601177856 | 144.475360397644 | 1.37625373947754 | 11169.9769561712 | 106.403766798005 |
| United States of America | 2036 | 60 to 64 | 21191.8381476459 | 218.058965558376 | 145247.65136994 | 1494.56372716923 | 275.406481564486 | 2.83386707937343 | 19054.0696928582 | 196.061837484513 |
| United States of America | 2036 | 65 to 69 | 40763.2675911036 | 412.446378843152 | 300631.976803378 | 3041.82116705662 | 601.623358380668 | 6.08727882368579 | 37349.4496813087 | 377.905064612688 |
| United States of America | 2036 | 70 to 74 | 62844.3451180611 | 642.277006019358 | 546338.213862263 | 5583.64434563215 | 1391.83993979125 | 14.2247769104427 | 68052.3446447102 | 695.503407488614 |
| United States of America | 2036 | 75 to 79 | 80873.2449902484 | 899.117594795716 | 837009.756334673 | 9305.55215172735 | 2815.30858227494 | 31.2995166869834 | 105409.349020178 | 1171.90055093538 |
| United States of America | 2036 | 80 to 84 | 61945.7938993451 | 872.078867458594 | 972451.556279051 | 13690.2669007064 | 5579.24314994375 | 78.5451237477884 | 138374.082949474 | 1948.04369995288 |
| United States of America | 2036 | 85 to 89 | 26683.8016631426 | 593.663471095903 | 759436.339878855 | 16896.0037741396 | 7719.52734035142 | 171.74469567516 | 128970.713341244 | 2869.3513135212 |
| United States of America | 2036 | 90 to 94 | 8569.61215452298 | 411.900183682294 | 383413.959727478 | 18428.87141 | 8479.56300766607 | 407.571952781994 | 99038.7292995501 | 4760.31704289147 |
| United States of America | 2036 | 95 plus | 2376.62494128618 | 317.555696766779 | 134800.338662472 | 18011.5148691459 | 5568.63966061889 | 744.060712631397 | 53772.5795626529 | 7184.89008228796 |
| United States of America | 2037 | 30 to 34 | 473.880324037398 | 3.9089838268866 | 891.588659512577 | 7.35461143559764 | 3.05340044932249 | 0.0251871461379095 | 212.046237849828 | 1.74914482045832 |
| United States of America | 2037 | 35 to 39 | 1363.78788408913 | 11.4441209614316 | 5537.62720360409 | 46.4685720534071 | 5.39380149679759 | 0.0452616697152504 | 766.817221834235 | 6.43468764047649 |
| United States of America | 2037 | 40 to 44 | 2257.1584294429 | 19.0990972137828 | 14420.7685319546 | 122.02229869936 | 16.5960424290206 | 0.140428524458587 | 2021.49552209963 | 17.1050197408326 |
| United States of America | 2037 | 45 to 49 | 3232.84241812843 | 26.8422651370266 | 28166.5783172659 | 233.866877938509 | 38.7064498296331 | 0.321379348097491 | 4028.61496284223 | 33.4495583085695 |
| United States of America | 2037 | 50 to 54 | 5947.93421028096 | 52.8782865 | 46553.0941836576 | 413.866018770022 | 75.215759215057 | 0.668682659251211 | 6643.02940323326 | 59.0578173137521 |
| United States of America | 2037 | 55 to 59 | 10372.272032413 | 97.1143782287955 | 83657.4634350601 | 783.275112753092 | 146.777545582972 | 1.37426111007261 | 11370.9382897221 | 106.46477439403 |
| United States of America | 2037 | 60 to 64 | 21091.7690621844 | 218.327553112438 | 144418.493601614 | 1494.92137142522 | 273.737699781507 | 2.83354525700149 | 18935.1594572122 | 196.003806977907 |
| United States of America | 2037 | 65 to 69 | 40885.3698101183 | 412.835746578379 | 301290.941595041 | 3042.25377900115 | 600.3292137 | 6.06176146328829 | 37363.5701074213 | 377.27474232882 |
| United States of America | 2037 | 70 to 74 | 61839.4757337145 | 642.858212320429 | 537256.456393735 | 5585.10111893763 | 1366.37366923991 | 14.2042687773044 | 66900.1990714581 | 695.4674481 |
| United States of America | 2037 | 75 to 79 | 81556.9062889602 | 899.60513699931 | 843764.710002768 | 9307.06057985885 | 2836.61548507859 | 31.2889978075825 | 106237.845355563 | 1171.84571821528 |
| United States of America | 2037 | 80 to 84 | 62947.1448306482 | 873.168255164907 | 987096.336394306 | 13692.4587770888 | 5661.44149317737 | 78.5324100658654 | 140455.23493884 | 1948.31795390045 |
| United States of America | 2037 | 85 to 89 | 27832.5028900339 | 594.711981440028 | 791020.22159529 | 16902.1523217919 | 8018.64653340311 | 171.338710973599 | 134144.106187974 | 2866.3288926889 |
| United States of America | 2037 | 90 to 94 | 9426.18443776966 | 412.965280567295 | 420924.622874001 | 18440.8926146552 | 9269.95714406642 | 406.120894209023 | 108482.53213866 | 4752.66738276262 |
| United States of America | 2037 | 95 plus | 2508.1995309715 | 318.826224417947 | 141872.817205867 | 18033.9618514178 | 5838.74392828576 | 742.183649670618 | 56481.239930249 | 7179.53267076426 |
| United States of America | 2038 | 30 to 34 | 478.273856335042 | 3.91845671331119 | 901.716051407284 | 7.38768232537917 | 3.11318500354124 | 0.0255060579108055 | 214.901483808101 | 1.76066943817747 |
| United States of America | 2038 | 35 to 39 | 1377.65292568592 | 11.4664968316939 | 5600.38311420913 | 46.6131737813268 | 5.49928086479299 | 0.0457716783647435 | 777.029528593485 | 6.46738119712824 |
| United States of America | 2038 | 40 to 44 | 2250.63476996579 | 19.1328071393851 | 14390.5867150218 | 122.335406844041 | 16.6674837103608 | 0.141691471039534 | 2020.51223049137 | 17.1765189735107 |
| United States of America | 2038 | 45 to 49 | 3261.51954983426 | 26.8865263478486 | 28433.5751645049 | 234.393832734452 | 39.2827988546054 | 0.323830040042305 | 4072.49543807332 | 33.5718533107722 |
| United States of America | 2038 | 50 to 54 | 6004.69718134048 | 52.8904962774001 | 46939.586832549 | 413.452663415644 | 76.1533916896818 | 0.670773322623298 | 6766.57138132839 | 59.6012267807679 |
| United States of America | 2038 | 55 to 59 | 10513.8990785098 | 97.2248311912329 | 84696.5520475627 | 783.211624328327 | 148.397195835512 | 1.37226848066768 | 11519.7044559515 | 106.525781990056 |
| United States of America | 2038 | 60 to 64 | 21272.1216368789 | 218.5961407 | 145509.234543946 | 1495.2790156812 | 275.707857156748 | 2.83322343462955 | 19067.959656659 | 195.9457765 |
| United States of America | 2038 | 65 to 69 | 40686.3513678748 | 413.225114313606 | 299584.423395707 | 3042.68639094568 | 594.331612492678 | 6.03624410289079 | 37084.5979198359 | 376.644420044952 |
| United States of America | 2038 | 70 to 74 | 60910.4783460497 | 643.4394186 | 528845.301789933 | 5586.55789224311 | 1342.68995740567 | 14.1837606441662 | 65832.2499455943 | 695.431488665787 |
| United States of America | 2038 | 75 to 79 | 81901.2420669574 | 900.092679202904 | 847005.404260747 | 9308.56900799035 | 2846.09166741789 | 31.2784789281817 | 106623.607970887 | 1171.79088549518 |
| United States of America | 2038 | 80 to 84 | 64601.3276680015 | 874.257642871219 | 1011935.8079138 | 13694.6506534712 | 5802.03865056544 | 78.5196963839423 | 143986.895324227 | 1948.59220784801 |
| United States of America | 2038 | 85 to 89 | 28618.8088784518 | 595.760491784153 | 812231.488517873 | 16908.3008694442 | 8211.17058232947 | 170.932726272039 | 137545.912843423 | 2863.3064718566 |
| United States of America | 2038 | 90 to 94 | 10133.2485775129 | 414.030377452297 | 451628.607182579 | 18452.9138182805 | 9904.1525927499 | 404.669835636052 | 116132.648005258 | 4745.01772263376 |
| United States of America | 2038 | 95 plus | 2685.03877667368 | 320.096752069116 | 151460.949142844 | 18056.4088336896 | 6209.84711370533 | 740.306586709838 | 60178.4886513143 | 7174.17525924056 |
| United States of America | 2039 | 30 to 34 | 480.033965393437 | 3.92792959973578 | 906.893441348671 | 7.42075321516069 | 3.15608064978213 | 0.0258249696837015 | 216.58059761457 | 1.77219405589662 |
| United States of America | 2039 | 35 to 39 | 1390.49091023924 | 11.4888727019563 | 5659.06364490777 | 46.7577755092464 | 5.60144295905336 | 0.0462816870142365 | 786.699887402149 | 6.50007475377998 |
| United States of America | 2039 | 40 to 44 | 2255.16574760611 | 19.1665170649874 | 14431.0376819891 | 122.648514988722 | 16.8202655179125 | 0.142954417620481 | 2029.43183369184 | 17.2480182061887 |
| United States of America | 2039 | 45 to 49 | 3267.72386117585 | 26.9307875586706 | 28504.783279245 | 234.920787530395 | 39.5902025157432 | 0.326280731987119 | 4088.37551387751 | 33.694148312975 |
| United States of America | 2039 | 50 to 54 | 6080.86821923317 | 52.9027060588802 | 47476.5430503381 | 413.039308061265 | 77.3419269659285 | 0.672863985995386 | 6913.28732238066 | 60.1446362477837 |
| United States of America | 2039 | 55 to 59 | 10610.8084113855 | 97.3352841536703 | 85373.304244811 | 783.148135903562 | 149.37784026543 | 1.37027585126275 | 11619.3424955438 | 106.586789586081 |
| United States of America | 2039 | 60 to 64 | 21590.079641158 | 218.864728220562 | 147538.229959732 | 1495.63665993719 | 279.453760875394 | 2.83290161225762 | 19323.4975342516 | 195.887745964694 |
| United States of America | 2039 | 65 to 69 | 40266.9994685131 | 413.614482048832 | 296259.624820233 | 3043.11900289021 | 585.167930645086 | 6.01072674249328 | 36606.4539125866 | 376.014097761083 |
| United States of America | 2039 | 70 to 74 | 60180.3140897022 | 644.020624922572 | 522170.354017788 | 5588.01466554858 | 1323.48088191725 | 14.1632525110279 | 64981.0266125161 | 695.395529254373 |
| United States of America | 2039 | 75 to 79 | 82144.8849034151 | 900.580221406498 | 849202.793103438 | 9310.07743612184 | 2852.05350763785 | 31.2679600487808 | 106877.900385229 | 1171.73605277509 |
| United States of America | 2039 | 80 to 84 | 65927.7595035616 | 875.347030577531 | 1031593.30953641 | 13696.8425298536 | 5912.84290016286 | 78.5069827020192 | 146781.0993 | 1948.86646179557 |
| United States of America | 2039 | 85 to 89 | 30027.1856750898 | 596.809002128279 | 851014.832932939 | 16914.4494170965 | 8579.69319069388 | 170.526741570478 | 143909.15682794 | 2860.2840510243 |
| United States of America | 2039 | 90 to 94 | 10584.6855383165 | 415.095474337298 | 470844.764097584 | 18464.9350219058 | 10281.8368838423 | 403.218777063081 | 120800.043173037 | 4737.3680625049 |
| United States of America | 2039 | 95 plus | 2856.63053576082 | 321.367279720285 | 160702.768559524 | 18078.8558159615 | 6563.89265231015 | 738.429523749059 | 63723.5501601768 | 7168.81784771687 |
| United States of America | 2040 | 30 to 34 | 479.190316502582 | 3.93749475861841 | 907.19814928679 | 7.45442433794634 | 3.18370871993609 | 0.0261604543455931 | 217.11475425481 | 1.78402646601151 |
| United States of America | 2040 | 35 to 39 | 1400.15571368175 | 11.5114242593202 | 5705.04274062345 | 46.9041884150026 | 5.69422065828397 | 0.0468152143245056 | 794.674277898101 | 6.53343958208723 |
| United States of America | 2040 | 40 to 44 | 2271.55642267533 | 19.2004658262624 | 14547.6471848127 | 122.964853453012 | 17.0674494053042 | 0.144263631656595 | 2049.16901134574 | 17.3207229993615 |
| United States of America | 2040 | 45 to 49 | 3253.28959765797 | 26.9753416716836 | 28396.1264089596 | 235.452513230128 | 39.6549373378665 | 0.328807335328594 | 4078.55981910787 | 33.818244993633 |
| United States of America | 2040 | 50 to 54 | 6177.37627655621 | 52.9791406508894 | 48257.7765266619 | 413.874016353511 | 78.9806289802866 | 0.677362955422073 | 7035.85821693456 | 60.3417543932577 |
| United States of America | 2040 | 55 to 59 | 10669.9902745853 | 97.3551872167073 | 85756.6262767165 | 782.461107404097 | 150.589797956136 | 1.37401230888334 | 11779.2282725106 | 107.476102997964 |
| United States of America | 2040 | 60 to 64 | 22014.0199871601 | 219.076822881019 | 150278.931416771 | 1495.53016032252 | 284.309166225456 | 2.82935824029201 | 19693.8535188592 | 195.987232759501 |
| United States of America | 2040 | 65 to 69 | 39618.0644992228 | 414.063466417967 | 291229.554210843 | 3043.75088142598 | 575.057002589375 | 6.01013953836017 | 35968.113003128 | 375.916434556779 |
| United States of America | 2040 | 70 to 74 | 60097.9777656905 | 644.577518478498 | 521071.045248022 | 5588.71851905661 | 1315.8780616757 | 14.1133769745484 | 64740.5505217883 | 694.371174400641 |
| United States of America | 2040 | 75 to 79 | 81923.4447966248 | 901.3429114 | 846396.328971468 | 9312.27114858482 | 2838.46476705016 | 31.2295229217219 | 106494.633203489 | 1171.68147629502 |
| United States of America | 2040 | 80 to 84 | 67023.7513436181 | 875.824650739948 | 1048328.03947137 | 13698.8980864966 | 6006.18152235534 | 78.485040431842 | 149133.529011764 | 1948.78410029165 |
| United States of America | 2040 | 85 to 89 | 31732.8451418587 | 597.615048790973 | 898280.592710686 | 16917.0459768404 | 9053.56843580871 | 170.503108634315 | 151898.428388046 | 2860.65703489822 |
| United States of America | 2040 | 90 to 94 | 10892.1100269772 | 415.88542674512 | 483772.772578853 | 18471.5399930147 | 10539.3858896369 | 402.417620413953 | 123954.428779797 | 4732.8607939466 |
| United States of America | 2040 | 95 plus | 2995.00410946471 | 322.244422270309 | 168138.643816298 | 18090.7064423294 | 6841.52139900811 | 736.106539455587 | 66530.0036222917 | 7158.22810164314 |
| United States of America | 2041 | 30 to 34 | 475.576828752593 | 3.94705991750104 | 902.232235396682 | 7.48809546073199 | 3.19246601556013 | 0.0264959390074846 | 216.381024622528 | 1.7958588761264 |
| United States of America | 2041 | 35 to 39 | 1411.21965727222 | 11.5339758166841 | 5756.79492706116 | 47.0506013207588 | 5.79327336939983 | 0.0473487416347747 | 803.469993062187 | 6.56680441039448 |
| United States of America | 2041 | 40 to 44 | 2294.90070483948 | 19.2344145875375 | 14708.9526918992 | 123.281191917301 | 17.3686193913146 | 0.14557284569271 | 2075.24848333696 | 17.3934277925343 |
| United States of America | 2041 | 45 to 49 | 3227.64841121199 | 27.0198957846966 | 28189.3816290895 | 235.984238929862 | 39.5793332902032 | 0.33133393867007 | 4054.56579295501 | 33.942341674291 |
| United States of America | 2041 | 50 to 54 | 6275.68455371954 | 53.0555752428986 | 49053.8671126829 | 414.708724645756 | 80.6541127855474 | 0.681861924848761 | 7160.84719751987 | 60.5388725387318 |
| United States of America | 2041 | 55 to 59 | 10729.400910091 | 97.3750902797443 | 86140.7931904094 | 781.774078904632 | 151.809038910633 | 1.37774876650394 | 11940.3842821602 | 108.365416409846 |
| United States of America | 2041 | 60 to 64 | 22498.2929716574 | 219.288917541475 | 153425.353239705 | 1495.42366070785 | 289.918941203439 | 2.8258148683264 | 20117.8268096911 | 196.086719554308 |
| United States of America | 2041 | 65 to 69 | 38949.1981842652 | 414.512450787102 | 286062.016331115 | 3044.38275996176 | 564.680854387031 | 6.00955233422705 | 35313.3899331113 | 375.818771352474 |
| United States of America | 2041 | 70 to 74 | 60528.8910830501 | 645.134412034424 | 524420.231962569 | 5589.42237256464 | 1319.48959923993 | 14.0635014380689 | 65052.3570596552 | 693.346819546909 |
| United States of America | 2041 | 75 to 79 | 80958.9349587577 | 902.105601438102 | 835921.153420466 | 9314.4648610478 | 2799.22559190141 | 31.1910857946629 | 105146.964864022 | 1171.62689981495 |
| United States of America | 2041 | 80 to 84 | 68024.2782322024 | 876.302270902364 | 1063557.07797917 | 13700.9536431395 | 6090.81568944648 | 78.4630981616649 | 151270.895526005 | 1948.70173878772 |
| United States of America | 2041 | 85 to 89 | 32696.3822081236 | 598.421095453667 | 924451.199003261 | 16919.6425365843 | 9314.61497332673 | 170.479475698152 | 156320.242908168 | 2861.03001877214 |
| United States of America | 2041 | 90 to 94 | 11521.2825712684 | 416.675379152941 | 510929.947330499 | 18478.1449641236 | 11104.8960313293 | 401.616463764825 | 130741.339153749 | 4728.35352538831 |
| United States of America | 2041 | 95 plus | 3235.40653489905 | 323.121564820333 | 181260.360852155 | 18102.5570686974 | 7347.35272433159 | 733.783555162114 | 71569.0884251658 | 7147.63835556941 |
| United States of America | 2042 | 30 to 34 | 470.137349258623 | 3.95662507638367 | 893.757516835332 | 7.52176658351764 | 3.18818542501003 | 0.0268314236693762 | 214.794976320617 | 1.80769128624129 |
| United States of America | 2042 | 35 to 39 | 1426.58223168287 | 11.556527374048 | 5826.18114462578 | 47.197014226515 | 5.91077162531164 | 0.0478822689450437 | 814.750301476911 | 6.60016923870173 |
| United States of America | 2042 | 40 to 44 | 2317.7594417455 | 19.2683633488125 | 14867.3417577011 | 123.59753038159 | 17.6681991405611 | 0.146882059728824 | 2100.97209495461 | 17.466132585707 |
| United States of America | 2042 | 45 to 49 | 3204.79055442795 | 27.0644498977095 | 28006.633509314 | 236.515964629595 | 39.5335251807703 | 0.333860542011545 | 4033.9190441917 | 34.066438354949 |
| United States of America | 2042 | 50 to 54 | 6363.81262448242 | 53.1320098349078 | 49771.1370747773 | 415.543432938001 | 82.2079220701988 | 0.686360894275449 | 7274.56886117371 | 60.7359906842059 |
| United States of America | 2042 | 55 to 59 | 10806.3017585592 | 97.3949933427813 | 86664.2326949425 | 781.087050405166 | 153.28042740697 | 1.38148522412454 | 12122.179369613 | 109.254729821729 |
| United States of America | 2042 | 60 to 64 | 22917.4246208862 | 219.501012201932 | 156121.459212882 | 1495.31716109317 | 294.664674338822 | 2.82227149636079 | 20483.1975513955 | 196.186206349115 |
| United States of America | 2042 | 65 to 69 | 38780.5113955308 | 414.961435156237 | 284573.974551034 | 3045.01463849753 | 561.57204250852 | 6.00896513009394 | 35113.2791667629 | 375.721108148169 |
| United States of America | 2042 | 70 to 74 | 60743.499970195 | 645.69130559035 | 525891.907335458 | 5590.12622607267 | 1318.33381859965 | 14.0136259015894 | 65130.3327911557 | 692.322464693177 |
| United States of America | 2042 | 75 to 79 | 79741.0114973068 | 902.868291453904 | 822844.024381971 | 9316.65857351077 | 2751.39102689575 | 31.152648667604 | 103472.857546624 | 1171.57232333488 |
| United States of America | 2042 | 80 to 84 | 68708.7947886992 | 876.779891064781 | 1073835.35673031 | 13703.0091997825 | 6147.03569055552 | 78.4411558914877 | 152703.421097488 | 1948.61937728379 |
| United States of America | 2042 | 85 to 89 | 33335.8807875607 | 599.227142116362 | 941408.867397823 | 16922.2390963283 | 9482.70739837993 | 170.45584276199 | 159184.026685584 | 2861.40300264606 |
| United States of America | 2042 | 90 to 94 | 12123.0596396184 | 417.465331560762 | 536791.222999172 | 18484.7499352325 | 11639.5482577415 | 400.815307115696 | 137178.983667541 | 4723.84625683001 |
| United States of America | 2042 | 95 plus | 3571.65483047739 | 323.998707370357 | 199687.252675861 | 18114.4076950653 | 8063.37995126752 | 731.460570868642 | 78676.4686450392 | 7137.04860949568 |
| United States of America | 2043 | 30 to 34 | 464.900954616975 | 3.9661902352663 | 885.618186182047 | 7.55543770630329 | 3.18439632695788 | 0.0271669083312678 | 213.277289592085 | 1.81952369635617 |
| United States of America | 2043 | 35 to 39 | 1438.96386548283 | 11.5790789314119 | 5883.49741071767 | 47.3434271322711 | 6.01676365992946 | 0.0484157962553128 | 824.367454390549 | 6.63353406700898 |
| United States of America | 2043 | 40 to 44 | 2340.71649661934 | 19.3023121100876 | 15026.5540891287 | 123.91386884588 | 17.9705807873319 | 0.148191273764939 | 2126.86675824772 | 17.5388373788798 |
| United States of America | 2043 | 45 to 49 | 3195.58987430108 | 27.1090040107225 | 27943.0110616842 | 237.047690329328 | 39.6530744954694 | 0.336387145353021 | 4030.35565281213 | 34.190535035607 |
| United States of America | 2043 | 50 to 54 | 6419.38226173873 | 53.2084444269171 | 50234.3280803944 | 416.378141230246 | 83.3494307560175 | 0.690859863702136 | 7351.33158254796 | 60.93310883 |
| United States of America | 2043 | 55 to 59 | 10910.9613168275 | 97.4148964058183 | 87408.7512775493 | 780.400021905701 | 155.151837577165 | 1.38522168174513 | 12336.6901710233 | 110.144043233612 |
| United States of America | 2043 | 60 to 64 | 23231.9849450561 | 219.713106862389 | 158100.315785495 | 1495.2106614785 | 298.046167046236 | 2.81872812439519 | 20754.8212902273 | 196.285693143922 |
| United States of America | 2043 | 65 to 69 | 39125.1645003287 | 415.410419525372 | 286852.267993011 | 3045.6465170333 | 565.895229594748 | 6.00837792596083 | 35377.8537630331 | 375.623444943865 |
| United States of America | 2043 | 70 to 74 | 60475.0534284857 | 646.248199146276 | 523182.498951457 | 5590.8300795807 | 1306.70932701651 | 13.9637503651099 | 64690.7646052703 | 691.298109839445 |
| United States of America | 2043 | 75 to 79 | 78625.0039881577 | 903.630981469707 | 810834.082910759 | 9318.85228597375 | 2707.25003527975 | 31.1142115405451 | 101933.852875257 | 1171.51774685482 |
| United States of America | 2043 | 80 to 84 | 69107.2675606142 | 877.257511227198 | 1079636.8967339 | 13705.0647564254 | 6177.59039764703 | 78.4192136213105 | 153498.906738213 | 1948.53701577987 |
| United States of America | 2043 | 85 to 89 | 34292.1427523305 | 600.033188779056 | 967261.296927145 | 16924.8356560722 | 9740.27066875205 | 170.432209825827 | 163551.670957771 | 2861.77598651998 |
| United States of America | 2043 | 90 to 94 | 12533.165973468 | 418.255283968584 | 554099.921742737 | 18491.3549063414 | 11986.5640236834 | 400.014150466568 | 141416.644552208 | 4719.33898827171 |
| United States of America | 2043 | 95 plus | 3859.42438554065 | 324.875849920381 | 215334.329718548 | 18126.2583214333 | 8661.92849586118 | 729.13758657517 | 84660.1220952051 | 7126.45886342195 |
| United States of America | 2044 | 30 to 34 | 461.783472182197 | 3.97575539414893 | 881.474003914533 | 7.58910882908894 | 3.19439937084437 | 0.0275023929931593 | 212.71177369032 | 1.83135610647106 |
| United States of America | 2044 | 35 to 39 | 1443.59366610969 | 11.6016304887758 | 5909.17219349334 | 47.4898400380273 | 6.09077607910301 | 0.0489493235655819 | 829.563829599933 | 6.66689889531623 |
| United States of America | 2044 | 40 to 44 | 2361.95365655347 | 19.3362608713627 | 15174.9086528523 | 124.230207310169 | 18.2617118256401 | 0.149500487801053 | 2151.27664586032 | 17.6115421720526 |
| United States of America | 2044 | 45 to 49 | 3201.94078556135 | 27.1535581237355 | 28015.3053432929 | 237.579416029061 | 39.9646245176178 | 0.338913748694496 | 4046.37279332404 | 34.314631716265 |
| United States of America | 2044 | 50 to 54 | 6430.99414384864 | 53.2848790189263 | 50353.7484070206 | 417.212849522491 | 83.9234069037959 | 0.695358833128824 | 7377.85538651066 | 61.1302269751541 |
| United States of America | 2044 | 55 to 59 | 11050.8737270306 | 97.4347994688553 | 88433.5974459675 | 779.712993406235 | 157.533048704722 | 1.38895813936573 | 12593.1967886886 | 111.033356645494 |
| United States of America | 2044 | 60 to 64 | 23448.44038 | 219.925201522846 | 159408.110395213 | 1495.10416186383 | 300.155195366975 | 2.81518475242958 | 20938.6016320299 | 196.385179938729 |
| United States of America | 2044 | 65 to 69 | 39723.2354233085 | 415.859403894507 | 290983.040755827 | 3046.27839556908 | 573.869156215283 | 6.00779072182772 | 35870.5343581616 | 375.52578173956 |
| United States of America | 2044 | 70 to 74 | 59885.3032905558 | 646.805092702202 | 517699.550019811 | 5591.53393308873 | 1288.23446732352 | 13.9138748286304 | 63909.9067667088 | 690.273754985713 |
| United States of America | 2044 | 75 to 79 | 77788.0120886845 | 904.393671485509 | 801714.631212121 | 9321.04599843672 | 2672.86558050648 | 31.0757744134862 | 100758.988183632 | 1171.46317037475 |
| United States of America | 2044 | 80 to 84 | 69440.5139928147 | 877.735131389614 | 1084415.38439267 | 13707.1203130684 | 6202.26606361091 | 78.3972713511334 | 154148.658115582 | 1948.45465427594 |
| United States of America | 2044 | 85 to 89 | 35100.4933875876 | 600.839235441751 | 988885.524633306 | 16927.4322158161 | 9955.11739826076 | 170.408576889664 | 167204.195537828 | 2862.1489703939 |
| United States of America | 2044 | 90 to 94 | 13198.8312377898 | 419.045236376405 | 582637.456464404 | 18497.9598774503 | 12574.1673593355 | 399.21299381744 | 148504.893460184 | 4714.83171971341 |
| United States of America | 2044 | 95 plus | 4039.06673164928 | 325.752992470405 | 224897.496322308 | 18138.1089478012 | 9011.89781217315 | 726.814602281697 | 88230.8708837505 | 7115.86911734822 |
| United States of America | 2045 | 30 to 34 | 461.066198339241 | 3.98532055303156 | 881.887950145918 | 7.6227799518746 | 3.22059524433581 | 0.0278378776550509 | 213.240543854967 | 1.84318851658595 |
| United States of America | 2045 | 35 to 39 | 1440.53594317168 | 11.6241820461397 | 5903.36027869819 | 47.6362529437834 | 6.13220138624202 | 0.049482850875851 | 830.335475158381 | 6.70026372362348 |
| United States of America | 2045 | 40 to 44 | 2377.84393263722 | 19.3702096326377 | 15289.0574659409 | 124.546545774459 | 18.5130641999926 | 0.150809701837168 | 2170.8788984228 | 17.6842469652254 |
| United States of America | 2045 | 45 to 49 | 3224.92776460768 | 27.1981122367484 | 28233.2547692812 | 238.111141728795 | 40.4851800615212 | 0.341440352035972 | 4083.46029379785 | 34.438728396923 |
| United States of America | 2045 | 50 to 54 | 6402.2173020404 | 53.3613136109355 | 50156.7732614579 | 418.047557814737 | 83.9679803454207 | 0.699857802555511 | 7357.97084911016 | 61.3273451206282 |
| United States of America | 2045 | 55 to 59 | 11214.1477040378 | 97.4547025318923 | 89642.7982311694 | 779.02596490677 | 160.257868645272 | 1.39269459698633 | 12878.9819356636 | 111.922670057377 |
| United States of America | 2045 | 60 to 64 | 23604.1313269632 | 220.137296183303 | 160300.511385625 | 1494.99766224916 | 301.477094247284 | 2.81164138046397 | 21067.9878317901 | 196.484666733536 |
| United States of America | 2045 | 65 to 69 | 40526.6224573337 | 416.308388263642 | 296609.402599449 | 3046.91027410485 | 584.786845159127 | 6.0072035176946 | 36547.0263119227 | 375.428118535256 |
| United States of America | 2045 | 70 to 74 | 58954.3368345073 | 647.361986258129 | 509277.15424771 | 5592.23778659676 | 1262.57472865719 | 13.863999292151 | 62768.9641358714 | 689.249400131981 |
| United States of America | 2045 | 75 to 79 | 77799.9181196881 | 905.156361501311 | 801350.260539677 | 9323.2397108997 | 2667.71842108264 | 31.0373372864273 | 100684.806035662 | 1171.40859389468 |
| United States of America | 2045 | 80 to 84 | 69359.1705354857 | 878.212751552031 | 1082718.35653475 | 13709.1758697113 | 6189.89852503729 | 78.3753290809562 | 153877.845524523 | 1948.37229277202 |
| United States of America | 2045 | 85 to 89 | 35822.7783340823 | 601.645282104445 | 1008036.93813598 | 16930.0287755601 | 10144.9512865153 | 170.384943953502 | 170438.450186964 | 2862.52195426782 |
| United States of America | 2045 | 90 to 94 | 13971.6028838087 | 419.835188784226 | 615809.342590935 | 18504.5648485592 | 13258.6598785201 | 398.411837168312 | 156753.851138612 | 4710.32445115512 |
| United States of America | 2045 | 95 plus | 4153.53985116569 | 326.630135020429 | 230801.05693139 | 18149.9595741692 | 9212.88174148838 | 724.491617988225 | 90353.1482801127 | 7105.27937127449 |

AF/AFL, atrial fibrillation and atrial flutter; DALYs, disability-adjusted life years; ASIR, age-standardized incidence rate; ASPR, age-standardized Prevalence rate; ASDAR, age-standardized DALYs rate; ASDR, age-standardized Deaths rate;

**Supplemental Figure1** Temporal trends age-standardized rates of incident cases (A), prevalence(B), disability-adjusted life years cases (C), deaths (D) of AF/AFL in mainland China from1990 to 2021.

**
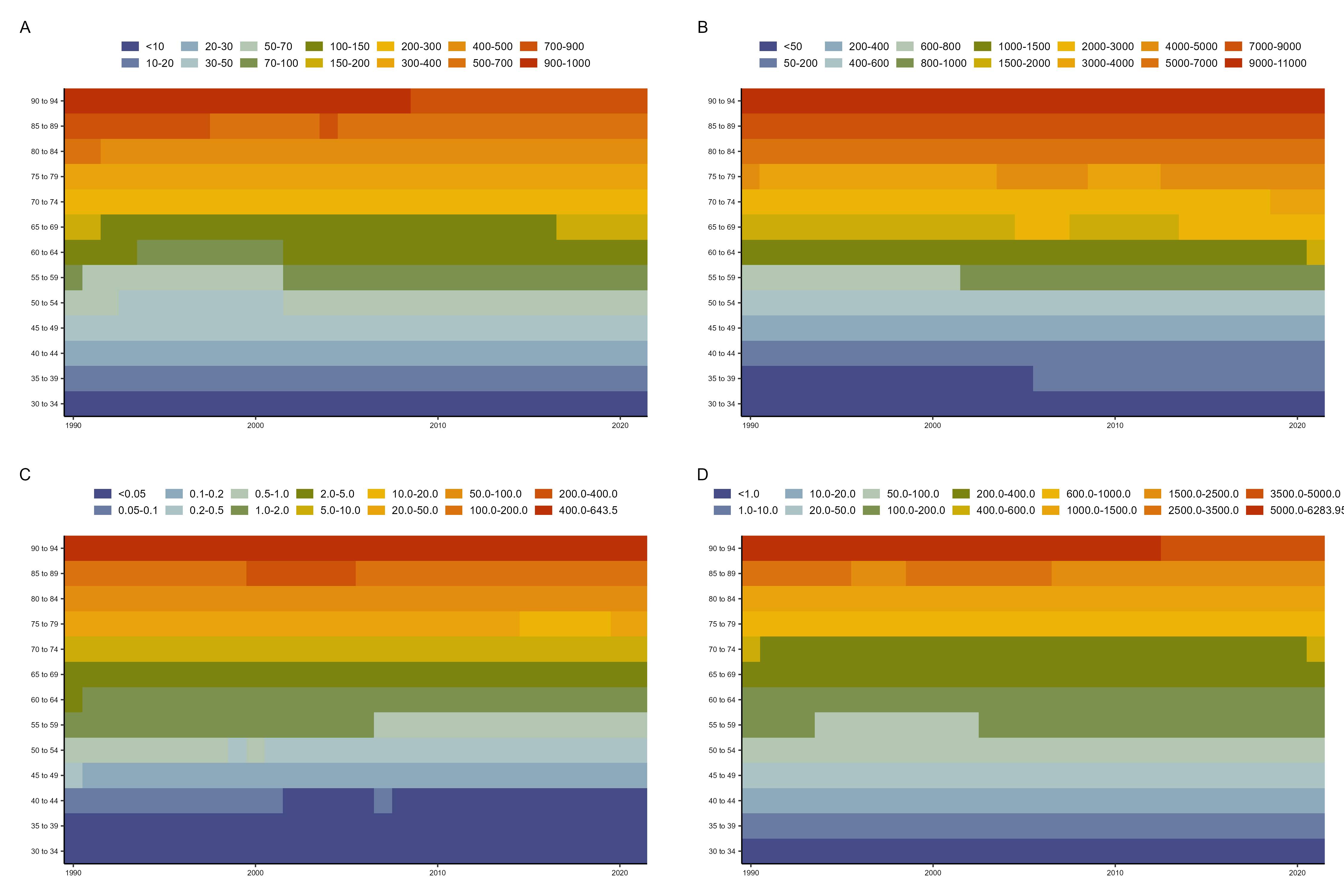
**

**Supplemental Figure2** Prediction of future burden of atrial fibrillation/atrial flutter in different age groups in mainland China in all population.

**Supplemental Figure3** Prediction of future burden of atrial fibrillation/atrial flutter in different age groups in mainland China in Male.

**Supplemental Figure4** Prediction of future burden of atrial fibrillation/atrial flutter in different age groups in mainland China in Female.
